# Supplementary material for: A study protocol to investigate if acipimox improves muscle function and sarcopenia: an open-label, uncontrolled, before-and-after experimental medicine feasibility study in community-dwelling older adults
Source: BMJ Open. 2024 Feb 27;14(2):e076518. doi: 10.1136/bmjopen-2023-076518 (PMC10900389; doi:10.1136/bmjopen-2023-076518)
Supplement: Supplementary data [file bmjopen-2023-076518supp001.pdf]

Participant ID: A C - __ __ __

Acipimox to improve muscle function and sarcopenia – a feasibility study

EudraCT 2021-000993-28 Sponsor ref: 09768

Prescreening form

Centre/Participant ID: | A | C | – | | | |

PRESCREENING CONTACT

Date of prescreening contact | d | d | m | m | y | y | y | y |

Contact by: Telephone ☐ Face to face ☐

Age (years) ☐☐

Sex: Male ☐ Female ☐

SARC-F:

	Score
How much difficulty do you have in lifting and carrying 10 pounds? *(none=0, some=1, a lot or unable without help=2)*	
How much difficulty do you have walking across a room? *(none=0, some=1, a lot or unable without help=2)*	
How much difficulty do you have transferring from a chair or bed? *(none=0, some=1, a lot or unable without help=2)*	
How much difficulty do you have climbing a flight of 10 stairs? *(none=0, some=1, a lot or unable without help=2)*	
How many times have you fallen in the past year? *(none=0, 1-3 = 1, 4 or more = 2)*	
**Total score**	

McDonald C, *et al. BMJ Open* 2024; 14:e076518. doi: 10.1136/bmjopen-2023-076518

Participant ID: A C - __ __ __

Active medical diagnoses

	Yes	No
Chronic heart failure		
Severe COPD (GOLD stage IV)		
Progressive neurological disease with life expectancy <6 mths		
Cancer with life expectancy <6 mths		
Myositis or other myopathy		
Active thyrotoxicosis		

Concomitant medications

	Yes	No
Statin		
Fibrate		
Acipimox		
Niacin		
>7.5mg/day oral prednisolone or equivalent		
Antiplatelet other than aspirin 75mg once daily		
Anticoagulants		
Previous intolerance of acipimox or niacin		

Weight (kg) | | | . |

Weight change in last 6 months (to nearest kg) (NB: 1 stone = 6kg): | +/- | | |

Participant ID: A C - __ __ __

Other eligibility criteria:

	Yes	No
Contraindications to MRI		
>10% weight loss over last 6 months		
Unwilling to have two muscle biopsies		

	Yes	No
Eligible to proceed in study?		
Participant happy for study team to access medical records?		
Participant happy to receive PIS?		
PIS sent?		

Research nurse / investigator signature: __________________________ Date: _ _ / _ _ /_ _ _ _

McDonald C, *et al. BMJ Open* 2024; 14:e076518. doi: 10.1136/bmjopen-2023-076518
