# Supplementary material for: A study protocol to investigate if acipimox improves muscle function and sarcopenia: an open-label, uncontrolled, before-and-after experimental medicine feasibility study in community-dwelling older adults
Source: BMJ Open. 2024 Feb 27;14(2):e076518. doi: 10.1136/bmjopen-2023-076518 (PMC10900389; doi:10.1136/bmjopen-2023-076518)
Supplement: Supplementary data [file bmjopen-2023-076518supp002.pdf]

Acipimox to improve muscle function and sarcopenia – a feasibility study

Introduction

Thank you for considering taking part in the acipimox study. Before you decide whether to take part, please take time to read this information about the study carefully. Feel free to discuss it with your family, friends, carers or your GP if you wish to do so. If anything is unclear, or you need more information, please ask. You will find our contact details at the back of the information. This leaflet is yours to keep.

SUMMARY of the ACIPIMOX STUDY

**Purpose of the study**	• We would like to find out whether a medicine called acipimox (used to treat high cholesterol) can help to improve muscle function for people over the age of 65 with muscle weakness.
**Will I definitely get acipimox?**	• Yes. Everyone in the study will receive acipimox – no dummy (placebo) medication is used in this study.
**What are the side effects of the medicine?**	• Acipimox may cause some people to feel hot and red in the face (flushing) or cause a headache at the start of treatment but this usually goes away quickly. We will give you a mini-aspirin each day to stop this side-effect. Acipimox can cause nausea or indigestion in some people. A full list of side-effects are given later in this information sheet under Supporting Information
**If I take part, what will I have to do?**	• All of the study visits will take place in a research centre (the Clinical Ageing Research Unit)<br>• You will be asked to attend a screening visit. The doctor will discuss the study with you and you can ask questions. We will ask to take a small blood sample to see if it is safe for you to take part.

McDonald C, *et al. BMJ Open* 2024; 14:e076518. doi: 10.1136/bmjopen-2023-076518

- If the study is suitable for you, we will ask you to take part in another four visits.
- We will ask you to take a tablet (acipimox) 2-3 times a day for 18 days together with aspirin once a day.
- At two of the visits, we will take a small sample of muscle tissue (a biopsy) from your thigh
- At two of the visits, we will do an MRI scan of your leg some of the scan will be at rest and other parts will be whilst you exercise your leg muscle in the scanner
- At these visits we will also ask you to do some simple tests of muscle strength and take a blood sample We will ask you to give small blood sample (a tablespoons worth)
- We will ask you to wear a sensor to measure your walking for a week before and after taking the course of tablets

Do I have to visit the hospital to take part?

- The study visits are at a research centre which is separate from the hospital. For this study you do need to come to the research centre.

Do I have to take part?

- No – it is entirely your choice whether you wish to take part or not. Your care will not be affected in any way.

If you are still interested in taking part in the study, please read on for more information.

Newcastle University

NHS The Newcastle upon Tyne Hospitals NHS Foundation Trust

What is the purpose of the study?

Many of us lose muscle size and strength as we get older – this is called sarcopenia. People with weaker muscles are more likely to fall over and may start to have problems carrying out normal daily activities. They may also take longer to recover from other illnesses. Falling, struggling with daily living and taking a long time to get better from being unwell is a condition called frailty.

The best way to keep up your muscle strength is to do strengthening exercises, but not everyone wants to, or is able to do these.

Acipimox is a medicine that is already safely used in older people to treat high cholesterol. Recent research suggests that levels of a naturally occurring substance called Nicotinamide Adenine Dinucleotide (NAD) are low in the muscles of people with sarcopenia. All cells in the body need NAD to produce and use energy. We think that acipimox might be able to increase the levels of NAD in muscle, which could in turn help muscles to work better for people with sarcopenia. To know if this is correct, we need to test the effects of acipimox on muscle function in people with signs of muscle weakness.

What are the possible side effects of the study medicine?

As with any medicine, the medicine used in this study (**acipimox**) may cause side effects in some people. Doctors around the world have used acipimox for nearly 40 years and the side effects are well understood.

In some people, acipimox can cause flushing (redness of the face) and headache. This side effect usually affects people for the first few doses but then gets better. To minimise this side effect, we will give you a low dose of aspirin to take once a day (if you already take aspirin, you will just keep taking your normal dose of aspirin). Less commonly, acipimox can cause nausea or indigestion. To help prevent this, we will ask you to take your study medicine during or just after meals.

Aspirin can cause inflammation of the stomach lining, and in rare cases can cause stomach ulcers. We are using a low dose of aspirin over a short period of time to minimise side effects, but if you have indigestion, stomach ulcers or are prone to bleeding, we will not include you in the study.

McDonald C, *et al. BMJ Open* 2024; 14:e076518. doi: 10.1136/bmjopen-2023-076518

Newcastle University

NHS The Newcastle upon Tyne Hospitals NHS Foundation Trust

The 28-day follow-up visit is the last study visit. For the 28 days following on from this final visit, we would like to know if you have been unwell. We will ask you to contact your study doctor or a member of their team to tell them about this. You can contact your study doctor and team on the contact details given on the last page of this information sheet.

If you are unsure whether the study doctor needs to know about an illness or not, please get in touch with your study doctor or a member of their team to check.

What are the benefits and disadvantages of taking part in this study?

We cannot promise the study will help you directly. However, the information we collect from this study may help to improve the treatment for older people with muscle weakness. If you want to find out more about taking part in research studies, you can visit the NHS Choices website www.nhs.uk.

If you do get a side effect from the study medicine, your doctor can stop your medicine. The side effects should disappear rapidly.

The muscle biopsy will be taken after your skin and surrounding area is numbed by injecting local anaesthetic. The injection and biopsy may cause some minor discomfort and bruising after biopsy and may be uncomfortable for a few days. Muscle biopsy is a simple procedure removing only a small amount of muscle with a low risk of complications. In very rare cases permanent muscle weakness, infection or a patch of numbness might occur. See page 7 for more information.

Is it safe for me to take part in this study during COVID-19?

The study will only take place if Newcastle Hospitals says that it is safe to do so. The study team will follow all of the hospital COVID-19 policies and procedures when you visit, including wearing masks, gloves and visors. These will be explained to you when your appointments are arranged. The visits will take place at the Clinical Ageing Research Unit, which is on the old Newcastle General Hospital site. This means that you will not have to mix with other patients in the main hospitals.

McDonald C, *et al. BMJ Open* 2024; 14:e076518. doi: 10.1136/bmjopen-2023-076518

What would I have to do if I took part?

We will ask you to attend five visits at the Clinical Ageing Research Unit, on the old Newcastle General Hospital site:

- Screening visit
- First baseline visit (on day 0)
- Second baseline visit (on day 7)
- First follow-up visit (on day 21)
- Second follow-up visit (on day 28)

These visits are extra, and not part of your usual NHS care. There is a flow diagram (Figure 1) on page 10 that shows the details of the trial visits.

Screening Visit

At the screening visit the doctor will discuss the study with you, and you will be able to ask questions. This visit may take place in your home rather than in the research unit if you prefer. We will ask if you are happy to give written consent to take part in the study. We will ask you some questions about yourself, your medications and your health. We will also ask to take a small blood sample (2 teaspoons) to make sure that it is safe for you to take part in this study. We will time you walking a short distance (4 metres), ask you to stand and sit from a chair five times, and test your handgrip strength.

When we have the results of the blood test, we will call you and arrange the next visit.

Baseline Visit 1

At this first visit, we will do the following:

- Measure your weight and height.
- Time you walking a short distance (4 metres), with your usual walking aids.
- To test your balance, we will ask you to stand beside the researcher for a short time (no more than 30 seconds).

McDonald C, *et al. BMJ Open* 2024; 14:e076518. doi: 10.1136/bmjopen-2023-076518

- Time you standing and sitting from a chair five times.
- Test the strength of your handgrip on both hands.
- Ask you questions about your activity level, weight change and levels of tiredness.
- Take a small amount of blood (6 teaspoons).
- Ask about other medicine that you may be taking.

We will then perform a magnetic resonance imaging (MRI) scan of your leg muscles whilst you exercise your leg.

The Magnetic Resonance Imaging (MRI) scan

You will need to arrive at the MRI centre 30 minutes before your arranged scan. Before your scan you will be asked questions to ensure that there are no known reasons why you should not undergo a scan, for example if you have a pacemaker fitted. For your scan you will be asked to remove items of jewellery, clothing with metal and all other metal (such as hair grips). For this reason you may prefer to leave valuable items such as jewellery at home, however lockers are available. You may also prefer to arrive at the MRI centre wearing loose fitting clothing, although if you prefer to change into comfortable clothing at the clinic changing rooms are available.

When you go through the MRI scanner, you will be asked to lie down on the scanner bed. We slide the bed into the scanner up to chest height and collect images of your thigh muscle. You will be asked to keep as still as possible while we do this so that we get good images. The scanner is noisy but we will provide earplugs. Each of these scans lasts about 1 minute.

We will then move the table so that your calf is in the middle of the scanner. We will collect some images of your calf muscles at rest, again you will be asked to keep as still as possible. We will then ask you to push against a pedal (an up down motion) for about 3 minutes. The effort required is similar to walking uphill on Dean Street (a steep hill in Newcastle). The radiographer will then ask you to stop pushing and keep still for about 6 minutes while we collect data from your leg muscle after the exercise. After this, your MRI scan is complete.

When having a scan, there is always a small possibility that an abnormality could be observed on the images of which you and your doctors were unaware. The MRI scans we collect will be reviewed by a radiologist in the

Newcastle upon Tyne Hospitals NHS Foundation Trust to look for any such findings.

It is important to recognise that the MRI scans are not being taken for diagnostic purposes and so there is no guarantee that the scans would be of the right kind to detect any abnormality which may be present.

Should the radiologist suspect anything abnormal on your scans they will inform the study Principal Investigator who will contact your clinical care team or GP in order to make recommendations about any further investigations which may be appropriate for them to arrange.

The walking sensor

After the scan, we will give you the sensor to wear. This is small, lightweight and is worn on the lower back. You will need to wear this all the time for the next seven days until you return for the second baseline visit. You do not need to take it off at night, and you can wear it in the shower. It will monitor your walking activity.

Baseline visit 2

At the second baseline visit, which will take place 7 days after the first visit, we will collect the accelerometer and then do a muscle biopsy. If you usually take aspirin we will ask you not to take this on the morning of your muscle biopsy.

The muscle biopsy:

This test involves taking a small tissue sample from one of your thigh muscles. You will meet with an experienced doctor who will explain about the muscle biopsy and check that you are willing to have this procedure performed. Throughout the study you are free to change your mind about taking part in any part of the study without having to give a reason why.

If you are willing to go ahead, the doctor will ask you questions about what medication you are currently taking, your general health and examine you. This is because if, for example, you had varicose veins over the site of where we would take the biopsy, or were taking medications that can cause bleeding, then it would not be safe to perform this and therefore you would not undergo this part of the study.

Muscle biopsy is a simple procedure removing only a small amount of muscle with a low risk of complications. One in a hundred people experience significant bleeding that may leave you with a bruise taking a few weeks to disappear. In very rare cases permanent muscle weakness, infection or a patch of numbness (one in a thousand people) might occur.

The doctor and the nurse helping with the muscle biopsy will take you to a treatment room, set up to take a small tissue sample (biopsy) from your thigh muscle.

The procedure takes no longer than 30 minutes. Whilst lying down, you will be given a local anaesthetic injection to numb the skin and surrounding area. When the area is numb, a small cut is made in the skin to allow the biopsy to be taken. The muscle biopsy will be done with a special type of forceps as shown in the photo, below. The local anaesthetic may cause a stinging sensation for a few seconds and you may feel a pulling sensation when the biopsy is taken.

The wound is so small that it will not require stitching but is closed using medical tape called “Steri-strips”. A plaster will then be applied to the area, and a tight bandage wrapped around your leg for two hours whilst you rest on a bed. This is done to reduce the chance of bruising and bleeding.

The biopsy site may be uncomfortable for a few days, if so, paracetamol or codeine is recommended for pain relief. Anti-inflammatory drugs such as aspirin or ibuprofen should be avoided in the first 48 hours. You should also avoid getting the area wet in the first 48 hours. The Steri-strips can be removed after 5 days.

You should not drive after the biopsy and again we will provide a taxi to take you home. You will be given an advice sheet informing you to avoid strenuous activities like running or weightlifting for the first 5 days after your biopsy as this may delay the healing process and result in bleeding. However, the biopsy should not limit routine household tasks.

Follow-up phone calls

A member of the study team will telephone you twice as follow-up: the first time one or two days after your muscle biopsy and the second time around a week later to ensure that you are well. They will check with you that the wound from the muscle biopsy has healed. If you have any concern at all

about the site of the muscle biopsy, then a member of the study team will arrange to visit you at home.

The study medication

At the end of this second baseline visit, we will give you the study medicines to take home with you. **Please wait until two days after the biopsy** before you take the first doses of medication; we will telephone you first to make sure that the biopsy wound is healing as it should before starting the medication.

Follow up visit 1

At the first follow-up visit, which will take place two weeks later, we will repeat all the tests (including the MRI scan) which we did at the first baseline visit.

Follow up visit 2

At the second follow-up visit, which will take place seven days after the first follow-up visit, we will collect the accelerometer and your study medications. We will then do the muscle biopsy again, and as before we will telephone you a couple of days later and then a week later to check that everything has healed without problems.

A summary of the study visits is shown in a flow diagram (Figure 1) on the next page.

McDonald C, *et al. BMJ Open* 2024; 14:e076518. doi: 10.1136/bmjopen-2023-076518

Newcastle University

The Newcastle upon Tyne Hospitals NHS Foundation Trust

Figure 1. Summary of acipimox study visits

Screening visit – 60 mins
Written informed consent with doctor
Medical history, other medicines
Physical tests (short walk, sit to stand and handgrip strength)
Blood sample (2 teaspoons)
After visit - eligibility for the study confirmed

Day 0: Baseline visit 1 - two hours
Height and weight
Physical tests (walking, balance, sit to stand and handgrip strength)
Questions on activity and tiredness
Blood sample (6 teaspoons)
Review of other medication and any episodes of ill health
MRI scan of leg
Start wearing walking sensor

Day 7: Baseline visit 2 – three hours (includes 2 hours rest after the biopsy)
Medicines review and review of any episodes of ill health
Return walking sensor
Muscle Biopsy
Receive study medication
After visit: telephone call at 48 hours and 7 days. Start taking medication at 48 hours

Day 21: Follow up visit 1 - two hours
Physical tests (walking, balance, sit to stand and handgrip strength)
Questions on activity and tiredness
Blood sample (6 teaspoons)
Review of other medication and any episodes of ill health
MRI scan of leg
Start wearing walking sensor

Day 28: Follow up visit 2 – three hours (includes 2 hours rest after the biopsy)
Medicines review and review of any episodes of ill health
Return walking sensor and study medication
Muscle Biopsy
After visit: telephone call at 48 hours and 7 days

Newcastle University

NHS The Newcastle upon Tyne Hospitals NHS Foundation Trust

What happens to my blood and muscle samples?

At the baseline and follow up visits, we take blood and muscle samples to analyse as part of the study. If there is any sample left over from these analyses, we ask your permission to store it in a licenced Newcastle University Biobank. Your consent to store these additional samples in the biobank is optional. Your biobank samples may be used in further research linked to this study. They may also be used by other researchers in different research studies. Your samples will be identified only using your unique study identity number. Researchers who use your samples will not know who you are. Other researchers may want to use parts of your biobank sample in animal or commercial (paid) research. You can give samples to the biobank but opt out of them being used for animal or paid research.

Pregnancy

We know that it is not possible for female participants over the age of 65 years to become pregnant.

However, if you are a male participant, we will ask you to inform us if your female partner becomes pregnant, or is breast-feeding an infant. We will ask for your consent to do this. We will also ask your partner to sign a consent form. This will allow the study team to collect safety information about their pregnancy and their baby.

What happens at the end of the study?

At the end of the study (4 weeks) you will stop taking the study medicines. You will continue to receive standard care like any other patient with your condition under the care of your GP and/or hospital doctor. If you were taking aspirin before the study, you will continue to take this as before.

When everyone has completed the study, we will analyse the results and we will tell you what the results are. We can either invite you to a study event to which we will invite you, or send you a written newsletter – whichever you prefer.

Newcastle University

NHS The Newcastle upon Tyne Hospitals NHS Foundation Trust

Who do I contact for further information?

We will be happy to answer any questions you, your family or your carers may have about any aspect of this clinical study. Please call the number at the end of this booklet to speak to the research team.

McDonald C, *et al. BMJ Open* 2024; 14:e076518. doi: 10.1136/bmjopen-2023-076518

SUPPORTING INFORMATION

Acipimox side effects

Very common – Flushing, headache, indigestion

Common – Stomach ache, tiredness, rash

Uncommon – Allergic reaction, rash or itching, wheezing, nausea, feeling hot or unwell, muscle aches

Other – loose stools, gritty eyes

Acipimox may cause a sensation of heat, flushing or itching, especially at the beginning of treatment; it may cause a rash or redness of the skin. These reactions usually disappear rapidly during the first day of treatment.

Why was I contacted?

We have contacted you because you are aged 65 or over and our measurements or questionnaire results suggest that your muscles are not as strong as they used to be. This trial is testing a medicine (acipimox) to see if we can prevent further weakening of muscles in people like you.

Do I have to take part?

No - it is up to you to decide whether or not to take part in this study. You do not have to take part. If you choose not to, you will continue to get the standard care arranged by your doctor.

If you agree to take part, we will ask you to sign a consent form. We will give you a copy of your signed consent form which is yours to keep. You can still change your mind and withdraw at any time without having to give a reason. If you decide not to take part, or withdraw from the study later on, your current or future medical care will not be affected in any way.

What does giving consent mean for me?

By signing a consent form, this means that you fully understand what taking part in the study means for you. That’s why it is really important that you take as much time as you want to read this information sheet. Feel free to discuss the study with your family, friends or any healthcare professional. At the screening visit, you will be discuss the study with a study doctor, who will answer all of your questions.

Will I know what treatment I am on?

Yes. Everyone in the study will receive acipimox – no dummy (placebo) medication is used in this study.

Who has checked the scientific quality of the study?

Independent experts at Newcastle University have checked the quality of the science used to plan this study. The study has also been checked and approved by the app committee ensures that when you take part in the study, your rights and wellbeing will be protected. The study has also been checked by the government Medicines and Healthcare products Regulatory Agency (MHRA). The MHRA are responsible for approving all studies involving medicines. The Health Research Authority gives final overall approval for the study. The Newcastle upon Tyne Hospitals NHS Foundation Trust is the study Sponsor, which means that they have overall responsibility for the study. The Sponsor has carefully checked all of the study documentation. The Sponsor has also assessed the risks of this study. This is to ensure that we are not doing anything harmful to you during the study and that your information is collected safely and stored securely.

What happens if relevant new information becomes available?

During the course of the study, if new information on the risks or benefits of acipimox becomes available, we will let you know at your next study visit. If this new information requires urgent action, we will contact you before your next visit. If necessary, we will then discuss whether you should or would like to withdraw from the study.

Will you tell my GP that I'm taking part in a clinical study?

Yes. With your permission, we will write to your GP to tell them that you are taking part in this study. Your hospital medical record will also show that you are taking part in a clinical study. It is important for your safety that that your GP practice and hospital medical records show that you took part in a clinical study. If we discover a new health problem during the study, we will tell you. With your permission, we will also tell your GP. Any blood test results from taking part in this study will also be added to your medical records. Your GP will be asked to let the study team know of any side effects from taking the medicine or if you have had any emergency hospital admissions.

Who has overall responsibility for the study?

The study Sponsor is the Newcastle upon Tyne Hospitals NHS Foundation Trust, who have overall responsibility for the study.

The doctor in charge of the study (the Chief Investigator) is Professor Miles Witham, a Consultant Geriatrician based in Newcastle upon Tyne.

Who is providing the study medications?

Acipimox and aspirin have been on the market for many years. We have bought the study medications from their manufacturers in the same way that the NHS would usually buy medications – the medications have not been supplied as a favour by the manufacturers.

What will happen to the results of the Acipimox study?

- The results will be published in medical journals and presented at meetings to other doctors, nurses, researchers and patients.
- A report will be written for the study funder.
- A report will be written for the Controlled Clinical Trials (ISRCTN) database.
- All study results that are published will be anonymous. This means that no-one will be able to find out who you are. Your identity will always be protected.
- The results will be available at the end of the study through publications, in the wider press and directly to patient groups.
- Fully anonymised data may be made available to other researchers to help inform other research studies.

What if I have a complaint or a problem occurs?

a) Complaints

If you have any concern or complaint about any aspect of this clinical study, please contact your local study team by phone, letter or email. Their contact details are listed at the end of this information sheet. If you are still unhappy and wish to raise your concerns with someone who is not directly involved in your care, you can contact Patient Advice and Liaison Service (PALS) for

McDonald C, *et al. BMJ Open* 2024; 14:e076518. doi: 10.1136/bmjopen-2023-076518

confidential advice on any aspect of care on 0800 032 0202, or email northoftynepals@nhct.nhs.uk

b) Harm

In the unlikely event that you are harmed during the study and this is due to NHS staff neglect, you may have grounds for legal action and compensation. This is organised through the NHS Indemnity (insurance) scheme. You may need to pay for your own legal costs. NHS Indemnity does not offer no-fault compensation (for harm that is not anyone's fault).

Newcastle University also have indemnity arrangements. This covers Newcastle University staff involved in designing and managing the Acipimox study.

Will my taking part in the study be kept confidential?

Yes. All the information that you provide during the course of this study will be securely stored. Paper copies of your study information will be stored in locked files or rooms at your local hospital. Electronic copies of your study information will be stored on a secure, password-protected computer database. Only authorised members of the study team will be granted access to the database.

- At study visits, your name will not be written on completed test forms or questionnaires. Instead, we will use a study code number (called a Participant Unique Study Identifier). This number is unique for you. No-one else taking part in the study will have this number. This number will also be used in the study database. Only the study team at your hospital will be able to link this number back to you using your date of birth, name and NHS number
- The study team at your hospital will have access to your information during the study. They will use this information to contact you to organise study visits as well as for ongoing safety.
- You will not be named in any results, reports or on websites
- Very occasionally, information might be given during the study that, by law, we must pass on to others. For instance, information which suggested you or others were at risk of harm. In this case, confidentiality

would be broken so that we could pass this information to the relevant people. You would be informed of this.

- At the end of the study, all study information will be kept in a secure storage area for at least 15 years. This is called archiving. Archiving means that any queries about the running of the study can still be answered after the study has ended. All information will be held securely to make sure we protect your confidentiality. After the archiving period has ended, your information will be safely destroyed.
- If there are any unexpected serious side effects to the medicine, we would send details of this to the government medicines agency (MHRA). There is a specific form to do this, and only your study number will be sent to them.

Will you look at information from my existing medical records?

Yes. The study team at your hospital will be able to look at your GP and hospital medical records. They need to do this to collect information that is needed for you to take part in the study. For example, they will collect results of your blood tests, and prescriptions and health history.

Authorised people from Newcastle Hospitals NHS Foundation Trust or from the MHRA will also need to look at your medical records. This is to check that the study is being carried out to the correct standards. Everyone who looks at your medical records will have a duty of confidentiality to you as a research participant.

What will happen if I don't want to carry on with the study?

You can withdraw from the study completely at any time, for any reason. You do not have to tell the study team why you want to withdraw. You will always be fully cared for and supported in line with your GP and hospital's standard practice.
If you do give a reason for withdrawing from the study, we will ask if you are happy for us to record why you decided to withdraw.

If you withdraw from the study, we will keep the information about you that we have already collected. You are free to request that the study team

destroys all information donated by you. By destroying your information, this means that it cannot be used at all for the remainder of the study. However, if some of your information has already been used in calculations and reports, it would not be possible to remove that information from these.

If I stop taking the medicine, do I have to leave the trial?

If you become unwell or the medicine causes side effects that you cannot tolerate, your usual doctor or the study team may ask you to stop taking the study medicine. If you stop taking the medication, you will not continue in the trial and will not have the second set of scans, blood tests and muscle biopsy.

What happens if I lose the capacity to consent during the study?

During the study, if you lose the capacity to make your own decisions, we will stop your medicine and withdraw you from the study.

Who is funding the acipimox study?

The Medical Research Council are funding the study through money given to Newcastle University. The UK government funds the MRC to carry out research to improve knowledge of diseases, and treatments to improve health.

How have patients and the public been involved in the design of the study?

Volunteers from the local VOICE organisation (https://www.voice-global.org/) have given us feedback on designing studies for older people which we have used in designing this study and the information sheet.

Will my expenses be reimbursed?

Yes – we will pay for your travel expenses including providing a taxi if you need this to attend study visits at the hospital. Alternatively, transport may be arranged for you if your local hospital is able to offer this. The study team will manage any payments to reimburse costs to you and you may be asked to provide receipts for your travel.

Will I be paid for taking part?

We will not give you a payment for taking part, but we will pay for your transport and make sure that you have some food and drink at each trial visit.

HOW WILL WE USE INFORMATION ABOUT YOU?

We will need to use information from you, your medical records and your GP for this clinical research study.

This information will include your initials, date of birth, NHS number, name and contact details. People will use this information to do the research or to check your records to make sure that the research is being done properly.

People who do not need to know who you are will not be able to see your name or contact details. Your data will have a code number, called your unique study identifier, instead.

We will keep all information about you safe and secure.

Once we have finished the study, we will keep some of the data so we can check the results. We will write our reports in a way that no-one can work out that you took part in the study.

What are your choices about how your information is used?

- You can stop being part of the study at any time, without giving a reason, but we will keep information about you that we already have.
- We need to manage your records in specific ways for the research to be reliable. This means that we won't be able to let you see or change the data we hold about you.

Where can you find out more about how your information is used?

You can find out more about how we use your information:

- www.hra.nhs.uk/information-about-patients/
- our leaflet available from https://www.newcastle-hospitals.nhs.uk/help/privacy/

- by asking one of the study team
- by sending an email to the trial Sponsor Data Protection Officer at nuth.dpo@nhs.net

Further Information and contact details

If you have any further questions or would like further information about the study or rights of participants, please feel free to contact the people below.

They are also who you or a doctor should contact in the event of an emergency.

Professor Miles Witham, telephone 07555 450498

Clinical Ageing Research Unit, telephone 0191 208 1250

Thank you for reading this information sheet

This research is funded by the UK Medical Research Council through their Confidence in Concept funding stream. The views expressed are those of the author(s) and not necessarily those of the Medical Research Council.
