# Supplementary material for: A study protocol to investigate if acipimox improves muscle function and sarcopenia: an open-label, uncontrolled, before-and-after experimental medicine feasibility study in community-dwelling older adults
Source: BMJ Open. 2024 Feb 27;14(2):e076518. doi: 10.1136/bmjopen-2023-076518 (PMC10900389; doi:10.1136/bmjopen-2023-076518)
Supplement: Supplementary data [file bmjopen-2023-076518supp003.pdf]

Supplementary Table 1 Schedule of events

	Visit 1	Visit 2	Visit 3	Visit 4*	Visit 5	Visit 6	Visit 7	Visit 8	Visit 9	Visit 10
	**Pre-Screen**	**Screening**	**Baseline 1**	**Baseline 2**	**Phone call**	**Phone call**	**Follow up 1**	**Follow up 2**	**Phone call**	**Phone call**
**Assessment**		**Day -28 to -1**	**Day 0**	**Day 7 (+/- 1 day)**	**Day 9 (+/- 1 day)**	**Day 14 (+/- 2 days)**	**Day 21 (+/- 3 days)**	**Day 28 (+/- 3 days)**	**Day 30 (+/- 3 days)**	**Day 37 (+/- 4 days)**
SARC-F questions	X									
Demographics	X	X								
Medical history	X	X								
Concomitant medications	X	X	X	X			X	X		
Informed written consent		X								
Height		X								
Weight		X								
Bloods (U+Es, liver function tests)		X								
Bloods (Full blood count)		X								
Bloods (research bloods for NAD levels)			X				X			
Frailty questions (activity, exhaustion, weight loss)			X				X			
Muscle biopsy				X				X		
MRI scanning			X				X			

Accelerometer given out			X				X			
Accelerometer returned				X				X		
Medication dispensing				X						
Instructions on taking medication					X				X	
Medication adherence (collection/count)								X		
Telephone check of wound healing					X	X			X	X
Adverse Events log completion		X	X	X	X	X	X	X	X	X
4m walk speed		X								
5-times sit to stand		X								
Grip strength		X	X				X			
Short Physical Performance Battery			X				X			
Eligibility confirmation		X								

BMJ Publishing Group Limited (BMJ) disclaims all liability and responsibility arising from any reliance placed on this supplemental material which has been supplied by the author(s)
