# Supplementary material for: A study protocol to investigate if acipimox improves muscle function and sarcopenia: an open-label, uncontrolled, before-and-after experimental medicine feasibility study in community-dwelling older adults
Source: BMJ Open. 2024 Feb 27;14(2):e076518. doi: 10.1136/bmjopen-2023-076518 (PMC10900389; doi:10.1136/bmjopen-2023-076518)
Supplement: Author's manuscript [file bmjopen-2023-076518.draft_revisions.pdf]

BMJ Open

BMJ Open is committed to open peer review. As part of this commitment we make the peer review history of every article we publish publicly available.

When an article is published we post the peer reviewers' comments and the authors' responses online. We also post the versions of the paper that were used during peer review. These are the versions that the peer review comments apply to.

The versions of the paper that follow are the versions that were submitted during the peer review process. They are not the versions of record or the final published versions. They should not be cited or distributed as the published version of this manuscript.

BMJ Open is an open access journal and the full, final, typeset and author-corrected version of record of the manuscript is available on our site with no access controls, subscription charges or pay-per-view fees (http://bmjopen.bmj.com).

If you have any questions on BMJ Open's open peer review process please email info.bmjopen@bmj.com

BMJ Open

Acipimox to improve muscle function and sarcopenia – Protocol for an open-label, uncontrolled, before-and-after experimental medicine feasibility study.

Journal:	*BMJ Open*
Manuscript ID	bmjopen-2023-076518
Article Type:	Protocol
Date Submitted by the Author:	09-Jun-2023

Complete List of Authors:	McDonald, Claire; Newcastle University, AGE Research Group, Translational and Clinical Research Institute, Faculty of Medical Sciences; Newcastle Upon Tyne Hospitals NHS Foundation Trust, NIHR Newcastle Biomedical Research Centre<br>Alderson , Craig; Newcastle Upon Tyne Hospitals NHS Foundation Trust<br>Birkbeck, Matthew; Newcastle University, Newcastle Magnetic Resonance Centre Translational and Clinical Research Institute, ; Newcastle Upon Tyne Hospitals NHS Foundation Trust<br>Brown, Laura ; Newcastle University, Wellcome Centre for Mitochondrial Research, Translational and Clinical Research Institute<br>Del Din, Silvia; Newcastle University, Brain and Movement Research Group, Translational and Clinical Research Institute,<br>Gorman, Grainne; Newcastle University, Wellcome Centre for Mitochondrial Research, Translational and Clinical Research Institute<br>Hollingsworth, Kieren; Newcastle University, Newcastle Magnetic Resonance Centre Translational and Clinical Research Institute<br>Massarella, Clare; Newcastle University, Wellcome Centre for Mitochondrial Research, Translational and Clinical Research Institute,<br>Rehman, Rana; Newcastle University, Brain and Movement Research Group, Translational and Clinical Research Institute<br>Rochester, Lynn; Newcastle University, Brain and Movement Research Group, Translational and Clinical Research Institute,<br>Sayer, Avan; Newcastle Upon Tyne Hospitals NHS Foundation Trust, NIHR Biomedical Research Centre; Newcastle University, AGE Research Group, Translational and Clinical Research Institute<br>Su, Huizhong; Newcastle University, Brain and Movement Research Group, Translational and Clinical Research Institute,<br>Tuppen, Helen ; Newcastle University, Wellcome Centre for Mitochondrial Research, Translational and Clinical Research Institute<br>Warren, Charlotte; Newcastle University, Wellcome Centre for Mitochondrial Research, Translational and Clinical Research Institute<br>Witham, Miles; Newcastle University, NIHR Newcastle Biomedical Research Centre; Newcastle University, AGE Research Group, Translational and Clinical Research Institute

Keywords:	Clinical Trial, Aging, Neuromuscular disease < NEUROLOGY

Acipimox to improve muscle function and sarcopenia – Protocol for an open-label, uncontrolled, before-and-after experimental medicine feasibility study.

Authors

Claire McDonald[1,2,3]

Craig Alderson [4]

Matt G Birkbeck [4,5]

Laura Brown [6]

Silvia Del Din [2, 7]

Grainne Gorman [6]

Kieren G Hollingsworth [5]

Clare Massarella [6]

Rana Rehman [7]

Lynn Rochester [2, 4, 7]

Avan A Sayer [1, 2]

Huizhong Su [6]

Helen AL Tuppen [6]

Charlotte Warren [6]

Miles D Witham* [1, 2]

*Corresponding author

Affiliations

1. AGE Research Group, Translational and Clinical Research Institute, Faculty of Medical Sciences, Newcastle University, Newcastle, UK

For peer review only

2. NIHR Newcastle Biomedical Research Centre, Newcastle upon Tyne Hospitals NHS Foundation Trust, Cumbria, Northumberland, Tyne and Wear NHS Foundation Trust and Newcastle University
3. Gateshead Health NHS Foundation trust, Gateshead, UK.
4. Newcastle Upon Tyne Hospitals NHS Foundation Trust
5. Newcastle Magnetic Resonance Centre, Translational and Clinical Research Institute, Newcastle University
6. Wellcome Centre for Mitochondrial Research, Translational and Clinical Research Institute, Newcastle University
7. Brain and Movement Research Group, Translational and Clinical Research Institute, Newcastle University

Correspondence to:

Professor Miles D Witham, NIHR Newcastle Biomedical Research Centre, Newcastle University Campus for Ageing and Vitality, Newcastle upon Tyne, NE4 5PL, UK. Tel: +44 191 208 1317. Email: Miles.Witham@newcastle.ac.uk

For peer review only

Abstract

Introduction

Sarcopenia is the age associated loss of muscle mass and strength. Nicotinamide adenine dinucleotide (NAD) plays a central role in both mitochondrial function and cellular ageing processes implicated in sarcopenia. NAD concentrations are low in older people with sarcopenia, and increasing skeletal muscle NAD concentrations may offer a novel therapy for this condition. Acipimox is a licensed lipid-lowering agent known to act as an NAD precursor. This open-label, uncontrolled, before-and-after proof-of-concept experimental medicine study will test whether daily supplementation with acipimox improves skeletal muscle NAD concentrations.

Methods and analysis

Sixteen participants aged 65 and over with probable sarcopenia will receive acipimox 250 mg and aspirin 75mg orally daily for 4 weeks, with the frequency of acipimox administration being dependent on renal function. Muscle biopsy of the vastus lateralis and MRI scanning of the lower leg will be performed at baseline before starting acipimox and after 3 weeks of treatment. Adverse events will be recorded for the duration of the trial. The primary outcome, analysed in a per-protocol population, is the change in skeletal muscle NAD concentration between baseline and follow-up. Secondary outcomes include changes in phosphocreatine recovery rate by ^{31}P magnetic resonance spectroscopy, changes in physical performance and daily activity (hand grip strength, 4-metre walk and 7-day accelerometery), changes in skeletal muscle mitochondrial respiratory function, changes in skeletal muscle mitochondrial DNA copy number and changes in NAD concentrations in whole blood as a putative biomarker for future participant selection.

For peer review only

Ethics and dissemination

The trial is approved by the UK Medicines and Healthcare products Regulatory Agency (EuDRACT 2021-000993-28) UK Health Research Authority and Northeast – Tyne and Wear South Research Ethics Committee (IRAS 293565). Results will be made available to participants, their families, patients with sarcopenia, the public, regional and national clinical teams, and the international scientific community.

Strengths and limitations

1. This novel experimental medicine study is the first to test acipimox for older people living with sarcopenia and frailty.
2. It will ascertain whether it is feasible to use muscle biopsy-based trial outcome measures even in older people living with sarcopenia and frailty.
3. The trial design will provide a template for a suite of future experimental medicine studies in sarcopenia – a condition for which such studies are necessary but rarely done.
4. The trial will provide proof-of-concept mechanistic data, initial effect size data, and recruitment feasibility data to inform a future, larger randomised controlled trial.

Key words

Sarcopenia, Nicotinamide adenine dinucleotide, Acipimox, Clinical trial, Older people

Trial registration number

ISRCTN trial database (ISRCTN87404878)

Protocol

Acipimox feasibility study Clinical Trial Protocol V2 2/11/21

For peer review only

Introduction

Sarcopenia is the loss of muscle strength and mass that commonly accompanies ageing.[1] It is a syndrome of major clinical importance for many older people and affects between 5 and 10% of those aged over 65 in the general population, with rates in excess of 30% among those living in care homes.[2 3] Sarcopenia is associated with an increased risk of falls, hospitalisation, need for care and earlier death.[4-6] The cost of sarcopenia to the UK health service has been estimated to be in the region of 2 billion pounds per year.[7] At present, resistance exercise is the only intervention shown to be of benefit in sarcopenia.[3] However, this intervention is only partially effective at reversing the decline in muscle mass and strength, and not all older people are either willing or able to undertake resistance training. Alternative interventions are therefore required both to prevent and ameliorate sarcopenia and its deleterious consequences.

The pathophysiology of sarcopenia is complex, multifactorial, and incompletely understood. Impaired micro and macrovascular function, neuromuscular junction dysfunction, chronic inflammation and changes to the extracellular matrix have all been posited as being of importance.[1] Mitochondrial dysfunction, however, is postulated to play a central role in the aetiology of the condition. Mitochondrial numbers are low in sarcopenic muscle, and oxidative phosphorylation capacity is strikingly impaired, driven by reductions in expression and activity of all the major mitochondrial respiratory chain complexes.[8 9] Interventions that target deficits in mitochondrial function therefore provide an attractive mechanism to treat sarcopenia.

The role of NAD+ in muscle function

NAD+ and its reduced form (NADH) plays a central role in cellular redox homeostasis and is a cofactor in multiple pathways of cellular metabolism. NADH is essential for ATP production via oxidative phosphorylation in mitochondria, but it is also now clear that NAD+ is a key signalling molecule for

For peer review only - http://bmjopen.bmj.com/site/about/guidelines.xhtml

multiple pathways implicated in cellular ageing. [10] These include roles in protein deacetylation signalling via sirtuins, and DNA damage sensing (via poly-ADP-ribose polymerases; PARPs). These and other key NAD+ dependent systems such as the NADase CD38, which increases with ageing not only play important roles in cellular ageing, they also consume NAD+, leaving less for competing systems. NAD+ concentrations are lower at older age in multiple tissues, and lower concentrations are found in a wide range of degenerative diseases. [11] Recent data confirm that NAD+ concentrations are lower (by 32%) in muscle biopsies from older people with sarcopenia than in healthy, age-matched controls. [12] Therapies that increase NAD+ concentrations may therefore provide a way to reverse the changes commonly seen in ageing skeletal muscle which contribute to sarcopenia, including deficits in mitochondrial function. [10 11]

Acipimox is a nicotinic acid analogue and postulated to act as an NAD+ precursor. [13] Administration of acipimox may therefore be able to enhance skeletal muscle NAD concentrations in patients with sarcopenia, with consequent improvement in mitochondrial function and hence skeletal muscle function. Acipimox already has marketing authorisation for use as a lipid-lowering agent for patients with type IIb or type IV hyperlipidaemia who have not responded to other therapies. A recent systematic review identified 23 randomised controlled trials assessing the effect of NAD precursors on measures of physical function.[10] Studies were small (2-8 participants). A total of 96 primary outcomes were assessed, 10 of which were in favour of an NAD precursor, 1 was in favour of placebo; the remainder were not statistically significant in any clear direction. Small studies of acipimox that enrolled healthy younger patients or those with left ventricular dysfunction did not show improvement in measures of physical function.[14-18]

In contrast, muscle biopsy data from a trial of 21 patients with type 2 diabetes mellitus showed that 2 weeks of acipimox treatment (250mg twice a day) produced significant upregulation of mitochondrial genes involved in oxidative phosphorylation, an increase in skeletal muscle ATP content, and an

For peer review only

increase in ex-vivo mitochondrial respiration; no change in mitochondrial number measured by mtDNA copy number was noted.[13] The results of a placebo-controlled trial examining the impact of acipimox on mitochondrial function in patients with mitochondrial myopathy are awaited. [19]. However, none of these studies have assessed the presence of sarcopenia or frailty and enrolled younger or middle-aged participants.

Trial objectives

Trials to date have not tested acipimox or other NAD analogues in older people with sarcopenia or frailty. Testing whether acipimox has the expected biological effects in this population (which would be the target population for treatment in clinical practice) is a key step in ascertaining whether this therapy should be tested in larger randomised controlled trials for people with sarcopenia and frailty. The primary objective of this proof-of-concept experimental medicine study is therefore to test whether supplementation with acipimox improves skeletal muscle NAD concentrations. The secondary objectives are to test whether supplementation with acipimox improves mitochondrial respiratory chain function; to examine if individuals with low NAD concentrations in skeletal muscle biopsy can be identified by less invasive biomarkers (whole blood NAD concentration); and to assess candidate outcomes for future randomised clinical trials in patients with sarcopenia and frailty.

Methods and Analysis

Trial design and Setting

This is an open-label, uncontrolled, before-and-after feasibility study. Participants will be approached through older people's medicine clinics, day units and rehabilitation units. In addition, potential

For peer review only

participants will be identified through local primary care centres, acting as patient identification centres (PIC), and via a sarcopenia registry.[20] Screening visits will take place either in the participants' homes or in a clinical research facility. Study assessments will take place at the Clinical Ageing Research Unit and Clinical Research Facilities, based in Newcastle upon Tyne Hospitals NHS Foundation Trust.

Eligibility criteria

The target population are adults aged 65 years and over with sarcopenia. Sarcopenia is defined using hand grip strength or five times sit-to-stand thresholds from the 2019 European Working Group on Sarcopenia in Older People (EWGSOP) guidance.[21] In order to identify a frail / prefrail population, participants will also be required to have a walk speed <=0.8 m/s on a 4 metre walk test.[22] Participants must be able to give written informed consent. The study exclusion criteria are primarily focused on excluding participants for whom use of acipimox or aspirin may be unsafe or for whom MRI is contraindicated. Participants who are at risk of other types of skeletal muscle myopathy will be excluded to avoid confusion with the diagnosis of sarcopenia. The full inclusion and exclusion criteria for the study are shown in Table 1.

Interventions

All participants will take acipimox orally. Participants will be supplied with 250mg capsules, and the dose is adjusted according to renal function. Participants with creatinine clearance (estimated by the Cockcroft-Gault equation) >60 ml/min will be prescribed one capsule three times a day.[23] Participants with creatinine clearance 45-60 ml/min will be prescribed one capsule twice a day. Facial flushing is a common side effect of acipimox; this is likely to increase the risk of discontinuation of therapy and to cause unmasking of treatment allocation in any future randomised controlled trial. To suppress this side-effect, participants will be prescribed aspirin 75mg orally to be taken 30 minutes before the first dose of acipimox each day with the expectation that this intervention would also form part of a future randomised controlled trial as has been the case previously.[19] Participants who take aspirin as part of

For peer review only

their usual medication regime will continue their usual dose and will not be prescribed additional aspirin. If acipimox is discontinued aspirin will be discontinued.

Investigators may discontinue the trial treatment in the event of intolerable side effects occurring that are possibly, probably, or definitely related to trial medication or which constitute a serious adverse reaction (SAR) or suspected unexpected serious adverse reaction (SUSAR). Treatment will also be discontinued if the participant requests the medication to be withdrawn, requests not to undergo follow-up study measurements or if creatinine clearance falls below 45 ml/min. If creatinine clearance falls from above 60ml/min to between 45 and 60ml/min during the study, the dose frequency will be reduced from three times a day to twice a day. If acipimox is discontinued this will automatically lead to withdrawal from the trial.

Outcomes

Primary outcome

The primary outcome is change in skeletal muscle NAD+/NADH concentrations and ratio between baseline and follow up. To measure this outcome, muscle biopsy of vastus lateralis muscle will be performed at baseline and follow-up. Biopsies will be taken under local anaesthetic, using a Weil-Blakesley conchotome, a technique that has previously been shown to be well tolerated even by very old participants. [24-26] The second biopsy will be taken from the same leg as the first biopsy, a minimum of 3cm from the first biopsy site to avoid healing or inflammatory changes affecting the results of the second biopsy. Participants will be asked to withhold aspirin for 24 hours prior to biopsy. Aspirin (taken as part of their usual medication regime, or as adjunctive medication in the study to prevent flushing) can be started or restarted 48 hours after the biopsy. Biopsy samples will be weighed and snap frozen in liquid nitrogen cooled isopentane immediately after extraction, then stored at -80°C pending analysis. NAD+/NADH concentrations will be measured using a modified protocol of the Promega NAD/NADH-Glo assay kit (Promega, Southampton, UK).

For peer review only - http://bmjopen.bmj.com/site/about/guidelines.xhtml

Secondary outcomes

A range of measures of physical performance, mechanistic outcomes and outcomes related to trial performance will also be recorded at baseline and follow-up to assess the potential of these outcome for future randomised controlled trials.

Biopsy related outcomes

ATP/ADP concentrations and ratio will be assessed in the muscle biopsy samples according to Strehler[27] COX SDH histochemistry[28] and quadruple immunofluorescence[29] will be used to assess respiratory chain deficiency. Mitochondrial DNA copy number will be assessed via quantitative PCR[30]

Physical performance Measures

Maximal hand grip strength (measured using a Jamar Dynamometer) and the short physical performance battery will be performed at baseline and follow-up.[31 32]

Remote Digital Monitoring

Seven-day remote monitoring with a wearable device (AX6; Axivity Ltd, Newcastle upon Tyne, UK) will be used to measure indices of walking activity, gait speed, variability, and postural control at baseline and follow-up. [33 34 35]

Magnetic resonance imaging and Magnetic resonance spectroscopy outcomes

MR spectroscopy will assess phosphocreatine recovery rate measured by 31P MRS of the calf. The full MRI protocol has been published previously. [36] Changes in NAD(P)H levels by 31P MRS will also be assessed as an exploratory outcome. MRI of the calf muscle will be conducted at baseline and follow-up. Quantitative Dixon MRI will assess for intra-muscular fat infiltration.[37 38] An exploratory, diffusion weighted MRI technique - motor unit magnetic resonance imaging (MUMRI) - will be performed at rest to assess motor unit size via spontaneous muscle activity. [39]

For peer review only

Whole blood NAD concentration

Whole blood will be collected into EDTA tubes and frozen at -80C. Samples will be analysed for NAD+/NADH concentrations and ratio using Q-NADMED Blood NAD+ and NADH assay kit (NADMED, Helsinki, Finland) to test whether a precision target population (individuals with low skeletal muscle NAD+/NADH ratios at biopsy) can be identified by less invasive biomarkers.

Participation timeline

Recruitment and data management

Recruitment will take place over 12 months and started in October 2022. Potential participants are sent or given a brief study information sheet with an invitation letter, a reply slip and a prepaid envelope to express interest. The reply slip is returned to the study team and those who are interested will be contacted for pre-screening as described below. Data including numbers of potential participants identified, approached, and pre-screened will be collected and documented on the site screening log. Data will be collected on a paper case report forms and transposed to and secure Excel database. The participant trial record, including completed paper data collection tools, will be archived at site for 15 years following the end of the trial.

Pre-screening

Participants who express an interest in the trial will enter a brief telephone pre-screening process to check provisional eligibility. The pre-screening form is shown Supplementary material 1. Verbal consent will be taken for the pre-screening process including access to medical records. The SARC-F questionnaire will be administered; [40] this comprises five questions on physical function giving a total score between zero and ten. Based on previous observational data and trial experience, a score of one or more will be sufficient to identify those more likely to have sarcopenia and will enable progression to the screening visit.[41])[14] Participants who pass the pre-screen will be given, emailed or posted the

For peer review only

full participant information sheet (PIS), and all participants will be given at least 48 hours to consider their participation. The PIS can be found in Supplementary material 2. The study team will then telephone the potential participant, and those wishing to take part will then be invited to a screening visit.

Screening

Written informed consent will be obtained at the screening visit, which may take place at home, in clinic, or in a research facility. After informed consent is given, information on demographics, medical history, and medication history (including past history of allergy to acipimox or niacin related products) will be collected. Maximum handgrip, five time sit to stand and 4 metre walk speed will be measured. [22] Blood will be taken for full blood count (FBC), urea, creatinine, and electrolytes (U&Es) and liver function tests (LFTs – bilirubin, alanine aminotransferase and alkaline phosphatase). The vastus lateralis muscle will be inspected and palpated to ensure suitability for biopsy. Participants will be asked to confirm if they are taking part in any other interventional clinical trials or monitored exercise programs. Participant eligibility to proceed to the baseline visit is confirmed after the screening visit, once all screening assessment results are available.

Schedule of events

The schedule of events for the study is shown in Table 2. The primary and secondary outcomes are measured over two baseline visits and repeated at two follow-up visits.

Sample Size

As this is a feasibility study, few data exist on which to base a sample size calculation. The sample size has been selected to enable detection of a one standard deviation change in measures of mitochondrial NAD+/NADH ratios, ATP/ADP ratios or respiratory chain function. To detect this change

For peer review only

with an alpha of 0.05 and 80% power requires 11 paired observations (using a paired t-test). We plan to recruit 16 participants to allow for dropout and non-completion of the course of medication.

Statistical analysis

Analysis Population

The primary analysis population is the per-protocol population. This will consist of all participants who do not discontinue acipimox and undergo both baseline and follow up assessment for a given outcome. A safety analysis will be performed examining adverse events for all participants receiving at least one dose of acipimox.

Statistical Analysis Plan

A full statistical analysis plan (SAP) will be developed during the trial, which will include evaluation of the mechanistic outcomes data and correlation with main study outcomes. This will be developed by the Trial Management Group. Descriptive baseline statistics for all enrolled participants and for those completing the primary analysis (the per-protocol group) will be generated.

Analysis of the Primary Outcome Measure

The primary outcome is change in skeletal muscle NAD+/NADH ratios between baseline and follow up. Results will be analysed using a paired t-test (if normally distributed) or a Wilcoxon signed-rank test (if not normally distributed).

Analysis of Secondary Outcome Measures

Continuous secondary endpoints will be analysed in a similar manner to the primary outcome. Binary secondary endpoints will be analysed with a logistic regression model. Correlation between the changes in skeletal muscle NAD+/NADH ratios and changes in selected biomarkers (phosphocreatine recovery rate by MR spectroscopy, NAD+/NADH ratios in blood) will be performed using Pearson's

For peer review only

correlation coefficient (where measures are normally distributed) or Spearman's rho (where measures are not normally distributed)

Trial oversight

A Trial Management Group (TMG) will convene approximately monthly throughout the trial. Members will include the Chief Investigator, co-applicants, trial statistician, local site research staff and a Sponsor representative. The TMG undertakes the role of a Trial Steering Committee for this small, low risk CTIMP study. No independent data monitoring committee will be established given the low number of participants and the short duration of therapy. Instead, an interim safety review by the funder will examine the data from the first eight patients to complete the study.

Patient and Public Involvement

Patient consultation influenced the choice of outcomes and study design; previous feedback from patients has supported the use of both muscle biopsy and 31P MRS scanning as acceptable measures in this group of patients. Results from this feasibility study will be discussed with public and patient groups as part of the design process for a future randomised controlled trial.

Harms

All adverse events (AEs) occurring from point of consent to end of trial participation will be recorded. Falls will not be recorded as AEs, but any injury resulting from a fall will be recorded as an adverse event. Mild pain/soreness for the first 48 hours after muscle biopsy and superficial bruising at the site are to be expected and will not be recorded as AEs. Investigations, interventions, procedures, and operations will not be recorded as AEs, but the underlying condition (if new) leading to the intervention will be recorded as an AE. Complications resulting from investigations, interventions, procedures, and operations will be recorded as AEs.

For peer review only

Consent

Written informed consent will be obtained at screening visit before conducting any trial procedures including screening assessment. A medically qualified Investigator, competent in obtaining informed consent for research studies and formally delegated to do so by the site Principal Investigator, by way of dated signatures on the site delegation log, will conduct a consent interview with the participant at clinic or in the participants own home if preferred by the participant.

Ancillary and post-trial care

No provision for continuation of trial medication will be made by the trial team or Sponsor. Acipimox is not licensed for the indication under study in this trial; any off-licence use of acipimox after the end of the trial would be the responsibility of the participant's usual primary or secondary care clinical team.

Ethics and dissemination

Ethics approval

A favourable ethical opinion has been granted from the UK Health Research Authority and Northeast – Tyne and Wear South Research Ethics Committee (IRAS 293565). The trial has also received approval from the UK Medicines and Healthcare products Regulatory Agency (EuDRACT 2021-000993-28). The trial has been included in the National Institute for Health Research Clinical Research Network (NIHR CRN) portfolio (CPMS49429). The trial Sponsor is the Newcastle Upon Tyne Hospitals NHS Foundation Trust, Freeman Hospital, Freeman Road, High Heaton, Newcastle upon Tyne, NE7 7DN. The trial Sponsor has delegated responsibility for trial management to the CI , including trial design; review and approval of all localised patient-facing documentation prior to implementation at each site; collection, analysis and interpretation of data; writing of the protocol publication and final clinical report

For peer review only

manuscripts. This protocol manuscript is based on version 2.0 of the trial protocol (dated 2nd November 2021).

Dissemination policy

A final report of the trial will be provided to the Sponsor, Research Ethics Committee, and the trial Funder within one year of the end of the study. The trial results will be uploaded to the European Union Drug Regulating Authorities Clinical Trials (EudraCT) database, as per the European Commission's guidelines on posting and publication of result-related information within 12 months. The trial is registered on the ISRCTN trial database (ISRCTN87404878) and trial results will be made publicly available on the ISRCTN trial registry within 12 months of the end of the trial, defined as Last Patient Last Visit date. The final clinical trial report will be used for publication and presentation at scientific meetings. Summaries of results will also be made available to investigators for dissemination within their clinical areas and to the wider public, and a summary of results will be sent to all participants along with their treatment allocation. We will hold a dissemination event for participants and their families to present and discuss the study results.

Access to data

Access to the full dataset will be limited to the TMG and to authors of the trial publication. At the end of the trial, a de-identified dataset will be prepared and stored by Newcastle University. Requests for data sharing with study teams outside Newcastle University or the study Sponsor, including international requests, will be considered by a Data Access Committee with representation from the Funder, Sponsor and the trial Chief Investigator.

Discussion

Intervention studies for sarcopenia are challenging. Sarcopenia is often not diagnosed in clinical practice, and even when diagnosed, the diagnosis is often not recorded or coded in electronic patient

For peer review only

records, making participant identification difficult. Furthermore, patients with sarcopenia have, by definition, high rates of physical frailty; they also have high rates of multimorbidity. [42] These factors increase the risk that potential participants fail to meet inclusion and exclusion criteria or withdraw from clinical trials due to illness, death, adverse events or finding repeated trial interventions burdensome. This trial will build on our previous work to establish recruitment strategies that enable identification of patients with probable sarcopenia efficiently and at scale.[43] It will allow us to establish if our design for an experimental medicine sarcopenia trial is feasible to deliver for older people with sarcopenia and frailty, and whether acipimox is tolerated by older people with sarcopenia. This experimental medicine approach uses more intensive, more invasive procedures than have been used in many previous studies in sarcopenia. It will establish if our outcome measures (particularly multimodal MRI and repeated muscle biopsy) are acceptable to participants. Understanding how to design and deliver early phase experimental medicine trials that are acceptable older people with sarcopenia and frailty is essential to enable effective translation of preclinical knowledge of muscle biology into early-phase human interventional trials – a missing piece of the translational research circle for sarcopenia and frailty research.

Previous studies examining whether NAD precursors can increase NAD concentration in muscles have focused on younger populations without sarcopenia. This trial will provide pilot data to demonstrate if acipimox increases muscle NAD concentrations in older people with sarcopenia and frailty – the eventual target population for larger trials and for clinical practice. It will also suggest whether minimally invasive biomarkers can help guide which patients might benefit most from supplementation in future trials.

Facial flushing is a recognised side effect of acipimox, and we have chosen to co-administer aspirin to minimise this side effect and hence maximise trial retention and acipimox adherence. This co-administration does make interpretation of the results more difficult, as the anti-inflammatory or

For peer review only

haemodynamic effects of aspirin may influence muscle biochemistry and function independent of the effects of acipimox. Measurement of NAD-specific outcomes provides some mitigation to this challenge. Similarly, the lack of a control group in this trial limits our ability to ascribe changes in outcomes to the intervention alone, although the short time between baseline and follow up should reduce variability from non-intervention factors.

If acipimox does increase NAD concentrations in muscle and is associated with improvements in skeletal muscle biochemistry, energetics and function, double blinded randomised placebo-controlled trials will be needed to establish if acipimox can produce clinically meaningful improvements in physical performance, activities of daily living, and quality of life. This trial will gather pilot data on potential clinically important outcome measures both in the setting of the research unit and in the real-world setting by establishing the acceptability of 7-day activity monitoring to participants. The learning from this feasibility study, and from related intervention studies in the field of sarcopenia [ref], will also catalyse a new generation of experimental medicine studies for sarcopenia and frailty, necessary to bridge the gap between preclinical work and human trials. [44-46]

Authors contributions

Overall study conception and design: MDW, AAS, KGH, GG, LR

MRI design: MB, KGH

Laboratory analysis design and processes: LB, GG, CW, HALT, HS, CW

Accelerometry data collection and analysis design: SDD, RR, LR

Recruitment and medication use design: CA, CMcD

Manuscript preparation: CMcD

Critical revision of manuscript: All authors

For peer review only

Funding

This work was funded by the UKRI Medical Research Council Confidence in Concept funding scheme (grant number MC_PC_19047)

Competing interests

S. Del Din reports consultancy activity with Hoffmann-La Roche Ltd. outside of this study.

Acknowledgements

CMcD, MDW, AAS, GG, SDD and LR acknowledge support from the National Institute for Health and Care Research (NIHR) Newcastle Biomedical Research Centre (BRC). The NIHR Newcastle BRC is a partnership between Newcastle Hospitals NHS Foundation Trust, Cumbria Northumberland Tyne and Wear NHS Foundation Trust, and Newcastle University. The views expressed are those of the authors and not necessarily those of the NIHR or the Department of Health and Social Care.

SDD and LR were also supported by the Mobilise-D project that has received funding from the Innovative Medicines Initiative 2 Joint Undertaking (JU) under grant agreement No. 820820. This JU receives support from the European Union's Horizon 2020 research and innovation program and the European Federation of Pharmaceutical Industries and Associations (EFPIA). SDD and LR were also supported by the Innovative Medicines Initiative 2 Joint Undertaking (IMI2 JU) project IDEA-FAST - Grant Agreement 853981. SDD and LR were supported by the NIHR/Wellcome Trust Clinical Research Facility (CRF) infrastructure at Newcastle upon Tyne Hospitals NHS Foundation Trust.

We acknowledge the contribution of our much-missed colleague Dr Richard Dodds, who sadly died before this work could be completed.

For peer review only

Tables

Table 1 Inclusion and Exclusion Criteria

INCLUSION CRITERIA
• Age 65 years or over<br>• Low maximum handgrip strength (<16kg for women, <27kg for men) OR five-times sit to stand time >15 seconds (inability to complete sit to stand test will count as time >15seconds)<br>• Walk speed <=0.8 m/s on 4 metre walk test
**EXCLUSION CRITERIA**
General:<br>• Allergy to acipimox or other niacin-related products<br>• Allergy or intolerance of aspirin<br>• Any contraindication to taking aspirin<br>• Unable to give written informed consent<br>• Currently enrolled in another intervention study (observational studies are permitted)<br>• Currently participating in supervised exercise classes or physiotherapy<br>• Any progressive neurological or malignant condition with life expectancy <6 months<br>Safety of investigations medications:<br>• Creatinine Clearance <45ml/min (by Cockcroft-Gault equation)<br>• Taking statin medication or fibrate medication<br>• Active peptic ulcer disease or dyspepsia<br>Safety of muscle biopsy and MRI:<br>• Platelets <100x10^9/L at screening (contraindication to muscle biopsy)<br>• Presence of a bleeding diathesis or use of oral or parenteral anticoagulant medication<br>• Antiplatelet agents other than low dose (75mg once daily) aspirin<br>• Contraindications to MRI scanning (mild claustrophobia is not a contraindication)<br>• Allergy to local anaesthetic (lidocaine)<br>• Unable to palpate vastus lateralis muscle to enable biopsy localisation<br>Other causes of skeletal myopathy:<br>• Liver function tests (bilirubin, ALT, alkaline phosphatase) > 3x ULN<br>• Symptomatic (NYHA class II-IV) chronic heart failure (diagnosed according to European Society of Cardiology guidelines)<br>• Severe COPD (GOLD stage IV)<br>• Known myositis or other established myopathy<br>• Self-reported weight loss of >10% in last 6 months (to exclude significant cachexia)<br>• Known uncontrolled thyrotoxicosis<br>• 7.5mg/day or greater prednisolone use (or equivalent)

For peer review only

Table 2 Schedule of events

	**Visit 1**	**Visit 2**	**Visit 3**	**Visit 4***	**Visit 5**	**Visit 6**	**Visit 7**	**Visit 8**	**Visit 9**	**Visit 10**
	**Pre-Screen**	**Screening**	**Baseline 1**	**Baseline 2**	**Phone call**	**Phone call**	**Follow up 1**	**Follow up 2**	**Phone call**	**Phone call**
**Assessment**		**Day -28 to -1**	**Day 0**	**Day 7 (+/- 1 day)**	**Day 9 (+/- 1 day)**	**Day 14 (+/- 2 days)**	**Day 21 (+/- 3 days)**	**Day 28 (+/- 3 days)**	**Day 30 (+/- 3 days)**	**Day 37 (+/- 4 days)**
SARC-F questions	X									
Demographics	X	X								
Medical history	X	X								
Concomitant medications	X	X	X	X			X	X		
Informed written consent		X								
Height		X								
Weight		X								
Bloods (U+Es, liver function tests)		X								
Bloods (Full blood count)		X								
Bloods (research bloods for NAD levels)			X				X			
Frailty questions (activity, exhaustion, weight loss)			X				X			
Muscle biopsy				X				X		
MRI scanning			X				X			

For peer review only

Accelerometer given out			X				X			
Accelerometer returned				X				X		
Medication dispensing				X						
Instructions on taking medication					X				X	
Medication adherence (collection/count)								X		
Telephone check of wound healing					X	X			X	X
Adverse Events log completion		X	X	X	X	X	X	X	X	X
4m walk speed		X								
5-times sit to stand		X								
Grip strength		X	X				X			
Short Physical Performance Battery			X				X			
Eligibility confirmation		X								

For peer review only

References

1. Cruz-Jentoft AJ, Sayer AA. Sarcopenia. *Lancet* 2019;393(10191):2636-46. doi: 10.1016/S0140-6736(19)31138-9
2. Patel HP, Syddall HE, Jameson K, et al. Prevalence of sarcopenia in community-dwelling older people in the UK using the European Working Group on Sarcopenia in Older People (EWGSOP) definition: findings from the Hertfordshire Cohort Study (HCS). *Age and ageing* 2013;42(3):378-84.
3. Cruz-Jentoft AJ, Landi F, Schneider SM, et al. Prevalence of and interventions for sarcopenia in ageing adults: a systematic review. Report of the International Sarcopenia Initiative (EWGSOP and IWGS). *Age and ageing* 2014;43(6):748-59.
4. Lunt E, Ong T, Gordon AL, et al. The clinical usefulness of muscle mass and strength measures in older people: a systematic review. *Age and Ageing* 2020;50(1):88-95. doi: 10.1093/ageing/afaa123
5. Liu P, Hao Q, Hai S, et al. Sarcopenia as a predictor of all-cause mortality among community-dwelling older people: a systematic review and meta-analysis. *Maturitas* 2017;103:16-22.
6. Yeung SS, Reijnierse EM, Pham VK, et al. Sarcopenia and its association with falls and fractures in older adults: a systematic review and meta-analysis. *Journal of cachexia, sarcopenia and muscle* 2019;10(3):485-500.
7. Pinedo-Villanueva R, Westbury LD, Syddall HE, et al. Health care costs associated with muscle weakness: a UK population-based estimate. *Calcified tissue international* 2019;104:137-44.
8. Bellanti F, Lo Buglio A, Vendemiale G. Mitochondrial impairment in sarcopenia. *Biology* 2021;10(1):31.
9. Boffoli D, Scacco S, Vergari R, et al. Decline with age of the respiratory chain activity in human skeletal muscle. *Biochimica et Biophysica Acta (BBA)-Molecular Basis of Disease* 1994;1226(1):73-82.
10. Barker FJ, Hart A, Sayer AA, et al. Effects of nicotinamide adenine dinucleotide precursors on measures of physical performance and physical frailty: A systematic review. *JCSM Clinical Reports* 2022;7(4):93-106.
11. Camacho-Pereira J, Tarragó MG, Chini CC, et al. CD38 dictates age-related NAD decline and mitochondrial dysfunction through an SIRT3-dependent mechanism. *Cell metabolism* 2016;23(6):1127-39.
12. Migliavacca E, Tay SKH, Patel HP, et al. Mitochondrial oxidative capacity and NAD(+) biosynthesis are reduced in human sarcopenia across ethnicities. *Nat Commun* 2019;10(1):5808. doi: 10.1038/s41467-019-13694-1 [published Online First: 20191220]
13. Van De Weijer T, Phielix E, Bilet L, et al. Evidence for a direct effect of the NAD+ precursor acipimox on muscle mitochondrial function in humans. *Diabetes* 2015;64(4):1193-201.
14. Gautier J, Pirnay F, Jandrain B, et al. Endogenous substrate oxidation during exercise and variations in breath 13CO2/12CO2. *Journal of Applied Physiology* 1993;74(1):133-38.
15. Head A, Jakeman P, Kendall M, et al. The impact of a short course of three lipid lowering drugs on fat oxidation during exercise in healthy volunteers. *Postgraduate medical journal* 1993;69(809):197-203.
16. Nellemann B, Søndergaard E, Jensen J, et al. Kinetics and utilization of lipid sources during acute exercise and acipimox. *American Journal of Physiology-Endocrinology and Metabolism* 2014;307(2):E199-E208.
17. Halbirk M, Nørrelund H, Møller N, et al. Suppression of circulating free fatty acids with acipimox in chronic heart failure patients changes whole body metabolism but does not affect cardiac

For peer review only - http://bmjopen.bmj.com/site/about/guidelines.xhtml

function. *American Journal of Physiology-Heart and Circulatory Physiology* 2010;299(4):H1220-H25.
18. Makimura H, Stanley TL, Suresh C, et al. Metabolic effects of long-term reduction in free fatty acids with acipimox in obesity: a randomized trial. *The Journal of Clinical Endocrinology & Metabolism* 2016;101(3):1123-33.
19. Abouhajar A, Alcock L, Bigirumurame T, et al. Acipimox in Mitochondrial Myopathy (AIMM): study protocol for a randomised, double-blinded, placebo-controlled, adaptive design trial of the efficacy of acipimox in adult patients with mitochondrial myopathy. *Trials* 2022;23(1):1-15.
20. Witham MD, Heslop P, Dodds RM, et al. Developing a UK sarcopenia registry: recruitment and baseline characteristics of the SarcNet pilot. *Age and Ageing* 2021;50(5):1762-69.
21. Cruz-Jentoft AJ, Bahat G, Bauer J, et al. Sarcopenia: revised European consensus on definition and diagnosis. *Age and ageing* 2019;48(1):16-31.
22. Abellan Van Kan G, Rolland Y, Andrieu S, et al. Gait speed at usual pace as a predictor of adverse outcomes in community-dwelling older people an International Academy on Nutrition and Aging (IANA) Task Force. *The journal of nutrition, health & aging* 2009;13(10):881-89. doi: 10.1007/s12603-009-0246-z
23. Cockcroft DW, Gault MH. Prediction of creatinine clearance from serum creatinine. *Nephron* 1976;16(1):31-41. doi: 10.1159/000180580
24. Patel H, Syddall HE, Martin HJ, et al. In epidemiological studies: Findings from the hertfordshire sarcopenia study (HSS). *The journal of nutrition, health & aging* 2011;15(1):10-15. doi: 10.1007/s12603-011-0006-8
25. Dodds R, Hurst C, Hillman S, et al. Advancing our understanding of skeletal muscle across the lifecourse: Protocol for the MASS_Lifecourse study and characteristics of the first 80 participants. *Experimental Gerontology* 2022;166:111884.
26. Dodds R, Davies K, Granic A, et al. Mitochondrial respiratory chain function and content are preserved in the skeletal muscle of active very old men and women. *Experimental Gerontology* 2018;113:80-85.
27. Strehler BL. Adenosine-5'-triphosphate and creatine phosphate determination with luciferase. Methods of enzymatic analysis: Elsevier 1974:2112-26.
28. Old SL, Johnson MA. Methods of microphotometric assay of succinate dehydrogenase and cytochrome c oxidase activities for use on human skeletal muscle. *The Histochemical journal* 1989;21:545-55.
29. Rocha MC, Grady JP, Grünewald A, et al. A novel immunofluorescent assay to investigate oxidative phosphorylation deficiency in mitochondrial myopathy: understanding mechanisms and improving diagnosis. *Scientific reports* 2015;5(1):15037.
30. Grady JP, Murphy JL, Blakely EL, et al. Accurate measurement of mitochondrial DNA deletion level and copy number differences in human skeletal muscle. *PLoS One* 2014;9(12):e114462.
31. Roberts HC, Denison HJ, Martin HJ, et al. A review of the measurement of grip strength in clinical and epidemiological studies: towards a standardised approach. *Age and ageing* 2011;40(4):423-29.
32. Guralnik JM, Simonsick EM, Ferrucci L, et al. A short physical performance battery assessing lower extremity function: association with self-reported disability and prediction of mortality and nursing home admission. *Journal of gerontology* 1994;49(2):M85-M94.
33. Axivity. AX6 Datasheet 6-Axis logging movement sensor 2023 [Available from: https://axivity.com/files/resources/AX6_Data_Sheet.pdf.
34. Del Din S, Galna B, Godfrey A, et al. Analysis of free-living gait in older adults with and without Parkinson's disease and with and without a history of falls: identifying generic and disease-specific characteristics. *The Journals of Gerontology: Series A* 2019;74(4):500-06.
35. Del Din S, Galna B, Lord S, et al. Falls risk in relation to activity exposure in high-risk older adults. *The Journals of Gerontology: Series A* 2020;75(6):1198-205.

For peer review only

36. Hollingsworth KG, Newton JL, Taylor R, et al. Pilot study of peripheral muscle function in primary biliary cirrhosis: potential implications for fatigue pathogenesis. *Clin Gastroenterol Hepatol* 2008;6(9):1041-8. doi: 10.1016/j.cgh.2008.04.013 [published Online First: 20080808]
37. Loughran T, Higgins DM, McCallum M, et al. Improving highly accelerated fat fraction measurements for clinical trials in muscular dystrophy: origin and quantitative effect of R2* changes. *Radiology* 2015;275(2):570-78.
38. Burakiewicz J, Sinclair CDJ, Fischer D, et al. Quantifying fat replacement of muscle by quantitative MRI in muscular dystrophy. *J Neurol* 2017;264(10):2053-67. doi: 10.1007/s00415-017-8547-3 [published Online First: 20170701]
39. Birkbeck MG, Blamire AM, Whittaker RG, et al. The role of novel motor unit magnetic resonance imaging to investigate motor unit activity in ageing skeletal muscle. *Journal of cachexia, sarcopenia and muscle* 2021;12(1):17-29.
40. Malmstrom TK, Morley JE. SARC-F: a simple questionnaire to rapidly diagnose sarcopenia. *Journal of the American Medical Directors Association* 2013;14(8):531-32.
41. Dodds RM, Murray JC, Robinson SM, et al. The identification of probable sarcopenia in early old age based on the SARC-F tool and clinical suspicion: findings from the 1946 British birth cohort. *Eur Geriatr Med* 2020;11(3):433-41. doi: 10.1007/s41999-020-00310-5 [published Online First: 20200319]
42. Dodds RM, Granic A, Robinson SM, et al. Sarcopenia, long-term conditions, and multimorbidity: findings from UK Biobank participants. *J Cachexia Sarcopenia Muscle* 2020;11(1):62-68. doi: 10.1002/jcsm.12503 [published Online First: 20191230]
43. Witham MD, Achison M, Aspray TJ, et al. Recruitment strategies for sarcopenia trials: lessons from the LACE randomized controlled trial. *JCSM Rapid Communications* 2021;4(2):93-102. doi: https://doi.org/10.1002/rco2.38
44. Witham MD, Cooper R, Bowden Davies KA, et al. Ageing research translation: a new era for UK Geroscience. *Nature Aging* 2022;2(10):867-68.
45. Witham MD. Bridging the gap between the laboratory and the clinic for patients with sarcopenia. *Biogerontology* 2019;20(2):241-48.
46. Bennett JL, Egail M, Anderson AE, et al. The Rheumatoid Arthritis and MUScle (RAMUS) Study: Protocol for an observational single-arm study of skeletal muscle in patients with rheumatoid arthritis receiving tofacitinib. *J Frailty Sarcopenia Falls* 2023;8(1):53-59. doi: 10.22540/jfsf-08-053 [published Online First: 20230301]

For peer review only

Reporting checklist for protocol of a clinical trial.

Based on the SPIRIT guidelines.

Instructions to authors

Complete this checklist by entering the page numbers from your manuscript where readers will find each of the items listed below.

Your article may not currently address all the items on the checklist. Please modify your text to include the missing information. If you are certain that an item does not apply, please write "n/a" and provide a short explanation.

Upload your completed checklist as an extra file when you submit to a journal.

In your methods section, say that you used the SPIRITreporting guidelines, and cite them as:

Chan A-W, Tetzlaff JM, Gøtzsche PC, Altman DG, Mann H, Berlin J, Dickersin K, Hróbjartsson A, Schulz KF, Parulekar WR, Krleža-Jerić K, Laupacis A, Moher D. SPIRIT 2013 Explanation and Elaboration: Guidance for protocols of clinical trials. BMJ. 2013;346:e7586

		Reporting Item	Page Number
**Administrative information**			
Title	#1	Descriptive title identifying the study design, population, interventions, and, if applicable, trial acronym	1

For peer review only

Trial registration	#2a	Trial identifier and registry name. If not yet registered, name of intended registry	5
Trial registration: data set	#2b	All items from the World Health Organization Trial Registration Data Set	
Protocol version	#3	Date and version identifier	4 & 17
Funding	#4	Sources and types of financial, material, and other support	19
Roles and responsibilities: contributorship	#5a	Names, affiliations, and roles of protocol contributors	1-2, 19
Roles and responsibilities: sponsor contact information	#5b	Name and contact information for the trial sponsor	16
Roles and responsibilities: sponsor and funder	#5c	Role of study sponsor and funders, if any, in study design; collection, management, analysis, and interpretation of data; writing of the report; and the decision to submit the report for publication, including whether they will have ultimate authority over any of these activities	NA
Roles and responsibilities: committees	#5d	Composition, roles, and responsibilities of the coordinating centre, steering committee, endpoint adjudication committee, data management team, and	15

For peer review only

		other individuals or groups overseeing the trial, if applicable (see Item 21a for data monitoring committee)	
**Introduction**			
Background and rationale	#6a	Description of research question and justification for undertaking the trial, including summary of relevant studies (published and unpublished) examining benefits and harms for each intervention	6
Background and rationale: choice of comparators	#6b	Explanation for choice of comparators	NA
Objectives	#7	Specific objectives or hypotheses	8
Trial design	#8	Description of trial design including type of trial (eg, parallel group, crossover, factorial, single group), allocation ratio, and framework (eg, superiority, equivalence, non-inferiority, exploratory)	8
**Methods: Participants, interventions, and outcomes**			
Study setting	#9	Description of study settings (eg, community clinic, academic hospital) and list of countries where data will	8

For peer review only

		be collected. Reference to where list of study sites can be obtained	
Eligibility criteria	#10	Inclusion and exclusion criteria for participants. If applicable, eligibility criteria for study centres and individuals who will perform the interventions (eg, surgeons, psychotherapists)	9
Interventions: description	#11a	Interventions for each group with sufficient detail to allow replication, including how and when they will be administered	9
Interventions: modifications	#11b	Criteria for discontinuing or modifying allocated interventions for a given trial participant (eg, drug dose change in response to harms, participant request, or improving / worsening disease)	9
Interventions: adherance	#11c	Strategies to improve adherence to intervention protocols, and any procedures for monitoring adherence (eg, drug tablet return; laboratory tests)	22
Interventions: concomitant care	#11d	Relevant concomitant care and interventions that are permitted or prohibited during the trial	8&9
Outcomes	#12	Primary, secondary, and other outcomes, including the specific measurement variable (eg, systolic blood pressure), analysis metric (eg, change from baseline, final value, time to event), method of aggregation (eg, median, proportion), and time point for each outcome.	10-13

For peer review only

		Explanation of the clinical relevance of chosen efficacy and harm outcomes is strongly recommended	
Participant timeline	#13	Time schedule of enrolment, interventions (including any run-ins and washouts), assessments, and visits for participants. A schematic diagram is highly recommended (see Figure)	12-11
Sample size	#14	Estimated number of participants needed to achieve study objectives and how it was determined, including clinical and statistical assumptions supporting any sample size calculations	13-14
Recruitment	#15	Strategies for achieving adequate participant enrolment to reach target sample size	8
**Methods: Assignment of interventions (for controlled trials)**			
Allocation: sequence generation	#16a	Method of generating the allocation sequence (eg, computer-generated random numbers), and list of any factors for stratification. To reduce predictability of a random sequence, details of any planned restriction (eg, blocking) should be provided in a separate document that is unavailable to those who enrol participants or assign interventions	NA

For peer review only

Allocation concealment mechanism	#16b	Mechanism of implementing the allocation sequence (eg, central telephone; sequentially numbered, opaque, sealed envelopes), describing any steps to conceal the sequence until interventions are assigned	NA
Allocation: implementation	#16c	Who will generate the allocation sequence, who will enrol participants, and who will assign participants to interventions	NA
Blinding (masking)	#17a	Who will be blinded after assignment to interventions (eg, trial participants, care providers, outcome assessors, data analysts), and how	NA
Blinding (masking): emergency unblinding	#17b	If blinded, circumstances under which unblinding is permissible, and procedure for revealing a participant's allocated intervention during the trial	NA
**Methods: Data collection, management, and analysis**			
Data collection plan	#18a	Plans for assessment and collection of outcome, baseline, and other trial data, including any related processes to promote data quality (eg, duplicate measurements, training of assessors) and a description of study instruments (eg, questionnaires, laboratory tests) along with their reliability and validity,	12

For peer review only

		if known. Reference to where data collection forms can be found, if not in the protocol	
Data collection plan: retention	#18b	Plans to promote participant retention and complete follow-up, including list of any outcome data to be collected for participants who discontinue or deviate from intervention protocols	12
Data management	#19	Plans for data entry, coding, security, and storage, including any related processes to promote data quality (eg, double data entry; range checks for data values). Reference to where details of data management procedures can be found, if not in the protocol	12
Statistics: outcomes	#20a	Statistical methods for analysing primary and secondary outcomes. Reference to where other details of the statistical analysis plan can be found, if not in the protocol	14
Statistics: additional analyses	#20b	Methods for any additional analyses (eg, subgroup and adjusted analyses)	14
Statistics: analysis population and missing data	#20c	Definition of analysis population relating to protocol non-adherence (eg, as randomised analysis), and any statistical methods to handle missing data (eg, multiple imputation)	14
**Methods: Monitoring**			

For peer review only

Data monitoring: formal committee	#21a	Composition of data monitoring committee (DMC); summary of its role and reporting structure; statement of whether it is independent from the sponsor and competing interests; and reference to where further details about its charter can be found, if not in the protocol. Alternatively, an explanation of why a DMC is not needed	15
Data monitoring: interim analysis	#21b	Description of any interim analyses and stopping guidelines, including who will have access to these interim results and make the final decision to terminate the trial	15
Harms	#22	Plans for collecting, assessing, reporting, and managing solicited and spontaneously reported adverse events and other unintended effects of trial interventions or trial conduct	15
Auditing	#23	Frequency and procedures for auditing trial conduct, if any, and whether the process will be independent from investigators and the sponsor	15
**Ethics and dissemination**			
Research ethics approval	#24	Plans for seeking research ethics committee / institutional review board (REC / IRB) approval	16

For peer review only

Protocol amendments	#25	Plans for communicating important protocol modifications (eg, changes to eligibility criteria, outcomes, analyses) to relevant parties (eg, investigators, REC / IRBs, trial participants, trial registries, journals, regulators)	15
Consent or assent	#26a	Who will obtain informed consent or assent from potential trial participants or authorised surrogates, and how (see Item 32)	16
Consent or assent: ancillary studies	#26b	Additional consent provisions for collection and use of participant data and biological specimens in ancillary studies, if applicable	NA
Confidentiality	#27	How personal information about potential and enrolled participants will be collected, shared, and maintained in order to protect confidentiality before, during, and after the trial	11, 18 and ICF
Declaration of interests	#28	Financial and other competing interests for principal investigators for the overall trial and each study site	20
Data access	#29	Statement of who will have access to the final trial dataset, and disclosure of contractual agreements that limit such access for investigators	17
Ancillary and post trial care	#30	Provisions, if any, for ancillary and post-trial care, and for compensation to those who suffer harm from trial participation	16

For peer review only

Dissemination policy: trial results	#31a	Plans for investigators and sponsor to communicate trial results to participants, healthcare professionals, the public, and other relevant groups (eg, via publication, reporting in results databases, or other data sharing arrangements), including any publication restrictions	17
Dissemination policy: authorship	#31b	Authorship eligibility guidelines and any intended use of professional writers	16
Dissemination policy: reproducible research	#31c	Plans, if any, for granting public access to the full protocol, participant-level dataset, and statistical code	NA
**Appendices**			
Informed consent materials	#32	Model consent form and other related documentation given to participants and authorised surrogates	Separate attachment
Biological specimens	#33	Plans for collection, laboratory evaluation, and storage of biological specimens for genetic or molecular analysis in the current trial and for future use in ancillary studies, if applicable	9/10/11

None The SPIRIT Explanation and Elaboration paper is distributed under the terms of the Creative Commons Attribution License CC-BY-NC. This checklist can be completed online using https://www.goodreports.org/, a tool made by the EQUATOR Network in collaboration with Penelope.ai

For peer review only

BMJ Open

A study protocol to investigate if Acipimox improves muscle function and sarcopenia –an open-label, uncontrolled, before-and-after experimental medicine feasibility study.

Journal:	*BMJ Open*
Manuscript ID	bmjopen-2023-076518.R1
Article Type:	Protocol
Date Submitted by the Author:	19-Dec-2023

Complete List of Authors:	McDonald, Claire; Newcastle University, AGE Research Group, Translational and Clinical Research Institute, Faculty of Medical Sciences; Newcastle Upon Tyne Hospitals NHS Foundation Trust, NIHR Newcastle Biomedical Research Centre<br>Alderson , Craig; Newcastle Upon Tyne Hospitals NHS Foundation Trust<br>Birkbeck, Matthew; Newcastle University, Newcastle Magnetic Resonance Centre Translational and Clinical Research Institute, ; Newcastle Upon Tyne Hospitals NHS Foundation Trust<br>Brown, Laura ; Newcastle University, Wellcome Centre for Mitochondrial Research, Translational and Clinical Research Institute<br>Del Din, Silvia; Newcastle University, Brain and Movement Research Group, Translational and Clinical Research Institute,<br>Gorman, Grainne; Newcastle University, Wellcome Centre for Mitochondrial Research, Translational and Clinical Research Institute<br>Hollingsworth, Kieren; Newcastle University, Newcastle Magnetic Resonance Centre Translational and Clinical Research Institute<br>Massarella, Clare; Newcastle University, Wellcome Centre for Mitochondrial Research, Translational and Clinical Research Institute,<br>Rehman, Rana; Newcastle University, Brain and Movement Research Group, Translational and Clinical Research Institute<br>Rochester, Lynn; Newcastle University, Brain and Movement Research Group, Translational and Clinical Research Institute,<br>Sayer, Avan; Newcastle Upon Tyne Hospitals NHS Foundation Trust, NIHR Biomedical Research Centre; Newcastle University, AGE Research Group, Translational and Clinical Research Institute<br>Su, Huizhong; Newcastle University, Brain and Movement Research Group, Translational and Clinical Research Institute,<br>Tuppen, Helen ; Newcastle University, Wellcome Centre for Mitochondrial Research, Translational and Clinical Research Institute<br>Warren, Charlotte; Newcastle University, Wellcome Centre for Mitochondrial Research, Translational and Clinical Research Institute<br>Witham, Miles; Newcastle University, NIHR Newcastle Biomedical Research Centre; Newcastle University, AGE Research Group, Translational and Clinical Research Institute

**Primary Subject Heading**:	Geriatric medicine

Secondary Subject Heading:	Evidence based practice
Keywords:	Clinical Trial, Aging, Neuromuscular disease < NEUROLOGY

SCHOLARONE™ Manuscripts

A study protocol to investigate if Acipimox improves muscle function and sarcopenia –an open-label, uncontrolled, before-and-after experimental medicine feasibility study.

Authors

Claire McDonald[1,2,3]

Craig Alderson [4]

Matt G Birkbeck [4,5]

Laura Brown [6]

Silvia Del Din [2, 7]

Grainne Gorman [6]

Kieren G Hollingsworth [5]

Clare Massarella [6]

Rana Rehman [7]

Lynn Rochester [2, 4, 7]

Avan A Sayer [1, 2]

Huizhong Su [6]

Helen AL Tuppen [6]

Charlotte Warren [6]

Miles D Witham* [1, 2]

*Corresponding author

Affiliations

1. AGE Research Group, Translational and Clinical Research Institute, Faculty of Medical Sciences, Newcastle University, Newcastle, UK

For peer review only

2. NIHR Newcastle Biomedical Research Centre, Newcastle upon Tyne Hospitals NHS Foundation Trust, Cumbria, Northumberland, Tyne and Wear NHS Foundation Trust and Newcastle University
3. Gateshead Health NHS Foundation trust, Gateshead, UK.
4. Newcastle Upon Tyne Hospitals NHS Foundation Trust
5. Newcastle Magnetic Resonance Centre, Translational and Clinical Research Institute, Newcastle University
6. Wellcome Centre for Mitochondrial Research, Translational and Clinical Research Institute, Newcastle University
7. Brain and Movement Research Group, Translational and Clinical Research Institute, Newcastle University

Correspondence to:

Professor Miles D Witham, NIHR Newcastle Biomedical Research Centre, Newcastle University Campus for Ageing and Vitality, Newcastle upon Tyne, NE4 5PL, UK. Tel: +44 191 208 1317. Email: Miles.Witham@newcastle.ac.uk

For peer review only

Abstract

Introduction

Sarcopenia is the age associated loss of muscle mass and strength. Nicotinamide adenine dinucleotide (NAD) plays a central role in both mitochondrial function and cellular ageing processes implicated in sarcopenia. NAD concentrations are low in older people with sarcopenia, and increasing skeletal muscle NAD concentrations may offer a novel therapy for this condition. Acipimox is a licensed lipid-lowering agent known to act as an NAD precursor. This open-label, uncontrolled, before-and-after proof-of-concept experimental medicine study will test whether daily supplementation with acipimox improves skeletal muscle NAD concentrations.

Methods and analysis

Sixteen participants aged 65 and over with probable sarcopenia will receive acipimox 250 mg and aspirin 75mg orally daily for 4 weeks, with the frequency of acipimox administration being dependent on renal function. Muscle biopsy of the vastus lateralis and MRI scanning of the lower leg will be performed at baseline before starting acipimox and after 3 weeks of treatment. Adverse events will be recorded for the duration of the trial. The primary outcome, analysed in a per-protocol population, is the change in skeletal muscle NAD concentration between baseline and follow-up. Secondary outcomes include changes in phosphocreatine recovery rate by ^{31}P magnetic resonance spectroscopy, changes in physical performance and daily activity (hand grip strength, 4-metre walk and 7-day accelerometery), changes in skeletal muscle mitochondrial respiratory function, changes in skeletal muscle mitochondrial DNA copy number and changes in NAD concentrations in whole blood as a putative biomarker for future participant selection.

For peer review only

Ethics and dissemination

The trial is approved by the UK Medicines and Healthcare products Regulatory Agency (EuDRACT 2021-000993-28) UK Health Research Authority and Northeast – Tyne and Wear South Research Ethics Committee (IRAS 293565). Results will be made available to participants, their families, patients with sarcopenia, the public, regional and national clinical teams, and the international scientific community.

Strengths and limitations

1. This study will recruit older people with sarcopenia – a population traditionally hard to include in trials and with whom experimental medicine studies are not commonly performed.
2. It will use muscle biopsy-based outcome measures, and will demonstrate feasibility of doing serial muscle biopsies in this population.
3. A range of other, minimally-invasive exploratory outcome measures (motor unit magnetic resonance imaging (MUMRI), whole blood NAD+/NADH and digital monitoring) will be measured to assess their value in sarcopenia clinical trials .
4. The study is not randomised, and larger randomised controlled trials will be required to assess efficacy if results from this proof-of-concept trial are promising.
5. Co-administration of aspirin with acipimox should improve tolerability of treatment and reflects the way that treatment would be used in clinical practice but does complicate efforts to understand the mechanism behind any beneficial effect on skeletal muscle function.

Key words

Sarcopenia, Nicotinamide adenine dinucleotide, Acipimox, Clinical trial, Older people

Trial registration number

ISRCTN trial database (ISRCTN87404878)

For peer review only

Protocol

Acipimox feasibility study Clinical Trial Protocol V2 2/11/21

For peer review only

Introduction

Sarcopenia is the loss of muscle strength and mass that commonly accompanies ageing. It is a syndrome of major clinical importance for many older people and affects between 5 and 10% of those aged over 65 in the general population, with rates in excess of 30% among those living in care homes.[1 2] Sarcopenia is associated with an increased risk of falls, hospitalisation, need for care and earlier death.[3-5] The cost of sarcopenia to the UK health service has been estimated to be in the region of 2 billion pounds per year.[6]

In recent decades several definitions of sarcopenia have been proposed and there is not yet global consensus on a single definition or operationalisation. The revised European consensus on the definition and diagnosis of sarcopenia requires low muscle strength (demonstrated by low handgrip strength or prolonged sit to stand time) to make a diagnosis of probable sarcopenia; low muscle mass can optionally be used to confirm the diagnosis. [7] At present, resistance exercise is the only intervention shown to be of benefit in sarcopenia.[2] However, this intervention is only partially effective at reversing the decline in muscle mass and strength, and not all older people are either willing or able to undertake resistance training. Alternative interventions are therefore required both to prevent and ameliorate sarcopenia and its deleterious consequences.

The pathophysiology of sarcopenia is complex, multifactorial, and incompletely understood. Impaired micro and macrovascular function, neuromuscular junction dysfunction, chronic inflammation and changes to the extracellular matrix have all been posited as being of importance. [7] Mitochondrial dysfunction, however, is postulated to play a central role in the aetiology of the condition. Mitochondrial numbers are low in sarcopenic muscle, and oxidative phosphorylation capacity is strikingly impaired, driven by reductions in expression and activity of all the major mitochondrial respiratory chain complexes. [8 9] *Ex vivo* mitochondrial oxidative capacity parallels decline in muscle

For peer review only

strength. [10] Furthermore, release of pro-apoptotic factors, morphological alterations (fission, swelling), energy stress via reduced ATP, and increased mitochondrial reactive oxygen species (ROS) emission (which react with and damage cellular components including contractile proteins) have all been reported during muscle atrophy in preclinical studies. [10] Interventions that target deficits in mitochondrial function therefore provide an attractive mechanism to treat sarcopenia.

The role of NAD+ in muscle function

NAD+ and its reduced form (NADH) plays a central role in cellular redox homeostasis and is a cofactor in multiple pathways of cellular metabolism. NADH is essential for ATP production via oxidative phosphorylation in mitochondria, but it is also now clear that NAD+ is a key signalling molecule for multiple pathways implicated in cellular ageing. [11] These include roles in protein deacetylation signalling via sirtuins, and DNA damage sensing (via poly-ADP-ribose polymerases; PARPs). These and other key NAD+ dependent systems such as the NADase CD38, which increases with ageing not only play important roles in cellular ageing, they also consume NAD+, leaving less for competing systems. NAD+ concentrations are lower at older age in multiple tissues, and lower concentrations are found in a wide range of degenerative diseases. [12] Recent data confirm that NAD+ concentrations are lower (by 32%) in muscle biopsies from older people with sarcopenia than in healthy, age-matched controls. [13] Therapies that increase NAD+ concentrations may therefore provide a way to reverse the changes commonly seen in ageing skeletal muscle which contribute to sarcopenia, including deficits in mitochondrial function. [11 12]

Acipimox is a nicotinic acid analogue and postulated to act as an NAD+ precursor. [14] Administration of acipimox may therefore be able to enhance skeletal muscle NAD concentrations in patients with sarcopenia, with consequent improvement in mitochondrial function and hence skeletal muscle function. Acipimox already has marketing authorisation for use as a lipid-lowering agent for patients with type IIb or type IV hyperlipidaemia who have not responded to other therapies. A recent

For peer review only

systematic review identified 23 randomised controlled trials assessing the effect of NAD precursors on measures of physical function.[11] Studies were small (2-8 participants). A total of 96 primary outcomes were assessed, 10 of which were in favour of an NAD precursor, 1 was in favour of placebo; the remainder were not statistically significant in any clear direction. Small studies of acipimox that enrolled healthy younger patients or those with left ventricular dysfunction did not show improvement in measures of physical function.[15-19]

In contrast, muscle biopsy data from a trial of 21 patients with type 2 diabetes mellitus showed that 2 weeks of acipimox treatment (250mg twice a day) produced significant upregulation of mitochondrial genes involved in oxidative phosphorylation, an increase in skeletal muscle ATP content, and an increase in ex-vivo mitochondrial respiration; no change in mitochondrial number measured by mtDNA copy number was noted.[14] The results of a placebo-controlled trial examining the impact of acipimox on mitochondrial function in patients with mitochondrial myopathy are awaited. [20]. However, none of these studies have assessed the presence of sarcopenia or frailty and enrolled younger or middle-aged participants.

Trial objectives

Trials to date have not tested acipimox or other NAD analogues in older people with sarcopenia or frailty. Testing whether acipimox has the expected biological effects in this population (which would be the target population for treatment in clinical practice) is a key step in ascertaining whether this therapy should be tested in larger randomised controlled trials for people with sarcopenia and frailty. The primary objective of this proof-of-concept experimental medicine study is therefore to test whether supplementation with acipimox improves skeletal muscle NAD concentrations. The secondary objectives are to test whether supplementation with acipimox improves mitochondrial respiratory chain function; to examine if individuals with low NAD concentrations in skeletal muscle

For peer review only

biopsy can be identified by less invasive biomarkers (whole blood NAD concentration); and to assess candidate outcomes for future randomised clinical trials in patients with sarcopenia and frailty.

Methods and Analysis

Trial design and Setting

This is an open-label, uncontrolled, before-and-after feasibility study. Participants will be approached through older people's medicine clinics, day units and rehabilitation units. In addition, potential participants will be identified through local primary care centres, acting as patient identification centres (PIC), and via a sarcopenia registry.[21] Screening visits will take place either in the participants' homes or in a clinical research facility. Study assessments will take place at the Clinical Ageing Research Unit and Clinical Research Facilities, based in Newcastle upon Tyne Hospitals NHS Foundation Trust.

Eligibility criteria

The target population are adults aged 65 years and over with sarcopenia. Sarcopenia is defined using hand grip strength or five times sit-to-stand thresholds from the 2019 European Working Group on Sarcopenia in Older People (EWGSOP) guidance.[7] In order to identify a frail / prefrail population, participants will also be required to have a walk speed <=0.8 m/s on a 4 metre walk test.[22] Participants must be able to give written informed consent. The study exclusion criteria are primarily focused on excluding participants for whom use of acipimox or aspirin may be unsafe or for whom MRI is contraindicated. Participants who are at risk of other types of skeletal muscle myopathy will be excluded to avoid confusion with the diagnosis of sarcopenia. The full inclusion and exclusion criteria for the study are shown in Table 1.

For peer review only

Interventions

All participants will take acipimox orally. Participants will be supplied with 250mg capsules, and the dose is adjusted according to renal function. Participants with creatinine clearance (estimated by the Cockcroft-Gault equation) >60 ml/min will be prescribed one capsule three times a day.[23] Participants with creatinine clearance 45-60 ml/min will be prescribed one capsule twice a day. Commonly reported adverse effects with acipimox are: headache, dyspepsia, abdominal discomfort, urticaria, asthenia and facial flushing. Facial flushing is likely to increase the risk of discontinuation of therapy and to cause unmasking of treatment allocation in any future randomised controlled trial. To suppress this side-effect, participants will be prescribed aspirin 75mg orally to be taken 30 minutes before the first dose of acipimox each day with the expectation that this intervention would also form part of a future randomised controlled trial as has been the case previously.[20] Participants who take aspirin as part of their usual medication regime will continue their usual dose and will not be prescribed additional aspirin. If acipimox is discontinued aspirin will be discontinued. Participants will start acipimox and aspirin 48 hours after the initial muscle biopsy is completed and continue the intervention until 24 hours prior to the second biopsy (3 weeks +/- 3 days). This dose duration of treatment was sufficient to upregulate mitochondrial genes involved in oxidative phosphorylation, increase in skeletal muscle ATP content, and increase ex-vivo mitochondrial respiration in a study of patients with type 2 diabetes mellitus. [14]

Investigators may discontinue the trial treatment in the event of intolerable side effects occurring that are possibly, probably, or definitely related to trial medication or which constitute a serious adverse reaction (SAR) or suspected unexpected serious adverse reaction (SUSAR). Treatment will also be discontinued if the participant requests the medication to be withdrawn, requests not to undergo follow-up study measurements or if creatinine clearance falls below 45 ml/min. If creatinine clearance falls from above 60ml/min to between 45 and 60ml/min during the study, the dose frequency will be

For peer review only

reduced from three times a day to twice a day. If acipimox is discontinued this will automatically lead to withdrawal from the trial.

Adherence

Participants will be asked to bring their unused trial medication to the follow up visit so that adherence can be calculated by tablet count. Adherence will be defined as number of tablets taken divided by number of tablets expected to have been taken.

Outcomes

Primary outcome

The primary outcome is change in skeletal muscle NAD+/NADH concentrations and ratio between baseline and follow up. To measure this outcome, muscle biopsy of vastus lateralis muscle will be performed at baseline and follow-up. Biopsies will be taken under local anaesthetic, using a Weil-Blakesley conchotome, a technique that has previously been shown to be well tolerated even by very old participants. [24-26] The second biopsy will be taken from the same leg as the first biopsy, a minimum of 3cm from the first biopsy site to avoid healing or inflammatory changes affecting the results of the second biopsy. Participants will be asked to withhold aspirin for 24 hours prior to biopsy. Aspirin (taken as part of their usual medication regime, or as adjunctive medication in the study to prevent flushing) can be started or restarted 48 hours after the biopsy. Biopsy samples will be weighed and snap frozen in liquid nitrogen cooled isopentane immediately after extraction, then stored at -80°C pending analysis. NAD+/NADH concentrations will be measured using a modified protocol of the Promega NAD/NADH-Glo assay kit (Promega, Southampton, UK).

For peer review only

Secondary outcomes

A range of measures of physical performance, mechanistic outcomes and outcomes related to trial performance will also be recorded at baseline and follow-up to assess the potential of these outcome for future randomised controlled trials.

Biopsy related outcomes

ATP/ADP concentrations and ratio will be assessed in the muscle biopsy samples according to Strehler[27], COX SDH histochemistry[28] and quadruple immunofluorescence[29] will be used to assess respiratory chain deficiency. Mitochondrial DNA copy number will be assessed via quantitative PCR[30]

Physical performance Measures

Maximal hand grip strength (measured using a Jamar Dynamometer) and the short physical performance battery will be performed at baseline and follow-up.[31 32]

Remote Digital Monitoring

Seven-day remote monitoring with a wearable device (AX6; Axivity Ltd, Newcastle upon Tyne, UK) will be used to measure indices of walking activity, gait speed, variability, and postural control at baseline and follow-up. [33] [34 35]

Magnetic resonance imaging and Magnetic resonance spectroscopy outcomes

MR spectroscopy will assess phosphocreatine recovery rate measured by 31P MRS of the calf. The full MRI protocol has been published previously. [36] Changes in NAD(P)H levels by 31P MRS will also be assessed as an exploratory outcome. MRI of the calf muscle will be conducted at baseline and follow-up. Quantitative Dixon MRI will assess for intra-muscular fat infiltration.[37 38] An exploratory,

For peer review only

diffusion weighted MRI technique - motor unit magnetic resonance imaging (MUMRI) - will be performed at rest to assess motor unit size via spontaneous muscle activity. [39]

Whole blood NAD concentration

Whole blood will be collected into EDTA tubes and frozen at -80°C. Samples will be analysed for NAD+/NADH concentrations and ratio using Q-NADMED Blood NAD+ and NADH assay kit (NADMED, Helsinki, Finland) to test whether a precision target population (individuals with low skeletal muscle NAD+/NADH ratios at biopsy) can be identified by less invasive biomarkers.

Participation timeline

Recruitment and data management

Recruitment will take place over 12 months and started in October 2022. Potential participants are sent or given a brief study information sheet with an invitation letter, a reply slip and a prepaid envelope to express interest. The reply slip is returned to the study team and those who are interested will be contacted for pre-screening as described below. Data including numbers of potential participants identified, approached, and pre-screened will be collected and documented on the site screening log. Data will be collected on a paper case report forms and transposed to and secure Excel database. The participant trial record, including completed paper data collection tools, will be archived at site for 15 years following the end of the trial.

Pre-screening

Participants who express an interest in the trial will enter a brief telephone pre-screening process to check provisional eligibility. The pre-screening form is shown Supplementary material 1. Verbal consent will be taken for the pre-screening process including access to medical records. The SARC-F questionnaire will be administered; [40] this comprises five questions on physical function giving a total score between zero and ten. Based on previous observational data and trial experience, a score

For peer review only

of one or more will be sufficient to identify those more likely to have sarcopenia and will enable progression to the screening visit.[41])[15] Participants who pass the pre-screen will be given, emailed or posted the full participant information sheet (PIS), and all participants will be given at least 48 hours to consider their participation. The PIS can be found in Supplementary material 2. The study team will then telephone the potential participant, and those wishing to take part will then be invited to a screening visit.

Screening

Written informed consent will be obtained at the screening visit, which may take place at home, in clinic, or in a research facility. After informed consent is given, information on demographics, medical history, and medication history (including past history of allergy to acipimox or niacin related products) will be collected. Maximum handgrip, five times sit to stand and 4 metre walk speed will be measured. [22] Screening blood will be taken to ensure it is safe to prescribe acipimox and perform muscle biopsy. A single 4 ml EDTA sample will be collected for Full blood count (FBC), and a single 8.5ml clot activator separation gel sample will be collected for urea, creatinine, and electrolytes (U&Es) and liver function tests (LFTs – bilirubin, alanine aminotransferase and alkaline phosphatase). Acceptable results are shown in Table 1. The vastus lateralis muscle will be inspected and palpated to ensure suitability for biopsy. Participants will be asked to confirm if they are taking part in any other interventional clinical trials or monitored exercise programs. Participant eligibility to proceed to the baseline visit is confirmed after the screening visit, once all screening assessment results are available.

Schedule of events

The schedule of events for the study is shown in Supplementary table 1. The primary and secondary outcomes are measured over two baseline visits and repeated at two follow-up visits.

For peer review only

Sample Size

As this is a feasibility study, few data exist on which to base a sample size calculation. The sample size has been selected to enable detection of a one standard deviation change in measures of mitochondrial NAD+/NADH ratios, ATP/ADP ratios or respiratory chain function. To detect this change with an alpha of 0.05 and 80% power requires 11 paired observations (using a paired t-test). We plan to recruit 16 participants to allow for dropout and non-completion of the course of medication.

Statistical analysis

Analysis Population

The primary analysis population is the per-protocol population. This will consist of all participants who do not discontinue acipimox and undergo both baseline and follow up assessment for a given outcome. A safety analysis will be performed examining adverse events for all participants receiving at least one dose of acipimox.

Statistical Analysis Plan

A full statistical analysis plan (SAP) will be developed during the trial, which will include evaluation of the mechanistic outcomes data and correlation with main study outcomes. This will be developed by the Trial Management Group. Descriptive baseline statistics for all enrolled participants and for those completing the primary analysis (the per-protocol group) will be generated.

Analysis of the Primary Outcome Measure

The primary outcome is change in skeletal muscle NAD+/NADH ratios between baseline and follow up. Results will be analysed using a paired t-test (if normally distributed) or a Wilcoxon signed-rank test (if not normally distributed).

For peer review only

Analysis of Secondary Outcome Measures

Continuous secondary endpoints will be analysed in a similar manner to the primary outcome. Binary secondary endpoints will be analysed with a logistic regression model. Correlation between the changes in skeletal muscle NAD+/NADH ratios and changes in selected biomarkers (phosphocreatine recovery rate by MR spectroscopy, NAD+/NADH ratios in blood) will be performed using Pearson's correlation coefficient (where measures are normally distributed) or Spearman's rho (where measures are not normally distributed)

Trial oversight

A Trial Management Group (TMG) will convene approximately monthly throughout the trial. Members will include the Chief Investigator, co-applicants, trial statistician, local site research staff and a Sponsor representative. The TMG undertakes the role of a Trial Steering Committee for this small, low risk CTIMP study. No independent data monitoring committee will be established given the low number of participants and the short duration of therapy. Instead, an interim safety review by the funder will examine the data from the first eight patients to complete the study.

Patient and Public Involvement

Patient consultation influenced the choice of outcomes. Patients told us both muscle biopsy and 31P MRS scanning would be acceptable even in this older, frailer population. They supported an early phase feasibility study to confirm this and improve our mechanistic understanding of how acipimox may improve muscle function. Results from this study will be used to inform design process for a future randomised controlled trial, which would be powered for patient centred outcomes such as physical performance and quality of life.

For peer review only

Harms

All adverse events (AEs) occurring from point of consent to end of trial participation will be recorded. Falls will not be recorded as AEs, but any injury resulting from a fall will be recorded as an adverse event. Mild pain/soreness for the first 48 hours after muscle biopsy and superficial bruising at the site are to be expected and will not be recorded as AEs. Investigations, interventions, procedures, and operations will not be recorded as AEs, but the underlying condition (if new) leading to the intervention will be recorded as an AE. Complications resulting from investigations, interventions, procedures, and operations will be recorded as AEs.

Consent

Written informed consent will be obtained at screening visit before conducting any trial procedures including screening assessment. A medically qualified Investigator, competent in obtaining informed consent for research studies and formally delegated to do so by the site Principal Investigator, by way of dated signatures on the site delegation log, will conduct a consent interview with the participant at clinic or in the participants own home if preferred by the participant.

Ancillary and post-trial care

No provision for continuation of trial medication will be made by the trial team or Sponsor. Acipimox is not licensed for the indication under study in this trial; any off-licence use of acipimox after the end of the trial would be the responsibility of the participant's usual primary or secondary care clinical team.

For peer review only

Ethics and dissemination

Ethics approval

A favourable ethical opinion has been granted from the UK Health Research Authority and Northeast – Tyne and Wear South Research Ethics Committee (IRAS 293565). The trial has also received approval from the UK Medicines and Healthcare products Regulatory Agency (EuDRACT 2021-000993-28). The trial has been included in the National Institute for Health Research Clinical Research Network (NIHR CRN) portfolio (CPMS49429). The trial Sponsor is the Newcastle Upon Tyne Hospitals NHS Foundation Trust, Freeman Hospital, Freeman Road, High Heaton, Newcastle upon Tyne, NE7 7DN. The trial Sponsor has delegated responsibility for trial management to the CI , including trial design; review and approval of all localised patient-facing documentation prior to implementation at each site; collection, analysis and interpretation of data; writing of the protocol publication and final clinical report manuscripts. This protocol manuscript is based on version 2.0 of the trial protocol (dated 2nd November 2021).

Dissemination policy

A final report of the trial will be provided to the Sponsor, Research Ethics Committee, and the trial Funder within one year of the end of the study. The trial results will be uploaded to the European Union Drug Regulating Authorities Clinical Trials (EudraCT) database, as per the European Commission's guidelines on posting and publication of result-related information within 12 months. The trial is registered on the ISRCTN trial database (ISRCTN87404878) and trial results will be made publicly available on the ISRCTN trial registry within 12 months of the end of the trial, defined as Last Patient Last Visit date. The final clinical trial report will be used for publication and presentation at scientific meetings. Summaries of results will also be made available to investigators for dissemination within their clinical areas and to the wider public, and a summary of results will be sent to all

For peer review only

participants along with their treatment allocation. We will hold a dissemination event for participants and their families to present and discuss the study results.

Access to data

Access to the full dataset will be limited to the TMG and to authors of the trial publication. At the end of the trial, a de-identified dataset will be prepared and stored by Newcastle University. Requests for data sharing with study teams outside Newcastle University or the study Sponsor, including international requests, will be considered by a Data Access Committee with representation from the Funder, Sponsor and the trial Chief Investigator.

Authors contributions

Overall study conception and design: MDW, AAS, KGH, GG, LR

MRI design: MB, KGH

Laboratory analysis design and processes: LB, GG, CW, HALT, HS, CM

Accelerometry data collection and analysis design: SDD, RR, LR

Recruitment and medication use design: CA, CMcD

Manuscript preparation: CMcD

Critical revision of manuscript: All authors

Funding

This work was funded by the UKRI Medical Research Council Confidence in Concept funding scheme (grant number MC_PC_19047)

Competing interests

S. Del Din reports consultancy activity with Hoffmann-La Roche Ltd. outside of this study.

For peer review only

Acknowledgements

CMcD, MDW, AAS, GG, SDD and LR acknowledge support from the National Institute for Health and Care Research (NIHR) Newcastle Biomedical Research Centre (BRC). The NIHR Newcastle BRC is a partnership between Newcastle Hospitals NHS Foundation Trust, Cumbria Northumberland Tyne and Wear NHS Foundation Trust, and Newcastle University. The views expressed are those of the authors and not necessarily those of the NIHR or the Department of Health and Social Care.

SDD and LR were also supported by the Mobilise-D project that has received funding from the Innovative Medicines Initiative 2 Joint Undertaking (JU) under grant agreement No. 820820. This JU receives support from the European Union's Horizon 2020 research and innovation program and the European Federation of Pharmaceutical Industries and Associations (EFPIA). SDD and LR were also supported by the Innovative Medicines Initiative 2 Joint Undertaking (IMI2 JU) project IDEA-FAST - Grant Agreement 853981. SDD and LR were supported by the NIHR/Wellcome Trust Clinical Research Facility (CRF) infrastructure at Newcastle upon Tyne Hospitals NHS Foundation Trust.

We acknowledge the contribution of our much-missed colleague Dr Richard Dodds, who sadly died before this work could be completed.

For peer review only

Tables

Table 1 Inclusion and Exclusion Criteria

**INCLUSION CRITERIA**
• Age 65 years or over
• Low maximum handgrip strength (<16kg for women, <27kg for men) OR five-times sit to stand time >15 seconds (inability to complete sit to stand test will count as time >15seconds)
• Walk speed <=0.8 m/s on 4 metre walk test
**EXCLUSION CRITERIA**
General:
• Allergy to acipimox or other niacin-related products
• Allergy or intolerance of aspirin
• Any contraindication to taking aspirin
• Unable to give written informed consent
• Currently enrolled in another intervention study (observational studies are permitted)
• Currently participating in supervised exercise classes or physiotherapy
• Any progressive neurological or malignant condition with life expectancy <6 months
Safety of investigations medications:
• Creatinine Clearance <45ml/min (by Cockcroft-Gault equation)
• Taking statin medication or fibrate medication
• Active peptic ulcer disease or dyspepsia
Safety of muscle biopsy and MRI:
• Platelets <100x10^{9}/L at screening (contraindication to muscle biopsy)
• Presence of a bleeding diathesis or use of oral or parenteral anticoagulant medication
• Antiplatelet agents other than low dose (75mg once daily) aspirin
• Contraindications to MRI scanning (mild claustrophobia is not a contraindication)
• Allergy to local anaesthetic (lidocaine)
• Unable to palpate vastus lateralis muscle to enable biopsy localisation
Other causes of skeletal myopathy:
• Liver function tests (bilirubin, ALT, alkaline phosphatase) > 3x ULN
• Symptomatic (NYHA class II-IV) chronic heart failure (diagnosed according to European Society of Cardiology guidelines)
• Severe COPD (GOLD stage IV)
• Known myositis or other established myopathy
• Self-reported weight loss of >10% in last 6 months (to exclude significant cachexia)
• Known uncontrolled thyrotoxicosis
• 7.5mg/day or greater prednisolone use (or equivalent)

For peer review only

References

1. Patel HP, Syddall HE, Jameson K, et al. Prevalence of sarcopenia in community-dwelling older people in the UK using the European Working Group on Sarcopenia in Older People (EWGSOP) definition: findings from the Hertfordshire Cohort Study (HCS). *Age and Ageing* 2013;42(3):378-84. doi: 10.1093/ageing/afs197
2. Cruz-Jentoft AJ, Landi F, Schneider SM, et al. Prevalence of and interventions for sarcopenia in ageing adults: a systematic review. Report of the International Sarcopenia Initiative (EWGSOP and IWGS). *Age and Ageing* 2014;43(6):748-59. doi: 10.1093/ageing/afu115
3. Lunt E, Ong T, Gordon AL, et al. The clinical usefulness of muscle mass and strength measures in older people: a systematic review. *Age and Ageing* 2021;50(1):88-95. doi: 10.1093/ageing/afaa123
4. Liu P, Hao QK, Hai S, et al. Sarcopenia as a predictor of all-cause mortality among community-dwelling older people: A systematic review and meta-analysis. *Maturitas* 2017;103:16-22. doi: 10.1016/j.maturitas.2017.04.007
5. Yeung SSY, Reijnierse EM, Pham VK, et al. Sarcopenia and its association with falls and fractures in older adults: A systematic review and meta-analysis. *Journal of Cachexia Sarcopenia and Muscle* 2019;10(3):485-500. doi: 10.1002/jcsm.12411
6. Pinedo-Villanueva R, Westbury LD, Syddall HE, et al. Health Care Costs Associated With Muscle Weakness: A UK Population-Based Estimate. *Calcified Tissue International* 2019;104(2):137-44. doi: 10.1007/s00223-018-0478-1
7. Cruz-Jentoft AJ, Bahat G, Bauer J, et al. Sarcopenia: revised European consensus on definition and diagnosis. *Age and ageing* 2019;48(1):16-31.
8. Bellanti F, Lo Buglio A, Vendemiale G. Mitochondrial Impairment in Sarcopenia. *Biology-Basel* 2021;10(1):31. doi: ARTN 31

10.3390/biology10010031

9. Boffoli D, Scacco SC, Vergari R, et al. Decline with Age of the Respiratory-Chain Activity in Human Skeletal-Muscle. *Biochimica Et Biophysica Acta-Molecular Basis of Disease* 1994;1226(1):73-82. doi: Doi 10.1016/0925-4439(94)90061-2
10. Coen PM, Musci RV, Hinkley JM, Miller BF. Mitochondria as a target for mitigating sarcopenia. *Frontiers in Physiology* 2019:1883.
11. Barker FJ, Hart A, Sayer AA, Witham MD. Effects of nicotinamide adenine dinucleotide precursors on measures of physical performance and physical frailty: A systematic review. *JCSM Clinical Reports* 2022;7(4):93-106.
12. Camacho-Pereira J, Tarragó MG, Chini CCS, et al. CD38 Dictates Age-Related NAD Decline and Mitochondrial Dysfunction through an SIRT3-Dependent Mechanism. *Cell Metabolism* 2016;23(6):1127-39. doi: 10.1016/j.cmet.2016.05.006
13. Migliavacca E, Tay SKH, Patel HP, et al. Mitochondrial oxidative capacity and NAD

biosynthesis are reduced in human sarcopenia across ethnicities. *Nature Communications* 2019;10(1):5808. doi: ARTN 5808

10.1038/s41467-019-13694-1 [published Online First: 20191220]

14. Van De Weijer T, Phielix E, Bilet L, et al. Evidence for a direct effect of the NAD+ precursor acipimox on muscle mitochondrial function in humans. *Diabetes* 2015;64(4):1193-201.
15. Gautier J, Pirnay F, Jandrain B, et al. Endogenous substrate oxidation during exercise and variations in breath 13CO2/12CO2. *Journal of Applied Physiology* 1993;74(1):133-38.

16. Head A, Jakeman P, Kendall M, et al. The impact of a short course of three lipid lowering drugs on fat oxidation during exercise in healthy volunteers. *Postgraduate medical journal* 1993;69(809):197-203.
17. Nellemann B, Søndergaard E, Jensen J, et al. Kinetics and utilization of lipid sources during acute exercise and acipimox. *American Journal of Physiology-Endocrinology and Metabolism* 2014;307(2):E199-E208.
18. Halbirk M, Nørrelund H, Møller N, et al. Suppression of circulating free fatty acids with acipimox in chronic heart failure patients changes whole body metabolism but does not affect cardiac function. *American Journal of Physiology-Heart and Circulatory Physiology* 2010;299(4):H1220-H25.
19. Makimura H, Stanley TL, Suresh C, et al. Metabolic Effects of Long-Term Reduction in Free Fatty Acids With Acipimox in Obesity: A Randomized Trial. *Journal of Clinical Endocrinology & Metabolism* 2016;101(3):1123-33. doi: 10.1210/jc.2015-3696
20. Abouhajar A, Alcock L, Bigirumurame T, et al. Acipimox in Mitochondrial Myopathy (AIMM): study protocol for a randomised, double-blinded, placebo-controlled, adaptive design trial of the efficacy of acipimox in adult patients with mitochondrial myopathy. *Trials* 2022;23(1):1-15.
21. Witham MD, Achison M, Aspray TJ, et al. Recruitment strategies for sarcopenia trials: lessons from the LACE randomized controlled trial. *JCSM Rapid Communications* 2021;4(2):93-102. doi: 10.1002/rco2.38
22. Abellan Van Kan G, Rolland Y, Andrieu S, et al. Gait speed at usual pace as a predictor of adverse outcomes in community-dwelling older people an International Academy on Nutrition and Aging (IANA) Task Force. *The journal of nutrition, health & aging* 2010;13(10):881-89. doi: 10.1007/s12603-009-0246-z
23. Cockcroft DW, Gault MH. Prediction of Creatinine Clearance from Serum Creatinine. *Nephron* 1976;16(1):31-41. doi: Doi 10.1159/000180580
24. Patel HP, Syddall HE, Martin HJ, et al. The Feasibility and Acceptability of Muscle Biopsy in Epidemiological Studies: Findings from the Hertfordshire Sarcopenia Study (Hss). *Journal of Nutrition Health & Aging* 2011;15(1):10-15. doi: DOI 10.1007/s12603-011-0006-8
25. Dodds RM, Hurst C, Hillman SJ, et al. Advancing our understanding of skeletal muscle across the lifecourse: Protocol for the MASS_Lifecourse study and characteristics of the first 80 participants. *Experimental Gerontology* 2022;166:111884. doi: ARTN 111884

10.1016/j.exger.2022.111884
26. Dodds RM, Davies K, Granic A, et al. Mitochondrial respiratory chain function and content are preserved in the skeletal muscle of active very old men and women. *Experimental Gerontology* 2018;113:80-85. doi: 10.1016/j.exger.2018.09.020
27. Strehler BL. Adenosine-5′-triphosphate and creatine phosphate determination with luciferase. Methods of enzymatic analysis: Elsevier 1974:2112-26.
28. Old SL, Johnson MA. Methods of Microphotometric Assay of Succinate-Dehydrogenase and Cytochrome-C Oxidase Activities for Use on Human Skeletal-Muscle. *Histochemical Journal* 1989;21(9-10):545-55. doi: Doi 10.1007/Bf01753355
29. Rocha MC, Grady JP, Grünewald A, et al. A novel immunofluorescent assay to investigate oxidative phosphorylation deficiency in mitochondrial myopathy: understanding mechanisms and improving diagnosis. *Scientific Reports* 2015;5(1):15037. doi: ARTN 15037

10.1038/srep15037
30. Grady JP, Murphy JL, Blakely EL, et al. Accurate Measurement of Mitochondrial DNA Deletion Level and Copy Number Differences in Human Skeletal Muscle. *Plos One* 2014;9(12):e114462. doi: ARTN e114462

10.1371/journal.pone.0114462

For peer review only

31. Roberts HC, Denison HJ, Martin HJ, et al. A review of the measurement of grip strength in clinical and epidemiological studies: towards a standardised approach. *Age and Ageing* 2011;40(4):423-29. doi: 10.1093/ageing/afr051
32. Guralnik JM, Simonsick EM, Ferrucci L, et al. A Short Physical Performance Battery Assessing Lower-Extremity Function - Association with Self-Reported Disability and Prediction of Mortality and Nursing-Home Admission. *Journals of Gerontology* 1994;49(2):M85-M94. doi: DOI 10.1093/geronj/49.2.M85
33. Axivity. AX6 Datasheet 6-Axis logging movement sensor 2023 [Available from: https://axivity.com/files/resources/AX6_Data_Sheet.pdf.
34. Del Din S, Galna B, Godfrey A, et al. Analysis of free-living gait in older adults with and without Parkinson's disease and with and without a history of falls: identifying generic and disease-specific characteristics. *The Journals of Gerontology: Series A* 2019;74(4):500-06.
35. Del Din S, Galna B, Lord S, et al. Falls risk in relation to activity exposure in high-risk older adults. *The Journals of Gerontology: Series A* 2020;75(6):1198-205.
36. Hollingsworth KG, Newton JL, Taylor R, et al. Pilot study of peripheral muscle function in primary biliary cirrhosis: Potential implications for fatigue pathogenesis. *Clinical Gastroenterology and Hepatology* 2008;6(9):1041-48. doi: 10.1016/j.cgh.2008.04.013 [published Online First: 20080808]
37. Loughran T, Higgins DM, McCallum M, et al. Improving highly accelerated fat fraction measurements for clinical trials in muscular dystrophy: origin and quantitative effect of R2* changes. *Radiology* 2015;275(2):570-78.
38. Burakiewicz J, Sinclair CDJ, Fischer D, et al. Quantifying fat replacement of muscle by quantitative MRI in muscular dystrophy. *Journal of Neurology* 2017;264(10):2053-67. doi: 10.1007/s00415-017-8547-3 [published Online First: 20170701]
39. Birkbeck MG, Blamire AM, Whittaker RG, et al. The role of novel motor unit magnetic resonance imaging to investigate motor unit activity in ageing skeletal muscle. *Journal of cachexia, sarcopenia and muscle* 2021;12(1):17-29.
40. Malmstrom TK, Morley JE. SARC-F: A Simple Questionnaire to Rapidly Diagnose Sarcopenia. *Journal of the American Medical Directors Association* 2013;14(8):531-32. doi: 10.1016/j.jamda.2013.05.018
41. Dodds RM, Murray JC, Robinson SM, Sayer AA. The identification of probable sarcopenia in early old age based on the SARC-F tool and clinical suspicion: findings from the 1946 British birth cohort. *European Geriatric Medicine* 2020;11(3):433-41. doi: 10.1007/s41999-020-00310-5 [published Online First: 20200319]

For peer review only

Supplementary Table 1 Schedule of events

	Visit 1	Visit 2	Visit 3	Visit 4*	Visit 5	Visit 6	Visit 7	Visit 8	Visit 9	Visit 10
	Pre-Screen	Screening	Baseline 1	Baseline 2	Phone call	Phone call	Follow up 1	Follow up 2	Phone call	Phone call
**Assessment**		Day -28 to -1	Day 0	Day 7 (+/- 1 day)	Day 9 (+/- 1 day)	Day 14 (+/- 2 days)	Day 21 (+/- 3 days)	Day 28 (+/- 3 days)	Day 30 (+/- 3 days)	Day 37 (+/- 4 days)
SARC-F questions	X									
Demographics	X	X								
Medical history	X	X								
Concomitant medications	X	X	X	X			X	X		
Informed written consent		X								
Height		X								
Weight		X								
Bloods (U+Es, liver function tests)		X								
Bloods (Full blood count)		X								
Bloods (research bloods for NAD levels)			X				X			
Frailty questions (activity, exhaustion, weight loss)			X				X			
Muscle biopsy				X				X		
MRI scanning			X				X			

For peer review only

Accelerometer given out			X				X			
Accelerometer returned				X				X		
Medication dispensing				X						
Instructions on taking medication					X				X	
Medication adherence (collection/count)								X		
Telephone check of wound healing					X	X			X	X
Adverse Events log completion		X	X	X	X	X	X	X	X	X
4m walk speed		X								
5-times sit to stand		X								
Grip strength		X	X				X			
Short Physical Performance Battery			X				X			
Eligibility confirmation		X								

For peer review only

For peer review only

Participant ID: A C - __ __ __

Acipimox to improve muscle function and sarcopenia – a feasibility study

EudraCT 2021-000993-28 Sponsor ref: 09768

Prescreening form

Centre/Participant ID: | A | C | – | | | |

For peer review only

PRESCREENING CONTACT

Date of prescreening contact | d | d | m | m | y | y | y | y |

Contact by: Telephone ☐ Face to face ☐

Age (years) ☐☐

Sex: Male ☐ Female ☐

SARC-F:

	Score
How much difficulty do you have in lifting and carrying 10 pounds? *(none=0, some=1, a lot or unable without help=2)*	
How much difficulty do you have walking across a room? *(none=0, some=1, a lot or unable without help=2)*	
How much difficulty do you have transferring from a chair or bed? *(none=0, some=1, a lot or unable without help=2)*	
How much difficulty do you have climbing a flight of 10 stairs? *(none=0, some=1, a lot or unable without help=2)*	
How many times have you fallen in the past year? *(none=0, 1-3 = 1, 4 or more = 2)*	
**Total score**	

Participant ID: A C - __ __ __

Active medical diagnoses

	Yes	No
Chronic heart failure		
Severe COPD (GOLD stage IV)		
Progressive neurological disease with life expectancy <6 mths		
Cancer with life expectancy <6 mths		
Myositis or other myopathy		
Active thyrotoxicosis		

Concomitant medications

	Yes	No
Statin		
Fibrate		
Acipimox		
Niacin		
>7.5mg/day oral prednisolone or equivalent		
Antiplatelet other than aspirin 75mg once daily		
Anticoagulants		
Previous intolerance of acipimox or niacin		

Weight (kg) [ ][ ].[ ]

Weight change in last 6 months (to nearest kg) (NB: 1 stone = 6kg): [+/-][ ][ ]

For peer review only - http://bmjopen.bmj.com/site/about/guidelines.xhtml

Participant ID: A C - __ __ __

Other eligibility criteria:

	Yes	No
Contraindications to MRI		
>10% weight loss over last 6 months		
Unwilling to have two muscle biopsies		

	Yes	No
Eligible to proceed in study?		
Participant happy for study team to access medical records?		
Participant happy to receive PIS?		
PIS sent?		

For peer review only

Research nurse / investigator signature: ________________________ Date: _ _ / _ _ /_ _ _ _

For peer review only - http://bmjopen.bmj.com/site/about/guidelines.xhtml

The Newcastle upon Tyne Hospitals NHS Foundation Trust

Acipimox to improve muscle function and sarcopenia – a feasibility study

Introduction

Thank you for considering taking part in the acipimox study. Before you decide whether to take part, please take time to read this information about the study carefully. Feel free to discuss it with your family, friends, carers or your GP if you wish to do so. If anything is unclear, or you need more information, please ask. You will find our contact details at the back of the information. This leaflet is yours to keep.

SUMMARY of the ACIPIMOX STUDY

**Purpose of the study**	• We would like to find out whether a medicine called acipimox (used to treat high cholesterol) can help to improve muscle function for people over the age of 65 with muscle weakness.
**Will I definitely get acipimox?**	• Yes. Everyone in the study will receive acipimox – no dummy (placebo) medication is used in this study.
**What are the side effects of the medicine?**	• Acipimox may cause some people to feel hot and red in the face (flushing) or cause a headache at the start of treatment but this usually goes away quickly. We will give you a mini-aspirin each day to stop this side-effect. Acipimox can cause nausea or indigestion in some people. A full list of side-effects are given later in this information sheet under Supporting Information
**If I take part, what will I have to do?**	• All of the study visits will take place in a research centre (the Clinical Ageing Research Unit)<br>• You will be asked to attend a screening visit. The doctor will discuss the study with you and you can ask questions. We will ask to take a small blood sample to see if it is safe for you to take part.

For peer review only - http://bmjopen.bmj.com/site/about/guidelines.xhtml

- If the study is suitable for you, we will ask you to take part in another four visits.
- We will ask you to take a tablet (acipimox) 2-3 times a day for 18 days together with aspirin once a day.
- At two of the visits, we will take a small sample of muscle tissue (a biopsy) from your thigh
- At two of the visits, we will do an MRI scan of your leg some of the scan will be at rest and other parts will be whilst you exercise your leg muscle in the scanner
- At these visits we will also ask you to do some simple tests of muscle strength and take a blood sample We will ask you to give small blood sample (a tablespoons worth)
- We will ask you to wear a sensor to measure your walking for a week before and after taking the course of tablets

Do I have to visit the hospital to take part?

- The study visits are at a research centre which is separate from the hospital. For this study you do need to come to the research centre.

Do I have to take part?

- No – it is entirely your choice whether you wish to take part or not. Your care will not be affected in any way.

If you are still interested in taking part in the study, please read on for more information.

For peer review only - http://bmjopen.bmj.com/site/about/guidelines.xhtml

The Newcastle upon Tyne Hospitals NHS Foundation Trust

What is the purpose of the study?

Many of us lose muscle size and strength as we get older – this is called sarcopenia. People with weaker muscles are more likely to fall over and may start to have problems carrying out normal daily activities. They may also take longer to recover from other illnesses. Falling, struggling with daily living and taking a long time to get better from being unwell is a condition called frailty.

The best way to keep up your muscle strength is to do strengthening exercises, but not everyone wants to, or is able to do these.

Acipimox is a medicine that is already safely used in older people to treat high cholesterol. Recent research suggests that levels of a naturally occurring substance called Nicotinamide Adenine Dinucleotide (NAD) are low in the muscles of people with sarcopenia. All cells in the body need NAD to produce and use energy. We think that acipimox might be able to increase the levels of NAD in muscle, which could in turn help muscles to work better for people with sarcopenia. To know if this is correct, we need to test the effects of acipimox on muscle function in people with signs of muscle weakness.

What are the possible side effects of the study medicine?

As with any medicine, the medicine used in this study (**acipimox**) may cause side effects in some people. Doctors around the world have used acipimox for nearly 40 years and the side effects are well understood.

In some people, acipimox can cause flushing (redness of the face) and headache. This side effect usually affects people for the first few doses but then gets better. To minimise this side effect, we will give you a low dose of aspirin to take once a day (if you already take aspirin, you will just keep taking your normal dose of aspirin). Less commonly, acipimox can cause nausea or indigestion. To help prevent this, we will ask you to take your study medicine during or just after meals.

Aspirin can cause inflammation of the stomach lining, and in rare cases can cause stomach ulcers. We are using a low dose of aspirin over a short period of time to minimise side effects, but if you have indigestion, stomach ulcers or are prone to bleeding, we will not include you in the study.

For peer review only - http://bmjopen.bmj.com/site/about/guidelines.xhtml

The 28-day follow-up visit is the last study visit. For the 28 days following on from this final visit, we would like to know if you have been unwell. We will ask you to contact your study doctor or a member of their team to tell them about this. You can contact your study doctor and team on the contact details given on the last page of this information sheet.

If you are unsure whether the study doctor needs to know about an illness or not, please get in touch with your study doctor or a member of their team to check.

What are the benefits and disadvantages of taking part in this study?

We cannot promise the study will help you directly. However, the information we collect from this study may help to improve the treatment for older people with muscle weakness. If you want to find out more about taking part in research studies, you can visit the NHS Choices website www.nhs.uk.

If you do get a side effect from the study medicine, your doctor can stop your medicine. The side effects should disappear rapidly.

The muscle biopsy will be taken after your skin and surrounding area is numbed by injecting local anaesthetic. The injection and biopsy may cause some minor discomfort and bruising after biopsy and may be uncomfortable for a few days. Muscle biopsy is a simple procedure removing only a small amount of muscle with a low risk of complications. In very rare cases permanent muscle weakness, infection or a patch of numbness might occur. See page 7 for more information.

Is it safe for me to take part in this study during COVID-19?

The study will only take place if Newcastle Hospitals says that it is safe to do so. The study team will follow all of the hospital COVID-19 policies and procedures when you visit, including wearing masks, gloves and visors. These will be explained to you when your appointments are arranged. The visits will take place at the Clinical Ageing Research Unit, which is on the old Newcastle General Hospital site. This means that you will not have to mix with other patients in the main hospitals.

For peer review only - http://bmjopen.bmj.com/site/about/guidelines.xhtml

Newcastle University

The Newcastle upon Tyne Hospitals NHS Foundation Trust

What would I have to do if I took part?

We will ask you to attend five visits at the Clinical Ageing Research Unit, on the old Newcastle General Hospital site:

- Screening visit
- First baseline visit (on day 0)
- Second baseline visit (on day 7)
- First follow-up visit (on day 21)
- Second follow-up visit (on day 28)

These visits are extra, and not part of your usual NHS care. There is a flow diagram (Figure 1) on page 10 that shows the details of the trial visits.

Screening Visit

At the screening visit the doctor will discuss the study with you, and you will be able to ask questions. This visit may take place in your home rather than in the research unit if you prefer. We will ask if you are happy to give written consent to take part in the study. We will ask you some questions about yourself, your medications and your health. We will also ask to take a small blood sample (2 teaspoons) to make sure that it is safe for you to take part in this study. We will time you walking a short distance (4 metres), ask you to stand and sit from a chair five times, and test your handgrip strength.

When we have the results of the blood test, we will call you and arrange the next visit.

Baseline Visit 1

At this first visit, we will do the following:

- Measure your weight and height.
- Time you walking a short distance (4 metres), with your usual walking aids.
- To test your balance, we will ask you to stand beside the researcher for a short time (no more than 30 seconds).

For peer review only - http://bmjopen.bmj.com/site/about/guidelines.xhtml

- Time you standing and sitting from a chair five times.
- Test the strength of your handgrip on both hands.
- Ask you questions about your activity level, weight change and levels of tiredness.
- Take a small amount of blood (6 teaspoons).
- Ask about other medicine that you may be taking.

We will then perform a magnetic resonance imaging (MRI) scan of your leg muscles whilst you exercise your leg.

The Magnetic Resonance Imaging (MRI) scan

You will need to arrive at the MRI centre 30 minutes before your arranged scan. Before your scan you will be asked questions to ensure that there are no known reasons why you should not undergo a scan, for example if you have a pacemaker fitted. For your scan you will be asked to remove items of jewellery, clothing with metal and all other metal (such as hair grips). For this reason you may prefer to leave valuable items such as jewellery at home, however lockers are available. You may also prefer to arrive at the MRI centre wearing loose fitting clothing, although if you prefer to change into comfortable clothing at the clinic changing rooms are available.

When you go through the MRI scanner, you will be asked to lie down on the scanner bed. We slide the bed into the scanner up to chest height and collect images of your thigh muscle. You will be asked to keep as still as possible while we do this so that we get good images. The scanner is noisy but we will provide earplugs. Each of these scans lasts about 1 minute.

We will then move the table so that your calf is in the middle of the scanner. We will collect some images of your calf muscles at rest, again you will be asked to keep as still as possible. We will then ask you to push against a pedal (an up down motion) for about 3 minutes. The effort required is similar to walking uphill on Dean Street (a steep hill in Newcastle). The radiographer will then ask you to stop pushing and keep still for about 6 minutes while we collect data from your leg muscle after the exercise. After this, your MRI scan is complete.

When having a scan, there is always a small possibility that an abnormality could be observed on the images of which you and your doctors were unaware. The MRI scans we collect will be reviewed by a radiologist in the

For peer review only - http://bmjopen.bmj.com/site/about/guidelines.xhtml

Newcastle upon Tyne Hospitals NHS Foundation Trust to look for any such findings.

It is important to recognise that the MRI scans are not being taken for diagnostic purposes and so there is no guarantee that the scans would be of the right kind to detect any abnormality which may be present.

Should the radiologist suspect anything abnormal on your scans they will inform the study Principal Investigator who will contact your clinical care team or GP in order to make recommendations about any further investigations which may be appropriate for them to arrange.

The walking sensor

After the scan, we will give you the sensor to wear. This is small, lightweight and is worn on the lower back. You will need to wear this all the time for the next seven days until you return for the second baseline visit. You do not need to take it off at night, and you can wear it in the shower. It will monitor your walking activity.

Baseline visit 2

At the second baseline visit, which will take place 7 days after the first visit, we will collect the accelerometer and then do a muscle biopsy. If you usually take aspirin we will ask you not to take this on the morning of your muscle biopsy.

The muscle biopsy:

This test involves taking a small tissue sample from one of your thigh muscles. You will meet with an experienced doctor who will explain about the muscle biopsy and check that you are willing to have this procedure performed. Throughout the study you are free to change your mind about taking part in any part of the study without having to give a reason why.

If you are willing to go ahead, the doctor will ask you questions about what medication you are currently taking, your general health and examine you. This is because if, for example, you had varicose veins over the site of where we would take the biopsy, or were taking medications that can cause bleeding, then it would not be safe to perform this and therefore you would not undergo this part of the study.

For peer review only - http://bmjopen.bmj.com/site/about/guidelines.xhtml

Muscle biopsy is a simple procedure removing only a small amount of muscle with a low risk of complications. One in a hundred people experience significant bleeding that may leave you with a bruise taking a few weeks to disappear. In very rare cases permanent muscle weakness, infection or a patch of numbness (one in a thousand people) might occur.

The doctor and the nurse helping with the muscle biopsy will take you to a treatment room, set up to take a small tissue sample (biopsy) from your thigh muscle.

The procedure takes no longer than 30 minutes. Whilst lying down, you will be given a local anaesthetic injection to numb the skin and surrounding area. When the area is numb, a small cut is made in the skin to allow the biopsy to be taken. The muscle biopsy will be done with a special type of forceps as shown in the photo, below. The local anaesthetic may cause a stinging sensation for a few seconds and you may feel a pulling sensation when the biopsy is taken.

The wound is so small that it will not require stitching but is closed using medical tape called “Steri-strips”. A plaster will then be applied to the area, and a tight bandage wrapped around your leg for two hours whilst you rest on a bed. This is done to reduce the chance of bruising and bleeding.

The biopsy site may be uncomfortable for a few days, if so, paracetamol or codeine is recommended for pain relief. Anti-inflammatory drugs such as aspirin or ibuprofen should be avoided in the first 48 hours. You should also avoid getting the area wet in the first 48 hours. The Steri-strips can be removed after 5 days.

You should not drive after the biopsy and again we will provide a taxi to take you home. You will be given an advice sheet informing you to avoid strenuous activities like running or weightlifting for the first 5 days after your biopsy as this may delay the healing process and result in bleeding. However, the biopsy should not limit routine household tasks.

Follow-up phone calls

A member of the study team will telephone you twice as follow-up: the first time one or two days after your muscle biopsy and the second time around a week later to ensure that you are well. They will check with you that the wound from the muscle biopsy has healed. If you have any concern at all

For peer review only - http://bmjopen.bmj.com/site/about/guidelines.xhtml

about the site of the muscle biopsy, then a member of the study team will arrange to visit you at home.

The study medication

At the end of this second baseline visit, we will give you the study medicines to take home with you. **Please wait until two days after the biopsy** before you take the first doses of medication; we will telephone you first to make sure that the biopsy wound is healing as it should before starting the medication.

Follow up visit 1

At the first follow-up visit, which will take place two weeks later, we will repeat all the tests (including the MRI scan) which we did at the first baseline visit.

Follow up visit 2

At the second follow-up visit, which will take place seven days after the first follow-up visit, we will collect the accelerometer and your study medications. We will then do the muscle biopsy again, and as before we will telephone you a couple of days later and then a week later to check that everything has healed without problems.

A summary of the study visits is shown in a flow diagram (Figure 1) on the next page.

For peer review only - http://bmjopen.bmj.com/site/about/guidelines.xhtml

Newcastle University

NHS
The Newcastle upon Tyne Hospitals
NHS Foundation Trust

Figure 1. Summary of acipimox study visits

Screening visit – 60 mins
Written informed consent with doctor
Medical history, other medicines
Physical tests (short walk, sit to stand and handgrip strength)
Blood sample (2 teaspoons)
After visit - eligibility for the study confirmed

Day 0: Baseline visit 1 - two hours
Height and weight
Physical tests (walking, balance, sit to stand and handgrip strength)
Questions on activity and tiredness
Blood sample (6 teaspoons)
Review of other medication and any episodes of ill health
MRI scan of leg
Start wearing walking sensor

Day 7: Baseline visit 2 – three hours (includes 2 hours rest after the biopsy)
Medicines review and review of any episodes of ill health
Return walking sensor
Muscle Biopsy
Receive study medication
After visit: telephone call at 48 hours and 7 days. Start taking medication at 48 hours

Day 21: Follow up visit 1 - two hours
Physical tests (walking, balance, sit to stand and handgrip strength)
Questions on activity and tiredness
Blood sample (6 teaspoons)
Review of other medication and any episodes of ill health
MRI scan of leg
Start wearing walking sensor

Day 28: Follow up visit 2 – three hours (includes 2 hours rest after the biopsy)
Medicines review and review of any episodes of ill health
Return walking sensor and study medication
Muscle Biopsy
After visit: telephone call at 48 hours and 7 days

For peer review only - http://bmjopen.bmj.com/site/about/guidelines.xhtml

What happens to my blood and muscle samples?

At the baseline and follow up visits, we take blood and muscle samples to analyse as part of the study. If there is any sample left over from these analyses, we ask your permission to store it in a licenced Newcastle University Biobank. Your consent to store these additional samples in the biobank is optional. Your biobank samples may be used in further research linked to this study. They may also be used by other researchers in different research studies. Your samples will be identified only using your unique study identity number. Researchers who use your samples will not know who you are. Other researchers may want to use parts of your biobank sample in animal or commercial (paid) research. You can give samples to the biobank but opt out of them being used for animal or paid research.

Pregnancy

We know that it is not possible for female participants over the age of 65 years to become pregnant.

However, if you are a male participant, we will ask you to inform us if your female partner becomes pregnant, or is breast-feeding an infant. We will ask for your consent to do this. We will also ask your partner to sign a consent form. This will allow the study team to collect safety information about their pregnancy and their baby.

What happens at the end of the study?

At the end of the study (4 weeks) you will stop taking the study medicines. You will continue to receive standard care like any other patient with your condition under the care of your GP and/or hospital doctor. If you were taking aspirin before the study, you will continue to take this as before.

When everyone has completed the study, we will analyse the results and we will tell you what the results are. We can either invite you to a study event to which we will invite you, or send you a written newsletter – whichever you prefer.

For peer review only - http://bmjopen.bmj.com/site/about/guidelines.xhtml

Newcastle University

NHS The Newcastle upon Tyne Hospitals NHS Foundation Trust

Who do I contact for further information?

We will be happy to answer any questions you, your family or your carers may have about any aspect of this clinical study. Please call the number at the end of this booklet to speak to the research team.

For peer review only

For peer review only - http://bmjopen.bmj.com/site/about/guidelines.xhtml

Newcastle University

The Newcastle upon Tyne Hospitals NHS Foundation Trust

SUPPORTING INFORMATION

Acipimox side effects

Very common – Flushing, headache, indigestion

Common – Stomach ache, tiredness, rash

Uncommon – Allergic reaction, rash or itching, wheezing, nausea, feeling hot or unwell, muscle aches

Other – loose stools, gritty eyes

Acipimox may cause a sensation of heat, flushing or itching, especially at the beginning of treatment; it may cause a rash or redness of the skin. These reactions usually disappear rapidly during the first day of treatment.

Why was I contacted?

We have contacted you because you are aged 65 or over and our measurements or questionnaire results suggest that your muscles are not as strong as they used to be. This trial is testing a medicine (acipimox) to see if we can prevent further weakening of muscles in people like you.

Do I have to take part?

No - it is up to you to decide whether or not to take part in this study. You do not have to take part. If you choose not to, you will continue to get the standard care arranged by your doctor.

If you agree to take part, we will ask you to sign a consent form. We will give you a copy of your signed consent form which is yours to keep. You can still change your mind and withdraw at any time without having to give a reason. If you decide not to take part, or withdraw from the study later on, your current or future medical care will not be affected in any way.

What does giving consent mean for me?

By signing a consent form, this means that you fully understand what taking part in the study means for you. That's why it is really important that you take as much time as you want to read this information sheet. Feel free to discuss the study with your family, friends or any healthcare professional. At the screening visit, you will be discuss the study with a study doctor, who will answer all of your questions.

For peer review only - http://bmjopen.bmj.com/site/about/guidelines.xhtml

Newcastle University

The Newcastle upon Tyne Hospitals NHS Foundation Trust

Will I know what treatment I am on?

Yes. Everyone in the study will receive acipimox – no dummy (placebo) medication is used in this study.

Who has checked the scientific quality of the study?

Independent experts at Newcastle University have checked the quality of the science used to plan this study. The study has also been checked and approved by the app committee ensures that when you take part in the study, your rights and wellbeing will be protected. The study has also been checked by the government Medicines and Healthcare products Regulatory Agency (MHRA). The MHRA are responsible for approving all studies involving medicines. The Health Research Authority gives final overall approval for the study. The Newcastle upon Tyne Hospitals NHS Foundation Trust is the study Sponsor, which means that they have overall responsibility for the study. The Sponsor has carefully checked all of the study documentation. The Sponsor has also assessed the risks of this study. This is to ensure that we are not doing anything harmful to you during the study and that your information is collected safely and stored securely.

What happens if relevant new information becomes available?

During the course of the study, if new information on the risks or benefits of acipimox becomes available, we will let you know at your next study visit. If this new information requires urgent action, we will contact you before your next visit. If necessary, we will then discuss whether you should or would like to withdraw from the study.

Will you tell my GP that I'm taking part in a clinical study?

Yes. With your permission, we will write to your GP to tell them that you are taking part in this study. Your hospital medical record will also show that you are taking part in a clinical study. It is important for your safety that that your GP practice and hospital medical records show that you took part in a clinical study. If we discover a new health problem during the study, we will tell you. With your permission, we will also tell your GP. Any blood test results from taking part in this study will also be added to your medical records. Your GP will be asked to let the study team know of any side effects from taking the medicine or if you have had any emergency hospital admissions.

For peer review only - http://bmjopen.bmj.com/site/about/guidelines.xhtml

Who has overall responsibility for the study?

The study Sponsor is the Newcastle upon Tyne Hospitals NHS Foundation Trust, who have overall responsibility for the study.

The doctor in charge of the study (the Chief Investigator) is Professor Miles Witham, a Consultant Geriatrician based in Newcastle upon Tyne.

Who is providing the study medications?

Acipimox and aspirin have been on the market for many years. We have bought the study medications from their manufacturers in the same way that the NHS would usually buy medications – the medications have not been supplied as a favour by the manufacturers.

What will happen to the results of the Acipimox study?

- The results will be published in medical journals and presented at meetings to other doctors, nurses, researchers and patients.
- A report will be written for the study funder.
- A report will be written for the Controlled Clinical Trials (ISRCTN) database.
- All study results that are published will be anonymous. This means that no-one will be able to find out who you are. Your identity will always be protected.
- The results will be available at the end of the study through publications, in the wider press and directly to patient groups.
- Fully anonymised data may be made available to other researchers to help inform other research studies.

What if I have a complaint or a problem occurs?

a) Complaints

If you have any concern or complaint about any aspect of this clinical study, please contact your local study team by phone, letter or email. Their contact details are listed at the end of this information sheet. If you are still unhappy and wish to raise your concerns with someone who is not directly involved in your care, you can contact Patient Advice and Liaison Service (PALS) for

For peer review only - http://bmjopen.bmj.com/site/about/guidelines.xhtml

confidential advice on any aspect of care on 0800 032 0202, or email northoftynepals@nhct.nhs.uk

b) Harm

In the unlikely event that you are harmed during the study and this is due to NHS staff neglect, you may have grounds for legal action and compensation. This is organised through the NHS Indemnity (insurance) scheme. You may need to pay for your own legal costs. NHS Indemnity does not offer no-fault compensation (for harm that is not anyone's fault).

Newcastle University also have indemnity arrangements. This covers Newcastle University staff involved in designing and managing the Acipimox study.

Will my taking part in the study be kept confidential?

Yes. All the information that you provide during the course of this study will be securely stored. Paper copies of your study information will be stored in locked files or rooms at your local hospital. Electronic copies of your study information will be stored on a secure, password-protected computer database. Only authorised members of the study team will be granted access to the database.

- At study visits, your name will not be written on completed test forms or questionnaires. Instead, we will use a study code number (called a Participant Unique Study Identifier). This number is unique for you. No-one else taking part in the study will have this number. This number will also be used in the study database. Only the study team at your hospital will be able to link this number back to you using your date of birth, name and NHS number
- The study team at your hospital will have access to your information during the study. They will use this information to contact you to organise study visits as well as for ongoing safety.
- You will not be named in any results, reports or on websites
- Very occasionally, information might be given during the study that, by law, we must pass on to others. For instance, information which suggested you or others were at risk of harm. In this case, confidentiality

For peer review only - http://bmjopen.bmj.com/site/about/guidelines.xhtml

Newcastle University

NHS The Newcastle upon Tyne Hospitals NHS Foundation Trust

would be broken so that we could pass this information to the relevant people. You would be informed of this.

- At the end of the study, all study information will be kept in a secure storage area for at least 15 years. This is called archiving. Archiving means that any queries about the running of the study can still be answered after the study has ended. All information will be held securely to make sure we protect your confidentiality. After the archiving period has ended, your information will be safely destroyed.
- If there are any unexpected serious side effects to the medicine, we would send details of this to the government medicines agency (MHRA). There is a specific form to do this, and only your study number will be sent to them.

Will you look at information from my existing medical records?

Yes. The study team at your hospital will be able to look at your GP and hospital medical records. They need to do this to collect information that is needed for you to take part in the study. For example, they will collect results of your blood tests, and prescriptions and health history.

Authorised people from Newcastle Hospitals NHS Foundation Trust or from the MHRA will also need to look at your medical records. This is to check that the study is being carried out to the correct standards. Everyone who looks at your medical records will have a duty of confidentiality to you as a research participant.

What will happen if I don't want to carry on with the study?

You can withdraw from the study completely at any time, for any reason. You do not have to tell the study team why you want to withdraw. You will always be fully cared for and supported in line with your GP and hospital's standard practice.
If you do give a reason for withdrawing from the study, we will ask if you are happy for us to record why you decided to withdraw.

If you withdraw from the study, we will keep the information about you that we have already collected. You are free to request that the study team

For peer review only - http://bmjopen.bmj.com/site/about/guidelines.xhtml

destroys all information donated by you. By destroying your information, this means that it cannot be used at all for the remainder of the study. However, if some of your information has already been used in calculations and reports, it would not be possible to remove that information from these.

If I stop taking the medicine, do I have to leave the trial?

If you become unwell or the medicine causes side effects that you cannot tolerate, your usual doctor or the study team may ask you to stop taking the study medicine. If you stop taking the medication, you will not continue in the trial and will not have the second set of scans, blood tests and muscle biopsy.

What happens if I lose the capacity to consent during the study?

During the study, if you lose the capacity to make your own decisions, we will stop your medicine and withdraw you from the study.

Who is funding the acipimox study?

The Medical Research Council are funding the study through money given to Newcastle University. The UK government funds the MRC to carry out research to improve knowledge of diseases, and treatments to improve health.

How have patients and the public been involved in the design of the study?

Volunteers from the local VOICE organisation (https://www.voice-global.org/) have given us feedback on designing studies for older people which we have used in designing this study and the information sheet.

Will my expenses be reimbursed?

Yes – we will pay for your travel expenses including providing a taxi if you need this to attend study visits at the hospital. Alternatively, transport may be arranged for you if your local hospital is able to offer this. The study team will manage any payments to reimburse costs to you and you may be asked to provide receipts for your travel.

Will I be paid for taking part?

For peer review only - http://bmjopen.bmj.com/site/about/guidelines.xhtml

We will not give you a payment for taking part, but we will pay for your transport and make sure that you have some food and drink at each trial visit.

HOW WILL WE USE INFORMATION ABOUT YOU?

We will need to use information from you, your medical records and your GP for this clinical research study.

This information will include your initials, date of birth, NHS number, name and contact details. People will use this information to do the research or to check your records to make sure that the research is being done properly.

People who do not need to know who you are will not be able to see your name or contact details. Your data will have a code number, called your unique study identifier, instead.

We will keep all information about you safe and secure.

Once we have finished the study, we will keep some of the data so we can check the results. We will write our reports in a way that no-one can work out that you took part in the study.

What are your choices about how your information is used?

- You can stop being part of the study at any time, without giving a reason, but we will keep information about you that we already have.
- We need to manage your records in specific ways for the research to be reliable. This means that we won't be able to let you see or change the data we hold about you.

Where can you find out more about how your information is used?

You can find out more about how we use your information:

- www.hra.nhs.uk/information-about-patients/
- our leaflet available from https://www.newcastle-hospitals.nhs.uk/help/privacy/

For peer review only - http://bmjopen.bmj.com/site/about/guidelines.xhtml

- by asking one of the study team
- by sending an email to the trial Sponsor Data Protection Officer at nuth.dpo@nhs.net

Further Information and contact details

If you have any further questions or would like further information about the study or rights of participants, please feel free to contact the people below.

They are also who you or a doctor should contact in the event of an emergency.

Professor Miles Witham, telephone 07555 450498

Clinical Ageing Research Unit, telephone 0191 208 1250

Thank you for reading this information sheet

This research is funded by the UK Medical Research Council through their Confidence in Concept funding stream. The views expressed are those of the author(s) and not necessarily those of the Medical Research Council.

For peer review only - http://bmjopen.bmj.com/site/about/guidelines.xhtml

Reporting checklist for protocol of a clinical trial.

Based on the SPIRIT guidelines.

Instructions to authors

Complete this checklist by entering the page numbers from your manuscript where readers will find each of the items listed below.

Your article may not currently address all the items on the checklist. Please modify your text to include the missing information. If you are certain that an item does not apply, please write "n/a" and provide a short explanation.

Upload your completed checklist as an extra file when you submit to a journal.

In your methods section, say that you used the SPIRITreporting guidelines, and cite them as:

Chan A-W, Tetzlaff JM, Gøtzsche PC, Altman DG, Mann H, Berlin J, Dickersin K, Hróbjartsson A, Schulz KF, Parulekar WR, Krleža-Jerić K, Laupacis A, Moher D. SPIRIT 2013 Explanation and Elaboration: Guidance for protocols of clinical trials. BMJ. 2013;346:e7586

		Reporting Item	Page Number
**Administrative information**			
Title	#1	Descriptive title identifying the study design, population, interventions, and, if applicable, trial acronym	1

For peer review only

Trial registration	#2a	Trial identifier and registry name. If not yet registered, name of intended registry	5
Trial registration: data set	#2b	All items from the World Health Organization Trial Registration Data Set	
Protocol version	#3	Date and version identifier	4 & 17
Funding	#4	Sources and types of financial, material, and other support	19
Roles and responsibilities: contributorship	#5a	Names, affiliations, and roles of protocol contributors	1-2, 19
Roles and responsibilities: sponsor contact information	#5b	Name and contact information for the trial sponsor	16
Roles and responsibilities: sponsor and funder	#5c	Role of study sponsor and funders, if any, in study design; collection, management, analysis, and interpretation of data; writing of the report; and the decision to submit the report for publication, including whether they will have ultimate authority over any of these activities	NA
Roles and responsibilities: committees	#5d	Composition, roles, and responsibilities of the coordinating centre, steering committee, endpoint adjudication committee, data management team, and	15

For peer review only

		other individuals or groups overseeing the trial, if applicable (see Item 21a for data monitoring committee)	
**Introduction**			
Background and rationale	#6a	Description of research question and justification for undertaking the trial, including summary of relevant studies (published and unpublished) examining benefits and harms for each intervention	6
Background and rationale: choice of comparators	#6b	Explanation for choice of comparators	NA
Objectives	#7	Specific objectives or hypotheses	8
Trial design	#8	Description of trial design including type of trial (eg, parallel group, crossover, factorial, single group), allocation ratio, and framework (eg, superiority, equivalence, non-inferiority, exploratory)	8
**Methods: Participants, interventions, and outcomes**			
Study setting	#9	Description of study settings (eg, community clinic, academic hospital) and list of countries where data will	8

For peer review only

		be collected. Reference to where list of study sites can be obtained	
Eligibility criteria	#10	Inclusion and exclusion criteria for participants. If applicable, eligibility criteria for study centres and individuals who will perform the interventions (eg, surgeons, psychotherapists)	9
Interventions: description	#11a	Interventions for each group with sufficient detail to allow replication, including how and when they will be administered	9
Interventions: modifications	#11b	Criteria for discontinuing or modifying allocated interventions for a given trial participant (eg, drug dose change in response to harms, participant request, or improving / worsening disease)	9
Interventions: adherance	#11c	Strategies to improve adherence to intervention protocols, and any procedures for monitoring adherence (eg, drug tablet return; laboratory tests)	22
Interventions: concomitant care	#11d	Relevant concomitant care and interventions that are permitted or prohibited during the trial	8&9
Outcomes	#12	Primary, secondary, and other outcomes, including the specific measurement variable (eg, systolic blood pressure), analysis metric (eg, change from baseline, final value, time to event), method of aggregation (eg, median, proportion), and time point for each outcome.	10-13

For peer review only

		Explanation of the clinical relevance of chosen efficacy and harm outcomes is strongly recommended	
Participant timeline	#13	Time schedule of enrolment, interventions (including any run-ins and washouts), assessments, and visits for participants. A schematic diagram is highly recommended (see Figure)	12-11
Sample size	#14	Estimated number of participants needed to achieve study objectives and how it was determined, including clinical and statistical assumptions supporting any sample size calculations	13-14
Recruitment	#15	Strategies for achieving adequate participant enrolment to reach target sample size	8
**Methods: Assignment of interventions (for controlled trials)**			
Allocation: sequence generation	#16a	Method of generating the allocation sequence (eg, computer-generated random numbers), and list of any factors for stratification. To reduce predictability of a random sequence, details of any planned restriction (eg, blocking) should be provided in a separate document that is unavailable to those who enrol participants or assign interventions	NA

For peer review only

Allocation concealment mechanism	#16b	Mechanism of implementing the allocation sequence (eg, central telephone; sequentially numbered, opaque, sealed envelopes), describing any steps to conceal the sequence until interventions are assigned	NA
Allocation: implementation	#16c	Who will generate the allocation sequence, who will enrol participants, and who will assign participants to interventions	NA
Blinding (masking)	#17a	Who will be blinded after assignment to interventions (eg, trial participants, care providers, outcome assessors, data analysts), and how	NA
Blinding (masking): emergency unblinding	#17b	If blinded, circumstances under which unblinding is permissible, and procedure for revealing a participant's allocated intervention during the trial	NA
**Methods: Data collection, management, and analysis**			
Data collection plan	#18a	Plans for assessment and collection of outcome, baseline, and other trial data, including any related processes to promote data quality (eg, duplicate measurements, training of assessors) and a description of study instruments (eg, questionnaires, laboratory tests) along with their reliability and validity,	12

For peer review only

		if known. Reference to where data collection forms can be found, if not in the protocol	
Data collection plan: retention	#18b	Plans to promote participant retention and complete follow-up, including list of any outcome data to be collected for participants who discontinue or deviate from intervention protocols	12
Data management	#19	Plans for data entry, coding, security, and storage, including any related processes to promote data quality (eg, double data entry; range checks for data values). Reference to where details of data management procedures can be found, if not in the protocol	12
Statistics: outcomes	#20a	Statistical methods for analysing primary and secondary outcomes. Reference to where other details of the statistical analysis plan can be found, if not in the protocol	14
Statistics: additional analyses	#20b	Methods for any additional analyses (eg, subgroup and adjusted analyses)	14
Statistics: analysis population and missing data	#20c	Definition of analysis population relating to protocol non-adherence (eg, as randomised analysis), and any statistical methods to handle missing data (eg, multiple imputation)	14
**Methods: Monitoring**			

For peer review only

Data monitoring: formal committee	#21a	Composition of data monitoring committee (DMC); summary of its role and reporting structure; statement of whether it is independent from the sponsor and competing interests; and reference to where further details about its charter can be found, if not in the protocol. Alternatively, an explanation of why a DMC is not needed	15
Data monitoring: interim analysis	#21b	Description of any interim analyses and stopping guidelines, including who will have access to these interim results and make the final decision to terminate the trial	15
Harms	#22	Plans for collecting, assessing, reporting, and managing solicited and spontaneously reported adverse events and other unintended effects of trial interventions or trial conduct	15
Auditing	#23	Frequency and procedures for auditing trial conduct, if any, and whether the process will be independent from investigators and the sponsor	15
**Ethics and dissemination**			
Research ethics approval	#24	Plans for seeking research ethics committee / institutional review board (REC / IRB) approval	16

For peer review only

Protocol amendments	#25	Plans for communicating important protocol modifications (eg, changes to eligibility criteria, outcomes, analyses) to relevant parties (eg, investigators, REC / IRBs, trial participants, trial registries, journals, regulators)	15
Consent or assent	#26a	Who will obtain informed consent or assent from potential trial participants or authorised surrogates, and how (see Item 32)	16
Consent or assent: ancillary studies	#26b	Additional consent provisions for collection and use of participant data and biological specimens in ancillary studies, if applicable	NA
Confidentiality	#27	How personal information about potential and enrolled participants will be collected, shared, and maintained in order to protect confidentiality before, during, and after the trial	11, 18 and ICF
Declaration of interests	#28	Financial and other competing interests for principal investigators for the overall trial and each study site	20
Data access	#29	Statement of who will have access to the final trial dataset, and disclosure of contractual agreements that limit such access for investigators	17
Ancillary and post trial care	#30	Provisions, if any, for ancillary and post-trial care, and for compensation to those who suffer harm from trial participation	16

For peer review only

Dissemination policy: trial results	#31a	Plans for investigators and sponsor to communicate trial results to participants, healthcare professionals, the public, and other relevant groups (eg, via publication, reporting in results databases, or other data sharing arrangements), including any publication restrictions	17
Dissemination policy: authorship	#31b	Authorship eligibility guidelines and any intended use of professional writers	16
Dissemination policy: reproducible research	#31c	Plans, if any, for granting public access to the full protocol, participant-level dataset, and statistical code	NA
**Appendices**			
Informed consent materials	#32	Model consent form and other related documentation given to participants and authorised surrogates	Separate attachment
Biological specimens	#33	Plans for collection, laboratory evaluation, and storage of biological specimens for genetic or molecular analysis in the current trial and for future use in ancillary studies, if applicable	9/10/11

None The SPIRIT Explanation and Elaboration paper is distributed under the terms of the Creative Commons Attribution License CC-BY-NC. This checklist can be completed online using https://www.goodreports.org/, a tool made by the EQUATOR Network in collaboration with Penelope.ai

For peer review only

BMJ Open

A study protocol to investigate if Acipimox improves muscle function and sarcopenia –an open-label, uncontrolled, before-and-after experimental medicine feasibility study in community dwelling older adults.

Journal:	*BMJ Open*
Manuscript ID	bmjopen-2023-076518.R2
Article Type:	Protocol
Date Submitted by the Author:	25-Jan-2024
Complete List of Authors:	McDonald, Claire; Newcastle University, AGE Research Group, Translational and Clinical Research Institute, Faculty of Medical Sciences; Newcastle Upon Tyne Hospitals NHS Foundation Trust, NIHR Newcastle Biomedical Research Centre<br>Alderson , Craig; Newcastle Upon Tyne Hospitals NHS Foundation Trust<br>Birkbeck, Matthew; Newcastle University, Newcastle Magnetic Resonance Centre Translational and Clinical Research Institute, ; Newcastle Upon Tyne Hospitals NHS Foundation Trust<br>Brown, Laura ; Newcastle University, Wellcome Centre for Mitochondrial Research, Translational and Clinical Research Institute<br>Del Din, Silvia; Newcastle University, Brain and Movement Research Group, Translational and Clinical Research Institute,<br>Gorman, Grainne; Newcastle University, Wellcome Centre for Mitochondrial Research, Translational and Clinical Research Institute<br>Hollingsworth, Kieren; Newcastle University, Newcastle Magnetic Resonance Centre Translational and Clinical Research Institute<br>Massarella, Clare; Newcastle Upon Tyne Hospitals NHS Foundation Trust<br>Rehman, Rana; Newcastle University, Brain and Movement Research Group, Translational and Clinical Research Institute<br>Rochester, Lynn; Newcastle University, Brain and Movement Research Group, Translational and Clinical Research Institute,<br>Sayer, Avan; Newcastle Upon Tyne Hospitals NHS Foundation Trust, NIHR Biomedical Research Centre; Newcastle University, AGE Research Group, Translational and Clinical Research Institute<br>Su, Huizhong; Newcastle University, Brain and Movement Research Group, Translational and Clinical Research Institute,<br>Tuppen, Helen ; Newcastle University, Wellcome Centre for Mitochondrial Research, Translational and Clinical Research Institute<br>Warren, Charlotte; Newcastle University, Wellcome Centre for Mitochondrial Research, Translational and Clinical Research Institute<br>Witham, Miles; Newcastle University, NIHR Newcastle Biomedical Research Centre; Newcastle University, AGE Research Group, Translational and Clinical Research Institute
Primary Subject	Geriatric medicine

Heading:	
Secondary Subject Heading:	Evidence based practice
Keywords:	Clinical Trial, Aging, Neuromuscular disease < NEUROLOGY

A study protocol to investigate if Acipimox improves muscle function and sarcopenia –an open-label, uncontrolled, before-and-after experimental medicine feasibility study in community dwelling older adults.

Authors

Claire McDonald[1,2,3]

Craig Alderson [4]

Matt G Birkbeck [4,5]

Laura Brown [6]

Silvia Del Din [2, 7]

Grainne Gorman [6]

Kieren G Hollingsworth [5]

Clare Massarella [6]

Rana Rehman [7]

Lynn Rochester [2, 4, 7]

Avan A Sayer [1, 2]

Huizhong Su [6]

Helen AL Tuppen [6]

Charlotte Warren [6]

Miles D Witham* [1, 2]

*Corresponding author

Affiliations

1. AGE Research Group, Translational and Clinical Research Institute, Faculty of Medical Sciences, Newcastle University, Newcastle, UK

For peer review only

2. NIHR Newcastle Biomedical Research Centre, Newcastle upon Tyne Hospitals NHS Foundation Trust, Cumbria, Northumberland, Tyne and Wear NHS Foundation Trust and Newcastle University
3. Gateshead Health NHS Foundation trust, Gateshead, UK.
4. Newcastle Upon Tyne Hospitals NHS Foundation Trust
5. Newcastle Magnetic Resonance Centre, Translational and Clinical Research Institute, Newcastle University
6. Wellcome Centre for Mitochondrial Research, Translational and Clinical Research Institute, Newcastle University
7. Brain and Movement Research Group, Translational and Clinical Research Institute, Newcastle University

Correspondence to:

Professor Miles D Witham, NIHR Newcastle Biomedical Research Centre, Newcastle University Campus for Ageing and Vitality, Newcastle upon Tyne, NE4 5PL, UK. Tel: +44 191 208 1317. Email: Miles.Witham@newcastle.ac.uk

For peer review only

Abstract

Introduction

Sarcopenia is the age associated loss of muscle mass and strength. Nicotinamide adenine dinucleotide (NAD) plays a central role in both mitochondrial function and cellular ageing processes implicated in sarcopenia. NAD concentrations are low in older people with sarcopenia, and increasing skeletal muscle NAD concentrations may offer a novel therapy for this condition. Acipimox is a licensed lipid-lowering agent known to act as an NAD precursor. This open-label, uncontrolled, before-and-after proof-of-concept experimental medicine study will test whether daily supplementation with acipimox improves skeletal muscle NAD concentrations.

Methods and analysis

Sixteen participants aged 65 and over with probable sarcopenia will receive acipimox 250 mg and aspirin 75mg orally daily for 4 weeks, with the frequency of acipimox administration being dependent on renal function. Muscle biopsy of the vastus lateralis and MRI scanning of the lower leg will be performed at baseline before starting acipimox and after 3 weeks of treatment. Adverse events will be recorded for the duration of the trial. The primary outcome, analysed in a per-protocol population, is the change in skeletal muscle NAD concentration between baseline and follow-up. Secondary outcomes include changes in phosphocreatine recovery rate by ^{31}P magnetic resonance spectroscopy, changes in physical performance and daily activity (hand grip strength, 4-metre walk and 7-day accelerometery), changes in skeletal muscle mitochondrial respiratory function, changes in skeletal muscle mitochondrial DNA copy number and changes in NAD concentrations in whole blood as a putative biomarker for future participant selection.

For peer review only

Ethics and dissemination

The trial is approved by the UK Medicines and Healthcare products Regulatory Agency (EuDRACT 2021-000993-28) UK Health Research Authority and Northeast – Tyne and Wear South Research Ethics Committee (IRAS 293565). Results will be made available to participants, their families, patients with sarcopenia, the public, regional and national clinical teams, and the international scientific community.

Strengths and limitations

1. A key strength is that our recruitment method targets older people with sarcopenia – a population traditionally hard to include in trials and with whom experimental medicine studies are not commonly performed.
2. Our use of muscle biopsy-based outcome measures to understand treatment mechanisms is another key methodological strength and will demonstrate feasibility of doing serial muscle biopsies in this population for future similar trials.
3. A limitation is that the study is not randomised, and larger randomised controlled trials will be required to assess efficacy if results from this proof-of-concept trial are promising.
4. Co-administration of aspirin with acipimox is a methodological limitation as this complicates efforts to understand the mechanism of any beneficial effect, although it should improve the tolerability and hence adherence to treatment.

Key words

Sarcopenia, Nicotinamide adenine dinucleotide, Acipimox, Clinical trial, Older people

Trial registration number

ISRCTN trial database (ISRCTN87404878)

For peer review only

Protocol

Acipimox feasibility study Clinical Trial Protocol V2 2/11/21

For peer review only

Introduction

Sarcopenia is the loss of muscle strength and mass that commonly accompanies ageing. It is a syndrome of major clinical importance for many older people and affects between 5 and 10% of those aged over 65 in the general population, with rates in excess of 30% among those living in care homes.[1 2] Sarcopenia is associated with an increased risk of falls, hospitalisation, need for care and earlier death.[3-5] The cost of sarcopenia to the UK health service has been estimated to be in the region of 2 billion pounds per year.[6]

In recent decades several definitions of sarcopenia have been proposed and there is not yet global consensus on a single definition or operationalisation. The revised European consensus on the definition and diagnosis of sarcopenia requires low muscle strength (demonstrated by low handgrip strength or prolonged sit to stand time) to make a diagnosis of probable sarcopenia; low muscle mass can optionally be used to confirm the diagnosis. [7] At present, resistance exercise is the only intervention shown to be of benefit in sarcopenia.[2] However, this intervention is only partially effective at reversing the decline in muscle mass and strength, and not all older people are either willing or able to undertake resistance training. Alternative interventions are therefore required both to prevent and ameliorate sarcopenia and its deleterious consequences.

The pathophysiology of sarcopenia is complex, multifactorial, and incompletely understood. Impaired micro and macrovascular function, neuromuscular junction dysfunction, chronic inflammation and changes to the extracellular matrix have all been posited as being of importance. [7] Mitochondrial dysfunction, however, is postulated to play a central role in the aetiology of the condition. Mitochondrial numbers are low in sarcopenic muscle, and oxidative phosphorylation capacity is strikingly impaired, driven by reductions in expression and activity of all the major mitochondrial respiratory chain complexes. [8 9] *Ex vivo* mitochondrial oxidative capacity parallels decline in muscle

For peer review only

strength. [10] Furthermore, release of pro-apoptotic factors, morphological alterations (fission, swelling), energy stress via reduced ATP, and increased mitochondrial reactive oxygen species (ROS) emission (which react with and damage cellular components including contractile proteins) have all been reported during muscle atrophy in preclinical studies. [10] Interventions that target deficits in mitochondrial function therefore provide an attractive mechanism to treat sarcopenia.

The role of NAD+ in muscle function

NAD+ and its reduced form (NADH) plays a central role in cellular redox homeostasis and is a cofactor in multiple pathways of cellular metabolism. NADH is essential for ATP production via oxidative phosphorylation in mitochondria, but it is also now clear that NAD+ is a key signalling molecule for multiple pathways implicated in cellular ageing. [11] These include roles in protein deacetylation signalling via sirtuins, and DNA damage sensing (via poly-ADP-ribose polymerases; PARPs). These and other key NAD+ dependent systems such as the NADase CD38, which increases with ageing not only play important roles in cellular ageing, they also consume NAD+, leaving less for competing systems. NAD+ concentrations are lower at older age in multiple tissues, and lower concentrations are found in a wide range of degenerative diseases. [12] Recent data confirm that NAD+ concentrations are lower (by 32%) in muscle biopsies from older people with sarcopenia than in healthy, age-matched controls. [13] Therapies that increase NAD+ concentrations may therefore provide a way to reverse the changes commonly seen in ageing skeletal muscle which contribute to sarcopenia, including deficits in mitochondrial function. [11 12]

Acipimox is a nicotinic acid analogue and postulated to act as an NAD+ precursor. [14] Administration of acipimox may therefore be able to enhance skeletal muscle NAD concentrations in patients with sarcopenia, with consequent improvement in mitochondrial function and hence skeletal muscle function. Acipimox already has marketing authorisation for use as a lipid-lowering agent for patients with type IIb or type IV hyperlipidaemia who have not responded to other therapies. A recent

For peer review only

systematic review identified 23 randomised controlled trials assessing the effect of NAD precursors on measures of physical function.[11] Studies were small (2-8 participants). A total of 96 primary outcomes were assessed, 10 of which were in favour of an NAD precursor, 1 was in favour of placebo; the remainder were not statistically significant in any clear direction. Small studies of acipimox that enrolled healthy younger patients or those with left ventricular dysfunction did not show improvement in measures of physical function.[15-19]

In contrast, muscle biopsy data from a trial of 21 patients with type 2 diabetes mellitus showed that 2 weeks of acipimox treatment (250mg twice a day) produced significant upregulation of mitochondrial genes involved in oxidative phosphorylation, an increase in skeletal muscle ATP content, and an increase in ex-vivo mitochondrial respiration; no change in mitochondrial number measured by mtDNA copy number was noted.[14] The results of a placebo-controlled trial examining the impact of acipimox on mitochondrial function in patients with mitochondrial myopathy are awaited. [20]. However, none of these studies have assessed the presence of sarcopenia or frailty and enrolled younger or middle-aged participants.

Trial objectives

Trials to date have not tested acipimox or other NAD analogues in older people with sarcopenia or frailty. Testing whether acipimox has the expected biological effects in this population (which would be the target population for treatment in clinical practice) is a key step in ascertaining whether this therapy should be tested in larger randomised controlled trials for people with sarcopenia and frailty. The primary objective of this proof-of-concept experimental medicine study is therefore to test whether supplementation with acipimox improves skeletal muscle NAD concentrations. The secondary objectives are to test whether supplementation with acipimox improves mitochondrial respiratory chain function; to examine if individuals with low NAD concentrations in skeletal muscle

For peer review only

biopsy can be identified by less invasive biomarkers (whole blood NAD concentration); and to assess candidate outcomes for future randomised clinical trials in patients with sarcopenia and frailty.

Methods and Analysis

Trial design and Setting

This is an open-label, uncontrolled, before-and-after feasibility study. Participants will be approached through older people's medicine clinics, day units and rehabilitation units. In addition, potential participants will be identified through local primary care centres, acting as patient identification centres (PIC), and via a sarcopenia registry.[21] Screening visits will take place either in the participants' homes or in a clinical research facility. Study assessments will take place at the Clinical Ageing Research Unit and Clinical Research Facilities, based in Newcastle upon Tyne Hospitals NHS Foundation Trust.

Eligibility criteria

The target population are adults aged 65 years and over with sarcopenia. Sarcopenia is defined using hand grip strength or five times sit-to-stand thresholds from the 2019 European Working Group on Sarcopenia in Older People (EWGSOP) guidance.[7] In order to identify a frail / prefrail population, participants will also be required to have a walk speed <=0.8 m/s on a 4 metre walk test.[22] Participants must be able to give written informed consent. The study exclusion criteria are primarily focused on excluding participants for whom use of acipimox or aspirin may be unsafe or for whom MRI is contraindicated. Participants who are at risk of other types of skeletal muscle myopathy will be excluded to avoid confusion with the diagnosis of sarcopenia. The full inclusion and exclusion criteria for the study are shown in Table 1.

For peer review only

Interventions

All participants will take acipimox orally. Participants will be supplied with 250mg capsules, and the dose is adjusted according to renal function. Participants with creatinine clearance (estimated by the Cockcroft-Gault equation) >60 ml/min will be prescribed one capsule three times a day.[23] Participants with creatinine clearance 45-60 ml/min will be prescribed one capsule twice a day. Commonly reported adverse effects with acipimox are: headache, dyspepsia, abdominal discomfort, urticaria, asthenia and facial flushing. Facial flushing is likely to increase the risk of discontinuation of therapy and to cause unmasking of treatment allocation in any future randomised controlled trial. To suppress this side-effect, participants will be prescribed aspirin 75mg orally to be taken 30 minutes before the first dose of acipimox each day with the expectation that this intervention would also form part of a future randomised controlled trial as has been the case previously.[20] Participants who take aspirin as part of their usual medication regime will continue their usual dose and will not be prescribed additional aspirin. If acipimox is discontinued aspirin will be discontinued. Participants will start acipimox and aspirin 48 hours after the initial muscle biopsy is completed and continue the intervention until 24 hours prior to the second biopsy (3 weeks +/- 3 days). This dose duration of treatment was sufficient to upregulate mitochondrial genes involved in oxidative phosphorylation, increase in skeletal muscle ATP content, and increase ex-vivo mitochondrial respiration in a study of patients with type 2 diabetes mellitus. [14]

For peer review only

Investigators may discontinue the trial treatment in the event of intolerable side effects occurring that are possibly, probably, or definitely related to trial medication or which constitute a serious adverse reaction (SAR) or suspected unexpected serious adverse reaction (SUSAR). Treatment will also be discontinued if the participant requests the medication to be withdrawn, requests not to undergo follow-up study measurements or if creatinine clearance falls below 45 ml/min. If creatinine clearance falls from above 60ml/min to between 45 and 60ml/min during the study, the dose frequency will be

reduced from three times a day to twice a day. If acipimox is discontinued this will automatically lead to withdrawal from the trial.

Adherence

Participants will be asked to bring their unused trial medication to the follow up visit so that adherence can be calculated by tablet count. Adherence will be defined as number of tablets taken divided by number of tablets expected to have been taken.

Outcomes

Primary outcome

The primary outcome is change in skeletal muscle NAD+/NADH concentrations and ratio between baseline and follow up. To measure this outcome, muscle biopsy of vastus lateralis muscle will be performed at baseline and follow-up. Biopsies will be taken under local anaesthetic, using a Weil-Blakesley conchotome, a technique that has previously been shown to be well tolerated even by very old participants. [24-26] The second biopsy will be taken from the same leg as the first biopsy, a minimum of 3cm from the first biopsy site to avoid healing or inflammatory changes affecting the results of the second biopsy. Participants will be asked to withhold aspirin for 24 hours prior to biopsy. Aspirin (taken as part of their usual medication regime, or as adjunctive medication in the study to prevent flushing) can be started or restarted 48 hours after the biopsy. Biopsy samples will be weighed and snap frozen in liquid nitrogen cooled isopentane immediately after extraction, then stored at -80°C pending analysis. NAD+/NADH concentrations will be measured using a modified protocol of the Promega NAD/NADH-Glo assay kit (Promega, Southampton, UK).

For peer review only

Secondary outcomes

A range of measures of physical performance, mechanistic outcomes and outcomes related to trial performance will also be recorded at baseline and follow-up to assess the potential of these outcome for future randomised controlled trials.

Biopsy related outcomes

ATP/ADP concentrations and ratio will be assessed in the muscle biopsy samples according to Strehler[27], COX SDH histochemistry[28] and quadruple immunofluorescence[29] will be used to assess respiratory chain deficiency. Mitochondrial DNA copy number will be assessed via quantitative PCR[30]

Physical performance Measures

Maximal hand grip strength (measured using a Jamar Dynamometer) and the short physical performance battery will be performed at baseline and follow-up.[31 32]

Remote Digital Monitoring

Seven-day remote monitoring with a wearable device (AX6; Axivity Ltd, Newcastle upon Tyne, UK) will be used to measure indices of walking activity, gait speed, variability, and postural control at baseline and follow-up. [33] [34 35]

Magnetic resonance imaging and Magnetic resonance spectroscopy outcomes

MR spectroscopy will assess phosphocreatine recovery rate measured by 31P MRS of the calf. The full MRI protocol has been published previously. [36] Changes in NAD(P)H levels by 31P MRS will also be assessed as an exploratory outcome. MRI of the calf muscle will be conducted at baseline and follow-up. Quantitative Dixon MRI will assess for intra-muscular fat infiltration.[37 38] An exploratory,

For peer review only

diffusion weighted MRI technique - motor unit magnetic resonance imaging (MUMRI) - will be performed at rest to assess motor unit size via spontaneous muscle activity. [39]

Whole blood NAD concentration

Whole blood will be collected into EDTA tubes and frozen at -80°C. Samples will be analysed for NAD+/NADH concentrations and ratio using Q-NADMED Blood NAD+ and NADH assay kit (NADMED, Helsinki, Finland) to test whether a precision target population (individuals with low skeletal muscle NAD+/NADH ratios at biopsy) can be identified by less invasive biomarkers.

Participation timeline

Recruitment and data management

Recruitment will take place over 12 months and started in October 2022. Potential participants are sent or given a brief study information sheet with an invitation letter, a reply slip and a prepaid envelope to express interest. The reply slip is returned to the study team and those who are interested will be contacted for pre-screening as described below. Data including numbers of potential participants identified, approached, and pre-screened will be collected and documented on the site screening log. Data will be collected on a paper case report forms and transposed to and secure Excel database. The participant trial record, including completed paper data collection tools, will be archived at site for 15 years following the end of the trial.

Pre-screening

Participants who express an interest in the trial will enter a brief telephone pre-screening process to check provisional eligibility. The pre-screening form is shown Supplementary material 1. Verbal consent will be taken for the pre-screening process including access to medical records. The SARC-F questionnaire will be administered; [40] this comprises five questions on physical function giving a total score between zero and ten. Based on previous observational data and trial experience, a score

For peer review only

of one or more will be sufficient to identify those more likely to have sarcopenia and will enable progression to the screening visit.[41])[15] Participants who pass the pre-screen will be given, emailed or posted the full participant information sheet (PIS), and all participants will be given at least 48 hours to consider their participation. The PIS can be found in Supplementary material 2. The study team will then telephone the potential participant, and those wishing to take part will then be invited to a screening visit.

Screening

Written informed consent will be obtained at the screening visit, which may take place at home, in clinic, or in a research facility. After informed consent is given, information on demographics, medical history, and medication history (including past history of allergy to acipimox or niacin related products) will be collected. Maximum handgrip, five times sit to stand and 4 metre walk speed will be measured. [22] Screening blood will be taken to ensure it is safe to prescribe acipimox and perform muscle biopsy. A single 4 ml EDTA sample will be collected for Full blood count (FBC), and a single 8.5ml clot activator separation gel sample will be collected for urea, creatinine, and electrolytes (U&Es) and liver function tests (LFTs – bilirubin, alanine aminotransferase and alkaline phosphatase). Acceptable results are shown in Table 1. The vastus lateralis muscle will be inspected and palpated to ensure suitability for biopsy. Participants will be asked to confirm if they are taking part in any other interventional clinical trials or monitored exercise programs. Participant eligibility to proceed to the baseline visit is confirmed after the screening visit, once all screening assessment results are available.

Schedule of events

The schedule of events for the study is shown in Supplementary table 1. The primary and secondary outcomes are measured over two baseline visits and repeated at two follow-up visits.

For peer review only

Sample Size

As this is a feasibility study, few data exist on which to base a sample size calculation. The sample size has been selected to enable detection of a one standard deviation change in measures of mitochondrial NAD+/NADH ratios, ATP/ADP ratios or respiratory chain function. To detect this change with an alpha of 0.05 and 80% power requires 11 paired observations (using a paired t-test). We plan to recruit 16 participants to allow for dropout and non-completion of the course of medication.

Statistical analysis

Analysis Population

The primary analysis population is the per-protocol population. This will consist of all participants who do not discontinue acipimox and undergo both baseline and follow up assessment for a given outcome. A safety analysis will be performed examining adverse events for all participants receiving at least one dose of acipimox.

Statistical Analysis Plan

A full statistical analysis plan (SAP) will be developed during the trial, which will include evaluation of the mechanistic outcomes data and correlation with main study outcomes. This will be developed by the Trial Management Group. Descriptive baseline statistics for all enrolled participants and for those completing the primary analysis (the per-protocol group) will be generated.

Analysis of the Primary Outcome Measure

The primary outcome is change in skeletal muscle NAD+/NADH ratios between baseline and follow up. Results will be analysed using a paired t-test (if normally distributed) or a Wilcoxon signed-rank test (if not normally distributed).

For peer review only

Analysis of Secondary Outcome Measures

Continuous secondary endpoints will be analysed in a similar manner to the primary outcome. Binary secondary endpoints will be analysed with a logistic regression model. Correlation between the changes in skeletal muscle NAD+/NADH ratios and changes in selected biomarkers (phosphocreatine recovery rate by MR spectroscopy, NAD+/NADH ratios in blood) will be performed using Pearson's correlation coefficient (where measures are normally distributed) or Spearman's rho (where measures are not normally distributed)

Trial oversight

A Trial Management Group (TMG) will convene approximately monthly throughout the trial. Members will include the Chief Investigator, co-applicants, trial statistician, local site research staff and a Sponsor representative. The TMG undertakes the role of a Trial Steering Committee for this small, low risk CTIMP study. No independent data monitoring committee will be established given the low number of participants and the short duration of therapy. Instead, an interim safety review by the funder will examine the data from the first eight patients to complete the study.

Patient and Public Involvement

Patient consultation influenced the choice of outcomes. Patients told us both muscle biopsy and 31P MRS scanning would be acceptable even in this older, frailer population. They supported an early phase feasibility study to confirm this and improve our mechanistic understanding of how acipimox may improve muscle function. Results from this study will be used to inform design process for a future randomised controlled trial, which would be powered for patient centred outcomes such as physical performance and quality of life.

For peer review only

Harms

All adverse events (AEs) occurring from point of consent to end of trial participation will be recorded. Falls will not be recorded as AEs, but any injury resulting from a fall will be recorded as an adverse event. Mild pain/soreness for the first 48 hours after muscle biopsy and superficial bruising at the site are to be expected and will not be recorded as AEs. Investigations, interventions, procedures, and operations will not be recorded as AEs, but the underlying condition (if new) leading to the intervention will be recorded as an AE. Complications resulting from investigations, interventions, procedures, and operations will be recorded as AEs.

Consent

Written informed consent will be obtained at screening visit before conducting any trial procedures including screening assessment. A medically qualified Investigator, competent in obtaining informed consent for research studies and formally delegated to do so by the site Principal Investigator, by way of dated signatures on the site delegation log, will conduct a consent interview with the participant at clinic or in the participants own home if preferred by the participant.

Ancillary and post-trial care

No provision for continuation of trial medication will be made by the trial team or Sponsor. Acipimox is not licensed for the indication under study in this trial; any off-licence use of acipimox after the end of the trial would be the responsibility of the participant's usual primary or secondary care clinical team.

For peer review only

Ethics and dissemination

Ethics approval

A favourable ethical opinion has been granted from the UK Health Research Authority and Northeast – Tyne and Wear South Research Ethics Committee (IRAS 293565). The trial has also received approval from the UK Medicines and Healthcare products Regulatory Agency (EuDRACT 2021-000993-28). The trial has been included in the National Institute for Health Research Clinical Research Network (NIHR CRN) portfolio (CPMS49429). The trial Sponsor is the Newcastle Upon Tyne Hospitals NHS Foundation Trust, Freeman Hospital, Freeman Road, High Heaton, Newcastle upon Tyne, NE7 7DN. The trial Sponsor has delegated responsibility for trial management to the CI , including trial design; review and approval of all localised patient-facing documentation prior to implementation at each site; collection, analysis and interpretation of data; writing of the protocol publication and final clinical report manuscripts. This protocol manuscript is based on version 2.0 of the trial protocol (dated 2nd November 2021).

Dissemination policy

A final report of the trial will be provided to the Sponsor, Research Ethics Committee, and the trial Funder within one year of the end of the study. The trial results will be uploaded to the European Union Drug Regulating Authorities Clinical Trials (EudraCT) database, as per the European Commission's guidelines on posting and publication of result-related information within 12 months. The trial is registered on the ISRCTN trial database (ISRCTN87404878) and trial results will be made publicly available on the ISRCTN trial registry within 12 months of the end of the trial, defined as Last Patient Last Visit date. The final clinical trial report will be used for publication and presentation at scientific meetings. Summaries of results will also be made available to investigators for dissemination within their clinical areas and to the wider public, and a summary of results will be sent to all

For peer review only

participants along with their treatment allocation. We will hold a dissemination event for participants and their families to present and discuss the study results.

Access to data

Access to the full dataset will be limited to the TMG and to authors of the trial publication. At the end of the trial, a de-identified dataset will be prepared and stored by Newcastle University. Requests for data sharing with study teams outside Newcastle University or the study Sponsor, including international requests, will be considered by a Data Access Committee with representation from the Funder, Sponsor and the trial Chief Investigator.

Authors contributions

Overall study conception and design: MDW, AAS, KGH, GG, LR

MRI design: MB, KGH

Laboratory analysis design and processes: LB, GG, CW, HALT, HS, CM

Accelerometry data collection and analysis design: SDD, RR, LR

Recruitment and medication use design: CA, CMcD

Manuscript preparation: CMcD

Critical revision of manuscript: All authors

Funding

This work was funded by the UKRI Medical Research Council Confidence in Concept funding scheme (grant number MC_PC_19047)

Competing interests

S. Del Din reports consultancy activity with Hoffmann-La Roche Ltd. outside of this study.

For peer review only

Acknowledgements

CMcD, MDW, AAS, GG, SDD and LR acknowledge support from the National Institute for Health and Care Research (NIHR) Newcastle Biomedical Research Centre (BRC). The NIHR Newcastle BRC is a partnership between Newcastle Hospitals NHS Foundation Trust, Cumbria Northumberland Tyne and Wear NHS Foundation Trust, and Newcastle University. The views expressed are those of the authors and not necessarily those of the NIHR or the Department of Health and Social Care.

SDD and LR were also supported by the Mobilise-D project that has received funding from the Innovative Medicines Initiative 2 Joint Undertaking (JU) under grant agreement No. 820820. This JU receives support from the European Union's Horizon 2020 research and innovation program and the European Federation of Pharmaceutical Industries and Associations (EFPIA). SDD and LR were also supported by the Innovative Medicines Initiative 2 Joint Undertaking (IMI2 JU) project IDEA-FAST - Grant Agreement 853981. SDD and LR were supported by the NIHR/Wellcome Trust Clinical Research Facility (CRF) infrastructure at Newcastle upon Tyne Hospitals NHS Foundation Trust.

We acknowledge the contribution of our much-missed colleague Dr Richard Dodds, who sadly died before this work could be completed.

For peer review only

Tables

Table 1 Inclusion and Exclusion Criteria

INCLUSION CRITERIA
• Age 65 years or over<br>• Low maximum handgrip strength (<16kg for women, <27kg for men) OR five-times sit to stand time >15 seconds (inability to complete sit to stand test will count as time >15seconds)<br>• Walk speed <=0.8 m/s on 4 metre walk test
**EXCLUSION CRITERIA**
General:<br>• Allergy to acipimox or other niacin-related products<br>• Allergy or intolerance of aspirin<br>• Any contraindication to taking aspirin<br>• Unable to give written informed consent<br>• Currently enrolled in another intervention study (observational studies are permitted)<br>• Currently participating in supervised exercise classes or physiotherapy<br>• Any progressive neurological or malignant condition with life expectancy <6 months
Safety of investigations medications:<br>• Creatinine Clearance <45ml/min (by Cockcroft-Gault equation)<br>• Taking statin medication or fibrate medication<br>• Active peptic ulcer disease or dyspepsia
Safety of muscle biopsy and MRI:<br>• Platelets <100x10^9/L at screening (contraindication to muscle biopsy)<br>• Presence of a bleeding diathesis or use of oral or parenteral anticoagulant medication<br>• Antiplatelet agents other than low dose (75mg once daily) aspirin<br>• Contraindications to MRI scanning (mild claustrophobia is not a contraindication)<br>• Allergy to local anaesthetic (lidocaine)<br>• Unable to palpate vastus lateralis muscle to enable biopsy localisation
Other causes of skeletal myopathy:<br>• Liver function tests (bilirubin, ALT, alkaline phosphatase) > 3x ULN<br>• Symptomatic (NYHA class II-IV) chronic heart failure (diagnosed according to European Society of Cardiology guidelines)<br>• Severe COPD (GOLD stage IV)<br>• Known myositis or other established myopathy<br>• Self-reported weight loss of >10% in last 6 months (to exclude significant cachexia)<br>• Known uncontrolled thyrotoxicosis<br>• 7.5mg/day or greater prednisolone use (or equivalent)

For peer review only

References

1. Patel HP, Syddall HE, Jameson K, et al. Prevalence of sarcopenia in community-dwelling older people in the UK using the European Working Group on Sarcopenia in Older People (EWGSOP) definition: findings from the Hertfordshire Cohort Study (HCS). *Age and Ageing* 2013;42(3):378-84. doi: 10.1093/ageing/afs197
2. Cruz-Jentoft AJ, Landi F, Schneider SM, et al. Prevalence of and interventions for sarcopenia in ageing adults: a systematic review. Report of the International Sarcopenia Initiative (EWGSOP and IWGS). *Age and Ageing* 2014;43(6):748-59. doi: 10.1093/ageing/afu115
3. Lunt E, Ong T, Gordon AL, et al. The clinical usefulness of muscle mass and strength measures in older people: a systematic review. *Age and Ageing* 2021;50(1):88-95. doi: 10.1093/ageing/afaa123
4. Liu P, Hao QK, Hai S, et al. Sarcopenia as a predictor of all-cause mortality among community-dwelling older people: A systematic review and meta-analysis. *Maturitas* 2017;103:16-22. doi: 10.1016/j.maturitas.2017.04.007
5. Yeung SSY, Reijnierse EM, Pham VK, et al. Sarcopenia and its association with falls and fractures in older adults: A systematic review and meta-analysis. *Journal of Cachexia Sarcopenia and Muscle* 2019;10(3):485-500. doi: 10.1002/jcsm.12411
6. Pinedo-Villanueva R, Westbury LD, Syddall HE, et al. Health Care Costs Associated With Muscle Weakness: A UK Population-Based Estimate. *Calcified Tissue International* 2019;104(2):137-44. doi: 10.1007/s00223-018-0478-1
7. Cruz-Jentoft AJ, Bahat G, Bauer J, et al. Sarcopenia: revised European consensus on definition and diagnosis. *Age and ageing* 2019;48(1):16-31.
8. Bellanti F, Lo Buglio A, Vendemiale G. Mitochondrial Impairment in Sarcopenia. *Biology-Basel* 2021;10(1):31. doi: ARTN 31

10.3390/biology10010031
9. Boffoli D, Scacco SC, Vergari R, et al. Decline with Age of the Respiratory-Chain Activity in Human Skeletal-Muscle. *Biochimica Et Biophysica Acta-Molecular Basis of Disease* 1994;1226(1):73-82. doi: Doi 10.1016/0925-4439(94)90061-2
10. Coen PM, Musci RV, Hinkley JM, Miller BF. Mitochondria as a target for mitigating sarcopenia. *Frontiers in Physiology* 2019:1883.
11. Barker FJ, Hart A, Sayer AA, Witham MD. Effects of nicotinamide adenine dinucleotide precursors on measures of physical performance and physical frailty: A systematic review. *JCSM Clinical Reports* 2022;7(4):93-106.
12. Camacho-Pereira J, Tarragó MG, Chini CCS, et al. CD38 Dictates Age-Related NAD Decline and Mitochondrial Dysfunction through an SIRT3-Dependent Mechanism. *Cell Metabolism* 2016;23(6):1127-39. doi: 10.1016/j.cmet.2016.05.006
13. Migliavacca E, Tay SKH, Patel HP, et al. Mitochondrial oxidative capacity and NAD

biosynthesis are reduced in human sarcopenia across ethnicities. *Nature Communications* 2019;10(1):5808. doi: ARTN 5808

10.1038/s41467-019-13694-1 [published Online First: 20191220]
14. Van De Weijer T, Phielix E, Bilet L, et al. Evidence for a direct effect of the NAD+ precursor acipimox on muscle mitochondrial function in humans. *Diabetes* 2015;64(4):1193-201.
15. Gautier J, Pirnay F, Jandrain B, et al. Endogenous substrate oxidation during exercise and variations in breath 13CO2/12CO2. *Journal of Applied Physiology* 1993;74(1):133-38.

16. Head A, Jakeman P, Kendall M, et al. The impact of a short course of three lipid lowering drugs on fat oxidation during exercise in healthy volunteers. *Postgraduate medical journal* 1993;69(809):197-203.
17. Nellemann B, Søndergaard E, Jensen J, et al. Kinetics and utilization of lipid sources during acute exercise and acipimox. *American Journal of Physiology-Endocrinology and Metabolism* 2014;307(2):E199-E208.
18. Halbirk M, Nørrelund H, Møller N, et al. Suppression of circulating free fatty acids with acipimox in chronic heart failure patients changes whole body metabolism but does not affect cardiac function. *American Journal of Physiology-Heart and Circulatory Physiology* 2010;299(4):H1220-H25.
19. Makimura H, Stanley TL, Suresh C, et al. Metabolic Effects of Long-Term Reduction in Free Fatty Acids With Acipimox in Obesity: A Randomized Trial. *Journal of Clinical Endocrinology & Metabolism* 2016;101(3):1123-33. doi: 10.1210/jc.2015-3696
20. Abouhajar A, Alcock L, Bigirumurame T, et al. Acipimox in Mitochondrial Myopathy (AIMM): study protocol for a randomised, double-blinded, placebo-controlled, adaptive design trial of the efficacy of acipimox in adult patients with mitochondrial myopathy. *Trials* 2022;23(1):1-15.
21. Witham MD, Achison M, Aspray TJ, et al. Recruitment strategies for sarcopenia trials: lessons from the LACE randomized controlled trial. *JCSM Rapid Communications* 2021;4(2):93-102. doi: 10.1002/rco2.38
22. Abellan Van Kan G, Rolland Y, Andrieu S, et al. Gait speed at usual pace as a predictor of adverse outcomes in community-dwelling older people an International Academy on Nutrition and Aging (IANA) Task Force. *The journal of nutrition, health & aging* 2010;13(10):881-89. doi: 10.1007/s12603-009-0246-z
23. Cockcroft DW, Gault MH. Prediction of Creatinine Clearance from Serum Creatinine. *Nephron* 1976;16(1):31-41. doi: Doi 10.1159/000180580
24. Patel HP, Syddall HE, Martin HJ, et al. The Feasibility and Acceptability of Muscle Biopsy in Epidemiological Studies: Findings from the Hertfordshire Sarcopenia Study (Hss). *Journal of Nutrition Health & Aging* 2011;15(1):10-15. doi: DOI 10.1007/s12603-011-0006-8
25. Dodds RM, Hurst C, Hillman SJ, et al. Advancing our understanding of skeletal muscle across the lifecourse: Protocol for the MASS_Lifecourse study and characteristics of the first 80 participants. *Experimental Gerontology* 2022;166:111884. doi: ARTN 111884

10.1016/j.exger.2022.111884
26. Dodds RM, Davies K, Granic A, et al. Mitochondrial respiratory chain function and content are preserved in the skeletal muscle of active very old men and women. *Experimental Gerontology* 2018;113:80-85. doi: 10.1016/j.exger.2018.09.020
27. Strehler BL. Adenosine-5'-triphosphate and creatine phosphate determination with luciferase. Methods of enzymatic analysis: Elsevier 1974:2112-26.
28. Old SL, Johnson MA. Methods of Microphotometric Assay of Succinate-Dehydrogenase and Cytochrome-C Oxidase Activities for Use on Human Skeletal-Muscle. *Histochemical Journal* 1989;21(9-10):545-55. doi: Doi 10.1007/Bf01753355
29. Rocha MC, Grady JP, Grünewald A, et al. A novel immunofluorescent assay to investigate oxidative phosphorylation deficiency in mitochondrial myopathy: understanding mechanisms and improving diagnosis. *Scientific Reports* 2015;5(1):15037. doi: ARTN 15037

10.1038/srep15037
30. Grady JP, Murphy JL, Blakely EL, et al. Accurate Measurement of Mitochondrial DNA Deletion Level and Copy Number Differences in Human Skeletal Muscle. *Plos One* 2014;9(12):e114462. doi: ARTN e114462

10.1371/journal.pone.0114462

For peer review only

31. Roberts HC, Denison HJ, Martin HJ, et al. A review of the measurement of grip strength in clinical and epidemiological studies: towards a standardised approach. *Age and Ageing* 2011;40(4):423-29. doi: 10.1093/ageing/afr051
32. Guralnik JM, Simonsick EM, Ferrucci L, et al. A Short Physical Performance Battery Assessing Lower-Extremity Function - Association with Self-Reported Disability and Prediction of Mortality and Nursing-Home Admission. *Journals of Gerontology* 1994;49(2):M85-M94. doi: DOI 10.1093/geronj/49.2.M85
33. Axivity. AX6 Datasheet 6-Axis logging movement sensor 2023 [Available from: https://axivity.com/files/resources/AX6_Data_Sheet.pdf.
34. Del Din S, Galna B, Godfrey A, et al. Analysis of free-living gait in older adults with and without Parkinson's disease and with and without a history of falls: identifying generic and disease-specific characteristics. *The Journals of Gerontology: Series A* 2019;74(4):500-06.
35. Del Din S, Galna B, Lord S, et al. Falls risk in relation to activity exposure in high-risk older adults. *The Journals of Gerontology: Series A* 2020;75(6):1198-205.
36. Hollingsworth KG, Newton JL, Taylor R, et al. Pilot study of peripheral muscle function in primary biliary cirrhosis: Potential implications for fatigue pathogenesis. *Clinical Gastroenterology and Hepatology* 2008;6(9):1041-48. doi: 10.1016/j.cgh.2008.04.013 [published Online First: 20080808]
37. Loughran T, Higgins DM, McCallum M, et al. Improving highly accelerated fat fraction measurements for clinical trials in muscular dystrophy: origin and quantitative effect of R2* changes. *Radiology* 2015;275(2):570-78.
38. Burakiewicz J, Sinclair CDJ, Fischer D, et al. Quantifying fat replacement of muscle by quantitative MRI in muscular dystrophy. *Journal of Neurology* 2017;264(10):2053-67. doi: 10.1007/s00415-017-8547-3 [published Online First: 20170701]
39. Birkbeck MG, Blamire AM, Whittaker RG, et al. The role of novel motor unit magnetic resonance imaging to investigate motor unit activity in ageing skeletal muscle. *Journal of cachexia, sarcopenia and muscle* 2021;12(1):17-29.
40. Malmstrom TK, Morley JE. SARC-F: A Simple Questionnaire to Rapidly Diagnose Sarcopenia. *Journal of the American Medical Directors Association* 2013;14(8):531-32. doi: 10.1016/j.jamda.2013.05.018
41. Dodds RM, Murray JC, Robinson SM, Sayer AA. The identification of probable sarcopenia in early old age based on the SARC-F tool and clinical suspicion: findings from the 1946 British birth cohort. *European Geriatric Medicine* 2020;11(3):433-41. doi: 10.1007/s41999-020-00310-5 [published Online First: 20200319]

For peer review only

Supplementary Table 1 Schedule of events

	**Visit 1**	**Visit 2**	**Visit 3**	**Visit 4***	**Visit 5**	**Visit 6**	**Visit 7**	**Visit 8**	**Visit 9**	**Visit 10**
	**Pre-Screen**	**Screening**	**Baseline 1**	**Baseline 2**	**Phone call**	**Phone call**	**Follow up 1**	**Follow up 2**	**Phone call**	**Phone call**
**Assessment**		**Day -28 to -1**	**Day 0**	**Day 7 (+/- 1 day)**	**Day 9 (+/- 1 day)**	**Day 14 (+/- 2 days)**	**Day 21 (+/- 3 days)**	**Day 28 (+/- 3 days)**	**Day 30 (+/- 3 days)**	**Day 37 (+/- 4 days)**
SARC-F questions	X									
Demographics	X	X								
Medical history	X	X								
Concomitant medications	X	X	X	X			X	X		
Informed written consent		X								
Height		X								
Weight		X								
Bloods (U+Es, liver function tests)		X								
Bloods (Full blood count)		X								
Bloods (research bloods for NAD levels)			X				X			
Frailty questions (activity, exhaustion, weight loss)			X				X			
Muscle biopsy				X				X		
MRI scanning			X				X			

For peer review only

Accelerometer given out			X				X			
Accelerometer returned				X				X		
Medication dispensing				X						
Instructions on taking medication					X				X	
Medication adherence (collection/count)								X		
Telephone check of wound healing					X	X			X	X
Adverse Events log completion		X	X	X	X	X	X	X	X	X
4m walk speed		X								
5-times sit to stand		X								
Grip strength		X	X				X			
Short Physical Performance Battery			X				X			
Eligibility confirmation		X								

For peer review only

For peer review only

Participant ID: A C - __ __ __

Acipimox to improve muscle function and sarcopenia – a feasibility study

EudraCT 2021-000993-28 Sponsor ref: 09768

Prescreening form

Centre/Participant ID: | A | C | – | | | |

For peer review only

PRESCREENING CONTACT

Date of prescreening contact | d | d | m | m | y | y | y | y |

Contact by: Telephone ☐ Face to face ☐

Age (years) ☐☐

Sex: Male ☐ Female ☐

SARC-F:

	Score
How much difficulty do you have in lifting and carrying 10 pounds? *(none=0, some=1, a lot or unable without help=2)*	
How much difficulty do you have walking across a room? *(none=0, some=1, a lot or unable without help=2)*	
How much difficulty do you have transferring from a chair or bed? *(none=0, some=1, a lot or unable without help=2)*	
How much difficulty do you have climbing a flight of 10 stairs? *(none=0, some=1, a lot or unable without help=2)*	
How many times have you fallen in the past year? *(none=0, 1-3 = 1, 4 or more = 2)*	
**Total score**	

For peer review only - http://bmjopen.bmj.com/site/about/guidelines.xhtml

Participant ID: A C - __ __ __

Active medical diagnoses

	Yes	No
Chronic heart failure		
Severe COPD (GOLD stage IV)		
Progressive neurological disease with life expectancy <6 mths		
Cancer with life expectancy <6 mths		
Myositis or other myopathy		
Active thyrotoxicosis		

Concomitant medications

	Yes	No
Statin		
Fibrate		
Acipimox		
Niacin		
>7.5mg/day oral prednisolone or equivalent		
Antiplatelet other than aspirin 75mg once daily		
Anticoagulants		
Previous intolerance of acipimox or niacin		

Weight (kg) [ ][ ].[ ]

Weight change in last 6 months (to nearest kg) (NB: 1 stone = 6kg): [+/-][ ][ ]

For peer review only - http://bmjopen.bmj.com/site/about/guidelines.xhtml

Participant ID: A C - __ __ __

Other eligibility criteria:

	Yes	No
Contraindications to MRI		
>10% weight loss over last 6 months		
Unwilling to have two muscle biopsies		

	Yes	No
Eligible to proceed in study?		
Participant happy for study team to access medical records?		
Participant happy to receive PIS?		
PIS sent?		

For peer review only

Research nurse / investigator signature: ___________________________ Date: _ _ / _ _ /_ _ _ _

For peer review only - http://bmjopen.bmj.com/site/about/guidelines.xhtml

The Newcastle upon Tyne Hospitals NHS Foundation Trust

Acipimox to improve muscle function and sarcopenia – a feasibility study

Introduction

Thank you for considering taking part in the acipimox study. Before you decide whether to take part, please take time to read this information about the study carefully. Feel free to discuss it with your family, friends, carers or your GP if you wish to do so. If anything is unclear, or you need more information, please ask. You will find our contact details at the back of the information. This leaflet is yours to keep.

For peer review only

SUMMARY of the ACIPIMOX STUDY

**Purpose of the study**	• We would like to find out whether a medicine called acipimox (used to treat high cholesterol) can help to improve muscle function for people over the age of 65 with muscle weakness.
**Will I definitely get acipimox?**	• Yes. Everyone in the study will receive acipimox – no dummy (placebo) medication is used in this study.
**What are the side effects of the medicine?**	• Acipimox may cause some people to feel hot and red in the face (flushing) or cause a headache at the start of treatment but this usually goes away quickly. We will give you a mini-aspirin each day to stop this side-effect. Acipimox can cause nausea or indigestion in some people. A full list of side-effects are given later in this information sheet under Supporting Information
**If I take part, what will I have to do?**	• All of the study visits will take place in a research centre (the Clinical Ageing Research Unit)<br>• You will be asked to attend a screening visit. The doctor will discuss the study with you and you can ask questions. We will ask to take a small blood sample to see if it is safe for you to take part.

For peer review only - http://bmjopen.bmj.com/site/about/guidelines.xhtml

Newcastle University

NHS The Newcastle upon Tyne Hospitals NHS Foundation Trust

- If the study is suitable for you, we will ask you to take part in another four visits.
- We will ask you to take a tablet (acipimox) 2-3 times a day for 18 days together with aspirin once a day.
- At two of the visits, we will take a small sample of muscle tissue (a biopsy) from your thigh
- At two of the visits, we will do an MRI scan of your leg some of the scan will be at rest and other parts will be whilst you exercise your leg muscle in the scanner
- At these visits we will also ask you to do some simple tests of muscle strength and take a blood sample We will ask you to give small blood sample (a tablespoons worth)
- We will ask you to wear a sensor to measure your walking for a week before and after taking the course of tablets

**Do I have to visit the hospital to take part?**	• The study visits are at a research centre which is separate from the hospital. For this study you do need to come to the research centre.
**Do I have to take part?**	• No – it is entirely your choice whether you wish to take part or not. Your care will not be affected in any way.

If you are still interested in taking part in the study, please read on for more information.

For peer review only - http://bmjopen.bmj.com/site/about/guidelines.xhtml

Newcastle University

The Newcastle upon Tyne Hospitals NHS Foundation Trust

What is the purpose of the study?

Many of us lose muscle size and strength as we get older – this is called sarcopenia. People with weaker muscles are more likely to fall over and may start to have problems carrying out normal daily activities. They may also take longer to recover from other illnesses. Falling, struggling with daily living and taking a long time to get better from being unwell is a condition called frailty.

The best way to keep up your muscle strength is to do strengthening exercises, but not everyone wants to, or is able to do these.

Acipimox is a medicine that is already safely used in older people to treat high cholesterol. Recent research suggests that levels of a naturally occurring substance called Nicotinamide Adenine Dinucleotide (NAD) are low in the muscles of people with sarcopenia. All cells in the body need NAD to produce and use energy. We think that acipimox might be able to increase the levels of NAD in muscle, which could in turn help muscles to work better for people with sarcopenia. To know if this is correct, we need to test the effects of acipimox on muscle function in people with signs of muscle weakness.

What are the possible side effects of the study medicine?

As with any medicine, the medicine used in this study (**acipimox**) may cause side effects in some people. Doctors around the world have used acipimox for nearly 40 years and the side effects are well understood.

In some people, acipimox can cause flushing (redness of the face) and headache. This side effect usually affects people for the first few doses but then gets better. To minimise this side effect, we will give you a low dose of aspirin to take once a day (if you already take aspirin, you will just keep taking your normal dose of aspirin). Less commonly, acipimox can cause nausea or indigestion. To help prevent this, we will ask you to take your study medicine during or just after meals.

Aspirin can cause inflammation of the stomach lining, and in rare cases can cause stomach ulcers. We are using a low dose of aspirin over a short period of time to minimise side effects, but if you have indigestion, stomach ulcers or are prone to bleeding, we will not include you in the study.

For peer review only - http://bmjopen.bmj.com/site/about/guidelines.xhtml

The 28-day follow-up visit is the last study visit. For the 28 days following on from this final visit, we would like to know if you have been unwell. We will ask you to contact your study doctor or a member of their team to tell them about this. You can contact your study doctor and team on the contact details given on the last page of this information sheet.

If you are unsure whether the study doctor needs to know about an illness or not, please get in touch with your study doctor or a member of their team to check.

What are the benefits and disadvantages of taking part in this study?

We cannot promise the study will help you directly. However, the information we collect from this study may help to improve the treatment for older people with muscle weakness. If you want to find out more about taking part in research studies, you can visit the NHS Choices website www.nhs.uk.

If you do get a side effect from the study medicine, your doctor can stop your medicine. The side effects should disappear rapidly.

The muscle biopsy will be taken after your skin and surrounding area is numbed by injecting local anaesthetic. The injection and biopsy may cause some minor discomfort and bruising after biopsy and may be uncomfortable for a few days. Muscle biopsy is a simple procedure removing only a small amount of muscle with a low risk of complications. In very rare cases permanent muscle weakness, infection or a patch of numbness might occur. See page 7 for more information.

Is it safe for me to take part in this study during COVID-19?

The study will only take place if Newcastle Hospitals says that it is safe to do so. The study team will follow all of the hospital COVID-19 policies and procedures when you visit, including wearing masks, gloves and visors. These will be explained to you when your appointments are arranged. The visits will take place at the Clinical Ageing Research Unit, which is on the old Newcastle General Hospital site. This means that you will not have to mix with other patients in the main hospitals.

For peer review only - http://bmjopen.bmj.com/site/about/guidelines.xhtml

What would I have to do if I took part?

We will ask you to attend five visits at the Clinical Ageing Research Unit, on the old Newcastle General Hospital site:

- Screening visit
- First baseline visit (on day 0)
- Second baseline visit (on day 7)
- First follow-up visit (on day 21)
- Second follow-up visit (on day 28)

These visits are extra, and not part of your usual NHS care. There is a flow diagram (Figure 1) on page 10 that shows the details of the trial visits.

Screening Visit

At the screening visit the doctor will discuss the study with you, and you will be able to ask questions. This visit may take place in your home rather than in the research unit if you prefer. We will ask if you are happy to give written consent to take part in the study. We will ask you some questions about yourself, your medications and your health. We will also ask to take a small blood sample (2 teaspoons) to make sure that it is safe for you to take part in this study. We will time you walking a short distance (4 metres), ask you to stand and sit from a chair five times, and test your handgrip strength.

When we have the results of the blood test, we will call you and arrange the next visit.

Baseline Visit 1

At this first visit, we will do the following:

- Measure your weight and height.
- Time you walking a short distance (4 metres), with your usual walking aids.
- To test your balance, we will ask you to stand beside the researcher for a short time (no more than 30 seconds).

For peer review only - http://bmjopen.bmj.com/site/about/guidelines.xhtml

- Time you standing and sitting from a chair five times.
- Test the strength of your handgrip on both hands.
- Ask you questions about your activity level, weight change and levels of tiredness.
- Take a small amount of blood (6 teaspoons).
- Ask about other medicine that you may be taking.

We will then perform a magnetic resonance imaging (MRI) scan of your leg muscles whilst you exercise your leg.

The Magnetic Resonance Imaging (MRI) scan

You will need to arrive at the MRI centre 30 minutes before your arranged scan. Before your scan you will be asked questions to ensure that there are no known reasons why you should not undergo a scan, for example if you have a pacemaker fitted. For your scan you will be asked to remove items of jewellery, clothing with metal and all other metal (such as hair grips). For this reason you may prefer to leave valuable items such as jewellery at home, however lockers are available. You may also prefer to arrive at the MRI centre wearing loose fitting clothing, although if you prefer to change into comfortable clothing at the clinic changing rooms are available.

When you go through the MRI scanner, you will be asked to lie down on the scanner bed. We slide the bed into the scanner up to chest height and collect images of your thigh muscle. You will be asked to keep as still as possible while we do this so that we get good images. The scanner is noisy but we will provide earplugs. Each of these scans lasts about 1 minute.

We will then move the table so that your calf is in the middle of the scanner. We will collect some images of your calf muscles at rest, again you will be asked to keep as still as possible. We will then ask you to push against a pedal (an up down motion) for about 3 minutes. The effort required is similar to walking uphill on Dean Street (a steep hill in Newcastle). The radiographer will then ask you to stop pushing and keep still for about 6 minutes while we collect data from your leg muscle after the exercise. After this, your MRI scan is complete.

When having a scan, there is always a small possibility that an abnormality could be observed on the images of which you and your doctors were unaware. The MRI scans we collect will be reviewed by a radiologist in the

For peer review only - http://bmjopen.bmj.com/site/about/guidelines.xhtml

Newcastle upon Tyne Hospitals NHS Foundation Trust to look for any such findings.

It is important to recognise that the MRI scans are not being taken for diagnostic purposes and so there is no guarantee that the scans would be of the right kind to detect any abnormality which may be present.

Should the radiologist suspect anything abnormal on your scans they will inform the study Principal Investigator who will contact your clinical care team or GP in order to make recommendations about any further investigations which may be appropriate for them to arrange.

The walking sensor

After the scan, we will give you the sensor to wear. This is small, lightweight and is worn on the lower back. You will need to wear this all the time for the next seven days until you return for the second baseline visit. You do not need to take it off at night, and you can wear it in the shower. It will monitor your walking activity.

Baseline visit 2

At the second baseline visit, which will take place 7 days after the first visit, we will collect the accelerometer and then do a muscle biopsy. If you usually take aspirin we will ask you not to take this on the morning of your muscle biopsy.

The muscle biopsy:

This test involves taking a small tissue sample from one of your thigh muscles. You will meet with an experienced doctor who will explain about the muscle biopsy and check that you are willing to have this procedure performed. Throughout the study you are free to change your mind about taking part in any part of the study without having to give a reason why.

If you are willing to go ahead, the doctor will ask you questions about what medication you are currently taking, your general health and examine you. This is because if, for example, you had varicose veins over the site of where we would take the biopsy, or were taking medications that can cause bleeding, then it would not be safe to perform this and therefore you would not undergo this part of the study.

For peer review only - http://bmjopen.bmj.com/site/about/guidelines.xhtml

Muscle biopsy is a simple procedure removing only a small amount of muscle with a low risk of complications. One in a hundred people experience significant bleeding that may leave you with a bruise taking a few weeks to disappear. In very rare cases permanent muscle weakness, infection or a patch of numbness (one in a thousand people) might occur.

The doctor and the nurse helping with the muscle biopsy will take you to a treatment room, set up to take a small tissue sample (biopsy) from your thigh muscle.

The procedure takes no longer than 30 minutes. Whilst lying down, you will be given a local anaesthetic injection to numb the skin and surrounding area. When the area is numb, a small cut is made in the skin to allow the biopsy to be taken. The muscle biopsy will be done with a special type of forceps as shown in the photo, below. The local anaesthetic may cause a stinging sensation for a few seconds and you may feel a pulling sensation when the biopsy is taken.

The wound is so small that it will not require stitching but is closed using medical tape called "Steri-strips". A plaster will then be applied to the area, and a tight bandage wrapped around your leg for two hours whilst you rest on a bed. This is done to reduce the chance of bruising and bleeding.

The biopsy site may be uncomfortable for a few days, if so, paracetamol or codeine is recommended for pain relief. Anti-inflammatory drugs such as aspirin or ibuprofen should be avoided in the first 48 hours. You should also avoid getting the area wet in the first 48 hours. The Steri-strips can be removed after 5 days.

You should not drive after the biopsy and again we will provide a taxi to take you home. You will be given an advice sheet informing you to avoid strenuous activities like running or weightlifting for the first 5 days after your biopsy as this may delay the healing process and result in bleeding. However, the biopsy should not limit routine household tasks.

Follow-up phone calls

A member of the study team will telephone you twice as follow-up: the first time one or two days after your muscle biopsy and the second time around a week later to ensure that you are well. They will check with you that the wound from the muscle biopsy has healed. If you have any concern at all

For peer review only - http://bmjopen.bmj.com/site/about/guidelines.xhtml

about the site of the muscle biopsy, then a member of the study team will arrange to visit you at home.

The study medication

At the end of this second baseline visit, we will give you the study medicines to take home with you. **Please wait until two days after the biopsy** before you take the first doses of medication; we will telephone you first to make sure that the biopsy wound is healing as it should before starting the medication.

Follow up visit 1

At the first follow-up visit, which will take place two weeks later, we will repeat all the tests (including the MRI scan) which we did at the first baseline visit.

Follow up visit 2

At the second follow-up visit, which will take place seven days after the first follow-up visit, we will collect the accelerometer and your study medications. We will then do the muscle biopsy again, and as before we will telephone you a couple of days later and then a week later to check that everything has healed without problems.

A summary of the study visits is shown in a flow diagram (Figure 1) on the next page.

For peer review only - http://bmjopen.bmj.com/site/about/guidelines.xhtml

Newcastle University

The Newcastle upon Tyne Hospitals NHS Foundation Trust

Figure 1. Summary of acipimox study visits

Screening visit – 60 mins
Written informed consent with doctor
Medical history, other medicines
Physical tests (short walk, sit to stand and handgrip strength)
Blood sample (2 teaspoons)
After visit - eligibility for the study confirmed

Day 0: Baseline visit 1 - two hours
Height and weight
Physical tests (walking, balance, sit to stand and handgrip strength)
Questions on activity and tiredness
Blood sample (6 teaspoons)
Review of other medication and any episodes of ill health
MRI scan of leg
Start wearing walking sensor

Day 7: Baseline visit 2 – three hours (includes 2 hours rest after the biopsy)
Medicines review and review of any episodes of ill health
Return walking sensor
Muscle Biopsy
Receive study medication
After visit: telephone call at 48 hours and 7 days. Start taking medication at 48 hours

Day 21: Follow up visit 1 - two hours
Physical tests (walking, balance, sit to stand and handgrip strength)
Questions on activity and tiredness
Blood sample (6 teaspoons)
Review of other medication and any episodes of ill health
MRI scan of leg
Start wearing walking sensor

Day 28: Follow up visit 2 – three hours (includes 2 hours rest after the biopsy)
Medicines review and review of any episodes of ill health
Return walking sensor and study medication
Muscle Biopsy
After visit: telephone call at 48 hours and 7 days

For peer review only - http://bmjopen.bmj.com/site/about/guidelines.xhtml

What happens to my blood and muscle samples?

At the baseline and follow up visits, we take blood and muscle samples to analyse as part of the study. If there is any sample left over from these analyses, we ask your permission to store it in a licenced Newcastle University Biobank. Your consent to store these additional samples in the biobank is optional. Your biobank samples may be used in further research linked to this study. They may also be used by other researchers in different research studies. Your samples will be identified only using your unique study identity number. Researchers who use your samples will not know who you are. Other researchers may want to use parts of your biobank sample in animal or commercial (paid) research. You can give samples to the biobank but opt out of them being used for animal or paid research.

Pregnancy

We know that it is not possible for female participants over the age of 65 years to become pregnant.

However, if you are a male participant, we will ask you to inform us if your female partner becomes pregnant, or is breast-feeding an infant. We will ask for your consent to do this. We will also ask your partner to sign a consent form. This will allow the study team to collect safety information about their pregnancy and their baby.

What happens at the end of the study?

At the end of the study (4 weeks) you will stop taking the study medicines. You will continue to receive standard care like any other patient with your condition under the care of your GP and/or hospital doctor. If you were taking aspirin before the study, you will continue to take this as before.

When everyone has completed the study, we will analyse the results and we will tell you what the results are. We can either invite you to a study event to which we will invite you, or send you a written newsletter – whichever you prefer.

For peer review only - http://bmjopen.bmj.com/site/about/guidelines.xhtml

Who do I contact for further information?

We will be happy to answer any questions you, your family or your carers may have about any aspect of this clinical study. Please call the number at the end of this booklet to speak to the research team.

For peer review only

For peer review only - http://bmjopen.bmj.com/site/about/guidelines.xhtml

Newcastle University

The Newcastle upon Tyne Hospitals NHS Foundation Trust

SUPPORTING INFORMATION

Acipimox side effects

Very common – Flushing, headache, indigestion

Common – Stomach ache, tiredness, rash

Uncommon – Allergic reaction, rash or itching, wheezing, nausea, feeling hot or unwell, muscle aches

Other – loose stools, gritty eyes

Acipimox may cause a sensation of heat, flushing or itching, especially at the beginning of treatment; it may cause a rash or redness of the skin. These reactions usually disappear rapidly during the first day of treatment.

Why was I contacted?

We have contacted you because you are aged 65 or over and our measurements or questionnaire results suggest that your muscles are not as strong as they used to be. This trial is testing a medicine (acipimox) to see if we can prevent further weakening of muscles in people like you.

Do I have to take part?

No - it is up to you to decide whether or not to take part in this study. You do not have to take part. If you choose not to, you will continue to get the standard care arranged by your doctor.

If you agree to take part, we will ask you to sign a consent form. We will give you a copy of your signed consent form which is yours to keep. You can still change your mind and withdraw at any time without having to give a reason. If you decide not to take part, or withdraw from the study later on, your current or future medical care will not be affected in any way.

What does giving consent mean for me?

By signing a consent form, this means that you fully understand what taking part in the study means for you. That's why it is really important that you take as much time as you want to read this information sheet. Feel free to discuss the study with your family, friends or any healthcare professional. At the screening visit, you will be discuss the study with a study doctor, who will answer all of your questions.

For peer review only - http://bmjopen.bmj.com/site/about/guidelines.xhtml

Newcastle University

The Newcastle upon Tyne Hospitals NHS Foundation Trust

Will I know what treatment I am on?

Yes. Everyone in the study will receive acipimox – no dummy (placebo) medication is used in this study.

Who has checked the scientific quality of the study?

Independent experts at Newcastle University have checked the quality of the science used to plan this study. The study has also been checked and approved by the app committee ensures that when you take part in the study, your rights and wellbeing will be protected. The study has also been checked by the government Medicines and Healthcare products Regulatory Agency (MHRA). The MHRA are responsible for approving all studies involving medicines. The Health Research Authority gives final overall approval for the study. The Newcastle upon Tyne Hospitals NHS Foundation Trust is the study Sponsor, which means that they have overall responsibility for the study. The Sponsor has carefully checked all of the study documentation. The Sponsor has also assessed the risks of this study. This is to ensure that we are not doing anything harmful to you during the study and that your information is collected safely and stored securely.

What happens if relevant new information becomes available?

During the course of the study, if new information on the risks or benefits of acipimox becomes available, we will let you know at your next study visit. If this new information requires urgent action, we will contact you before your next visit. If necessary, we will then discuss whether you should or would like to withdraw from the study.

Will you tell my GP that I'm taking part in a clinical study?

Yes. With your permission, we will write to your GP to tell them that you are taking part in this study. Your hospital medical record will also show that you are taking part in a clinical study. It is important for your safety that that your GP practice and hospital medical records show that you took part in a clinical study. If we discover a new health problem during the study, we will tell you. With your permission, we will also tell your GP. Any blood test results from taking part in this study will also be added to your medical records. Your GP will be asked to let the study team know of any side effects from taking the medicine or if you have had any emergency hospital admissions.

For peer review only - http://bmjopen.bmj.com/site/about/guidelines.xhtml

Newcastle University

The Newcastle upon Tyne Hospitals NHS Foundation Trust

Who has overall responsibility for the study?

The study Sponsor is the Newcastle upon Tyne Hospitals NHS Foundation Trust, who have overall responsibility for the study.

The doctor in charge of the study (the Chief Investigator) is Professor Miles Witham, a Consultant Geriatrician based in Newcastle upon Tyne.

Who is providing the study medications?

Acipimox and aspirin have been on the market for many years. We have bought the study medications from their manufacturers in the same way that the NHS would usually buy medications – the medications have not been supplied as a favour by the manufacturers.

What will happen to the results of the Acipimox study?

- The results will be published in medical journals and presented at meetings to other doctors, nurses, researchers and patients.
- A report will be written for the study funder.
- A report will be written for the Controlled Clinical Trials (ISRCTN) database.
- All study results that are published will be anonymous. This means that no-one will be able to find out who you are. Your identity will always be protected.
- The results will be available at the end of the study through publications, in the wider press and directly to patient groups.
- Fully anonymised data may be made available to other researchers to help inform other research studies.

What if I have a complaint or a problem occurs?

a) Complaints

If you have any concern or complaint about any aspect of this clinical study, please contact your local study team by phone, letter or email. Their contact details are listed at the end of this information sheet. If you are still unhappy and wish to raise your concerns with someone who is not directly involved in your care, you can contact Patient Advice and Liaison Service (PALS) for

For peer review only - http://bmjopen.bmj.com/site/about/guidelines.xhtml

confidential advice on any aspect of care on 0800 032 0202, or email northoftynepals@nhct.nhs.uk

b) Harm

In the unlikely event that you are harmed during the study and this is due to NHS staff neglect, you may have grounds for legal action and compensation. This is organised through the NHS Indemnity (insurance) scheme. You may need to pay for your own legal costs. NHS Indemnity does not offer no-fault compensation (for harm that is not anyone's fault).

Newcastle University also have indemnity arrangements. This covers Newcastle University staff involved in designing and managing the Acipimox study.

Will my taking part in the study be kept confidential?

Yes. All the information that you provide during the course of this study will be securely stored. Paper copies of your study information will be stored in locked files or rooms at your local hospital. Electronic copies of your study information will be stored on a secure, password-protected computer database. Only authorised members of the study team will be granted access to the database.

- At study visits, your name will not be written on completed test forms or questionnaires. Instead, we will use a study code number (called a Participant Unique Study Identifier). This number is unique for you. No-one else taking part in the study will have this number. This number will also be used in the study database. Only the study team at your hospital will be able to link this number back to you using your date of birth, name and NHS number
- The study team at your hospital will have access to your information during the study. They will use this information to contact you to organise study visits as well as for ongoing safety.
- You will not be named in any results, reports or on websites
- Very occasionally, information might be given during the study that, by law, we must pass on to others. For instance, information which suggested you or others were at risk of harm. In this case, confidentiality

For peer review only - http://bmjopen.bmj.com/site/about/guidelines.xhtml

would be broken so that we could pass this information to the relevant people. You would be informed of this.

- At the end of the study, all study information will be kept in a secure storage area for at least 15 years. This is called archiving. Archiving means that any queries about the running of the study can still be answered after the study has ended. All information will be held securely to make sure we protect your confidentiality. After the archiving period has ended, your information will be safely destroyed.
- If there are any unexpected serious side effects to the medicine, we would send details of this to the government medicines agency (MHRA). There is a specific form to do this, and only your study number will be sent to them.

Will you look at information from my existing medical records?

Yes. The study team at your hospital will be able to look at your GP and hospital medical records. They need to do this to collect information that is needed for you to take part in the study. For example, they will collect results of your blood tests, and prescriptions and health history.

Authorised people from Newcastle Hospitals NHS Foundation Trust or from the MHRA will also need to look at your medical records. This is to check that the study is being carried out to the correct standards. Everyone who looks at your medical records will have a duty of confidentiality to you as a research participant.

What will happen if I don't want to carry on with the study?

You can withdraw from the study completely at any time, for any reason. You do not have to tell the study team why you want to withdraw. You will always be fully cared for and supported in line with your GP and hospital's standard practice.
If you do give a reason for withdrawing from the study, we will ask if you are happy for us to record why you decided to withdraw.

If you withdraw from the study, we will keep the information about you that we have already collected. You are free to request that the study team

For peer review only - http://bmjopen.bmj.com/site/about/guidelines.xhtml

Newcastle University

The Newcastle upon Tyne Hospitals NHS Foundation Trust

destroys all information donated by you. By destroying your information, this means that it cannot be used at all for the remainder of the study. However, if some of your information has already been used in calculations and reports, it would not be possible to remove that information from these.

If I stop taking the medicine, do I have to leave the trial?

If you become unwell or the medicine causes side effects that you cannot tolerate, your usual doctor or the study team may ask you to stop taking the study medicine. If you stop taking the medication, you will not continue in the trial and will not have the second set of scans, blood tests and muscle biopsy.

What happens if I lose the capacity to consent during the study?

During the study, if you lose the capacity to make your own decisions, we will stop your medicine and withdraw you from the study.

Who is funding the acipimox study?

The Medical Research Council are funding the study through money given to Newcastle University. The UK government funds the MRC to carry out research to improve knowledge of diseases, and treatments to improve health.

How have patients and the public been involved in the design of the study?

Volunteers from the local VOICE organisation (https://www.voice-global.org/) have given us feedback on designing studies for older people which we have used in designing this study and the information sheet.

Will my expenses be reimbursed?

Yes – we will pay for your travel expenses including providing a taxi if you need this to attend study visits at the hospital. Alternatively, transport may be arranged for you if your local hospital is able to offer this. The study team will manage any payments to reimburse costs to you and you may be asked to provide receipts for your travel.

Will I be paid for taking part?

For peer review only - http://bmjopen.bmj.com/site/about/guidelines.xhtml

We will not give you a payment for taking part, but we will pay for your transport and make sure that you have some food and drink at each trial visit.

HOW WILL WE USE INFORMATION ABOUT YOU?

We will need to use information from you, your medical records and your GP for this clinical research study.

This information will include your initials, date of birth, NHS number, name and contact details. People will use this information to do the research or to check your records to make sure that the research is being done properly.

People who do not need to know who you are will not be able to see your name or contact details. Your data will have a code number, called your unique study identifier, instead.

We will keep all information about you safe and secure.

Once we have finished the study, we will keep some of the data so we can check the results. We will write our reports in a way that no-one can work out that you took part in the study.

What are your choices about how your information is used?

- You can stop being part of the study at any time, without giving a reason, but we will keep information about you that we already have.
- We need to manage your records in specific ways for the research to be reliable. This means that we won't be able to let you see or change the data we hold about you.

Where can you find out more about how your information is used?

You can find out more about how we use your information:

- www.hra.nhs.uk/information-about-patients/
- our leaflet available from https://www.newcastle-hospitals.nhs.uk/help/privacy/

For peer review only - http://bmjopen.bmj.com/site/about/guidelines.xhtml

- by asking one of the study team
- by sending an email to the trial Sponsor Data Protection Officer at nuth.dpo@nhs.net

Further Information and contact details

If you have any further questions or would like further information about the study or rights of participants, please feel free to contact the people below.

They are also who you or a doctor should contact in the event of an emergency.

Professor Miles Witham, telephone 07555 450498

Clinical Ageing Research Unit, telephone 0191 208 1250

Thank you for reading this information sheet

This research is funded by the UK Medical Research Council through their Confidence in Concept funding stream. The views expressed are those of the author(s) and not necessarily those of the Medical Research Council.

For peer review only - http://bmjopen.bmj.com/site/about/guidelines.xhtml

Reporting checklist for protocol of a clinical trial.

Based on the SPIRIT guidelines.

Instructions to authors

Complete this checklist by entering the page numbers from your manuscript where readers will find each of the items listed below.

Your article may not currently address all the items on the checklist. Please modify your text to include the missing information. If you are certain that an item does not apply, please write "n/a" and provide a short explanation.

Upload your completed checklist as an extra file when you submit to a journal.

In your methods section, say that you used the SPIRITreporting guidelines, and cite them as:

Chan A-W, Tetzlaff JM, Gøtzsche PC, Altman DG, Mann H, Berlin J, Dickersin K, Hróbjartsson A, Schulz KF, Parulekar WR, Krleža-Jerić K, Laupacis A, Moher D. SPIRIT 2013 Explanation and Elaboration: Guidance for protocols of clinical trials. BMJ. 2013;346:e7586

		Reporting Item	Page Number
**Administrative information**			
Title	#1	Descriptive title identifying the study design, population, interventions, and, if applicable, trial acronym	1

For peer review only

Trial registration	#2a	Trial identifier and registry name. If not yet registered, name of intended registry	5
Trial registration: data set	#2b	All items from the World Health Organization Trial Registration Data Set	
Protocol version	#3	Date and version identifier	4 & 17
Funding	#4	Sources and types of financial, material, and other support	19
Roles and responsibilities: contributorship	#5a	Names, affiliations, and roles of protocol contributors	1-2, 19
Roles and responsibilities: sponsor contact information	#5b	Name and contact information for the trial sponsor	16
Roles and responsibilities: sponsor and funder	#5c	Role of study sponsor and funders, if any, in study design; collection, management, analysis, and interpretation of data; writing of the report; and the decision to submit the report for publication, including whether they will have ultimate authority over any of these activities	NA
Roles and responsibilities: committees	#5d	Composition, roles, and responsibilities of the coordinating centre, steering committee, endpoint adjudication committee, data management team, and	15

For peer review only

		other individuals or groups overseeing the trial, if applicable (see Item 21a for data monitoring committee)	
**Introduction**			
Background and rationale	#6a	Description of research question and justification for undertaking the trial, including summary of relevant studies (published and unpublished) examining benefits and harms for each intervention	6
Background and rationale: choice of comparators	#6b	Explanation for choice of comparators	NA
Objectives	#7	Specific objectives or hypotheses	8
Trial design	#8	Description of trial design including type of trial (eg, parallel group, crossover, factorial, single group), allocation ratio, and framework (eg, superiority, equivalence, non-inferiority, exploratory)	8
**Methods: Participants, interventions, and outcomes**			
Study setting	#9	Description of study settings (eg, community clinic, academic hospital) and list of countries where data will	8

For peer review only

		be collected. Reference to where list of study sites can be obtained	
Eligibility criteria	#10	Inclusion and exclusion criteria for participants. If applicable, eligibility criteria for study centres and individuals who will perform the interventions (eg, surgeons, psychotherapists)	9
Interventions: description	#11a	Interventions for each group with sufficient detail to allow replication, including how and when they will be administered	9
Interventions: modifications	#11b	Criteria for discontinuing or modifying allocated interventions for a given trial participant (eg, drug dose change in response to harms, participant request, or improving / worsening disease)	9
Interventions: adherance	#11c	Strategies to improve adherence to intervention protocols, and any procedures for monitoring adherence (eg, drug tablet return; laboratory tests)	22
Interventions: concomitant care	#11d	Relevant concomitant care and interventions that are permitted or prohibited during the trial	8&9
Outcomes	#12	Primary, secondary, and other outcomes, including the specific measurement variable (eg, systolic blood pressure), analysis metric (eg, change from baseline, final value, time to event), method of aggregation (eg, median, proportion), and time point for each outcome.	10-13

For peer review only

		Explanation of the clinical relevance of chosen efficacy and harm outcomes is strongly recommended	
Participant timeline	#13	Time schedule of enrolment, interventions (including any run-ins and washouts), assessments, and visits for participants. A schematic diagram is highly recommended (see Figure)	12-11
Sample size	#14	Estimated number of participants needed to achieve study objectives and how it was determined, including clinical and statistical assumptions supporting any sample size calculations	13-14
Recruitment	#15	Strategies for achieving adequate participant enrolment to reach target sample size	8
**Methods: Assignment of interventions (for controlled trials)**			
Allocation: sequence generation	#16a	Method of generating the allocation sequence (eg, computer-generated random numbers), and list of any factors for stratification. To reduce predictability of a random sequence, details of any planned restriction (eg, blocking) should be provided in a separate document that is unavailable to those who enrol participants or assign interventions	NA

For peer review only

Allocation concealment mechanism	#16b	Mechanism of implementing the allocation sequence (eg, central telephone; sequentially numbered, opaque, sealed envelopes), describing any steps to conceal the sequence until interventions are assigned	NA
Allocation: implementation	#16c	Who will generate the allocation sequence, who will enrol participants, and who will assign participants to interventions	NA
Blinding (masking)	#17a	Who will be blinded after assignment to interventions (eg, trial participants, care providers, outcome assessors, data analysts), and how	NA
Blinding (masking): emergency unblinding	#17b	If blinded, circumstances under which unblinding is permissible, and procedure for revealing a participant's allocated intervention during the trial	NA
**Methods: Data collection, management, and analysis**			
Data collection plan	#18a	Plans for assessment and collection of outcome, baseline, and other trial data, including any related processes to promote data quality (eg, duplicate measurements, training of assessors) and a description of study instruments (eg, questionnaires, laboratory tests) along with their reliability and validity,	12

For peer review only

		if known. Reference to where data collection forms can be found, if not in the protocol	
Data collection plan: retention	#18b	Plans to promote participant retention and complete follow-up, including list of any outcome data to be collected for participants who discontinue or deviate from intervention protocols	12
Data management	#19	Plans for data entry, coding, security, and storage, including any related processes to promote data quality (eg, double data entry; range checks for data values). Reference to where details of data management procedures can be found, if not in the protocol	12
Statistics: outcomes	#20a	Statistical methods for analysing primary and secondary outcomes. Reference to where other details of the statistical analysis plan can be found, if not in the protocol	14
Statistics: additional analyses	#20b	Methods for any additional analyses (eg, subgroup and adjusted analyses)	14
Statistics: analysis population and missing data	#20c	Definition of analysis population relating to protocol non-adherence (eg, as randomised analysis), and any statistical methods to handle missing data (eg, multiple imputation)	14
**Methods: Monitoring**			

For peer review only

Data monitoring: formal committee	#21a	Composition of data monitoring committee (DMC); summary of its role and reporting structure; statement of whether it is independent from the sponsor and competing interests; and reference to where further details about its charter can be found, if not in the protocol. Alternatively, an explanation of why a DMC is not needed	15
Data monitoring: interim analysis	#21b	Description of any interim analyses and stopping guidelines, including who will have access to these interim results and make the final decision to terminate the trial	15
Harms	#22	Plans for collecting, assessing, reporting, and managing solicited and spontaneously reported adverse events and other unintended effects of trial interventions or trial conduct	15
Auditing	#23	Frequency and procedures for auditing trial conduct, if any, and whether the process will be independent from investigators and the sponsor	15
**Ethics and dissemination**			
Research ethics approval	#24	Plans for seeking research ethics committee / institutional review board (REC / IRB) approval	16

For peer review only

Protocol amendments	#25	Plans for communicating important protocol modifications (eg, changes to eligibility criteria, outcomes, analyses) to relevant parties (eg, investigators, REC / IRBs, trial participants, trial registries, journals, regulators)	15
Consent or assent	#26a	Who will obtain informed consent or assent from potential trial participants or authorised surrogates, and how (see Item 32)	16
Consent or assent: ancillary studies	#26b	Additional consent provisions for collection and use of participant data and biological specimens in ancillary studies, if applicable	NA
Confidentiality	#27	How personal information about potential and enrolled participants will be collected, shared, and maintained in order to protect confidentiality before, during, and after the trial	11, 18 and ICF
Declaration of interests	#28	Financial and other competing interests for principal investigators for the overall trial and each study site	20
Data access	#29	Statement of who will have access to the final trial dataset, and disclosure of contractual agreements that limit such access for investigators	17
Ancillary and post trial care	#30	Provisions, if any, for ancillary and post-trial care, and for compensation to those who suffer harm from trial participation	16

For peer review only

Dissemination policy: trial results	#31a	Plans for investigators and sponsor to communicate trial results to participants, healthcare professionals, the public, and other relevant groups (eg, via publication, reporting in results databases, or other data sharing arrangements), including any publication restrictions	17
Dissemination policy: authorship	#31b	Authorship eligibility guidelines and any intended use of professional writers	16
Dissemination policy: reproducible research	#31c	Plans, if any, for granting public access to the full protocol, participant-level dataset, and statistical code	NA
**Appendices**			
Informed consent materials	#32	Model consent form and other related documentation given to participants and authorised surrogates	Separate attachment
Biological specimens	#33	Plans for collection, laboratory evaluation, and storage of biological specimens for genetic or molecular analysis in the current trial and for future use in ancillary studies, if applicable	9/10/11

None The SPIRIT Explanation and Elaboration paper is distributed under the terms of the Creative Commons Attribution License CC-BY-NC. This checklist can be completed online using https://www.goodreports.org/, a tool made by the EQUATOR Network in collaboration with Penelope.ai

For peer review only
